# Lung environment in healthy old age shapes the phenotype and CCR2-mediated recruitment of a subset of apoptotic, high-turnover alveolar macrophages

**DOI:** 10.1038/s41467-026-78156-x

**Published:** 2026-10-07

**Authors:** Susanta Pahari, Miranda Lumbreras, Arkajyoti Paul, Abul Azad, Hao Zhang, Hong Cai, Zhao Lai, Diego Jose Maselli, Jay Peters, Jordi B. Torrelles, Joanne Turner, Florent Ginhoux, William P. Lafuse, Yufeng Wang, Eusondia Arnett, Larry S. Schlesinger

**Affiliations:** 1 Host Pathogen Interactions and Population Health programs, International Center for the Advancement of Research & Education (I•CARE), Texas Biomedical Research Institute, San Antonio, TX USA; 2 Duke University School of Medicine, Durham, NC USA; 3 Department of Molecular Microbiology and Immunology, South Texas Center for Emerging Infectious diseases, University of Texas at San Antonio, San Antonio, TX USA; 4 Department of Molecular Medicine, UT Health San Antonio, San Antonio, TX USA; 5 Division of Pulmonary and Critical Care Medicine, UT Health Science Center, San Antonio, TX 78207 USA; 6 Integrated Research Center for Infectious Diseases, Division of Infectious Diseases, Department of Internal Medicine, and Infectious Diseases Institute, The Ohio State University, Columbus, OH 43210 USA; 7 Abigail Wexner Research Institute at Nationwide Children’s Hospital, 700 Children’s Drive, Columbus, OH 43205 USA; 8 Singapore Immunology Network (SIgN), Agency for Science, Technology and Research (A*STAR), 138648 Singapore, Singapore; 9 Department of Microbial Infection and Immunity, The Ohio State University, Columbus, OH 43210 USA

**Keywords:** Chronic inflammation, Alveolar macrophages, Apoptosis, Translational immunology, Ageing

## Abstract

Immune system changes with healthy aging lead to chronic systemic inflammation termed inflammaging, contributing to age-related pathologies. Alveolar macrophages (AMs) maintain lung homeostasis and health. The impact of inflammaging on AM populations requires further definition. Herein, we examined the effect of healthy old age on the phenotype and ontogeny of AMs from mice, non-human primates and humans. We identify three AM subpopulations in old age, including a CD11c^+^CD11b^+^ population that increases more than 10-fold across mammalian species, leading to significant changes associated with heightened inflammation and immune dysregulation. RNA-seq analysis identifies distinct transcriptional AM subpopulation profiles. Experiments in mice reveal the importance of the alveolar environment in shaping AM phenotype and recruitment in healthy old age. The old age alveolar environment promotes phenotypic reprogramming of tissue-resident AMs (TRAMs) toward a CD11c^+^CD11b^+^ state in addition to enhancing CCR2-dependent recruitment of monocyte-derived AMs, which are relatively short-lived and exhibit high turnover due to Fas-mediated apoptosis. These studies provide important insight regarding the impact of the alveolar environment in healthy old age on AM phenotype and function.

## Abstract (web summary)

Immune system changes in healthy aging are implicated in systemic inflammation known as ‘inflammaging’. Here the authors examine the phenotype and function of alveolar macrophages (AM) in mice, non-human primates and humans and identify an age-associated increase in a CD11c^+^CD11b^+^ AM population and in mice, demonstrate increased CCR2-dependent recruitment of monocyte-derived AMs and an association between Fas/FasL signaling, inflammation, and altered AM turnover in the aged lung.

## Introduction

By 2030, one in six people globally will be 60 or older, totaling 1.4 billion. By 2050, this group will double to 2.1 billion, and the number of individuals aged 80 and older will triple^1^. Old individuals are more susceptible to severe illnesses and increased mortality due to various diseases^2–5^. Aging is associated with a decline in immune function, a phenomenon known as immunosenescence, which has been studied primarily within the adaptive immune system^6,7^. In parallel, “inflammaging,” or chronic inflammation linked to innate immune system dysfunction with age, is less well understood, especially in the lung alveoli^2,8,9^. Aging lungs are influenced by various biological pathways due to environmental exposures, leading to oxidative stress, inflammation, telomere shortening, DNA damage, mitochondrial dysfunction, epigenetic instability, immune dysregulation, and impaired proteostasis^10^. Oxidative stress and mitochondrial dysfunction in old age, even in the absence of disease, collectively contribute to an inflammatory alveolar environment significantly affecting the phenotype and recruitment of alveolar macrophages (AMs). AMs, the prime immune cells to interact with airborne pathogens and inhaled particulates, are essential for lung health. Despite this knowledge, little is known about the characteristics of AMs present in the lung in healthy old age.

AM development and function are influenced by signals from the lung microenvironment. Shortly after birth, fetal monocytes quickly transform into a stable, self-renewing population called tissue-resident AMs (TRAMs). In mice, TRAMs persist for extended periods without the need for input from bone marrow-derived monocytes^11–13^. TRAMs are located within epithelial surface lining fluid, where they attach to the lung epithelium via integrins and play a crucial role in sampling, responding to, and eliminating pathogens and particulates in the alveoli^14,15^. With TRAM depletion or inflammatory conditions, monocytes are recruited to the alveoli, where they undergo differentiation into monocyte-derived alveolar macrophages (MoAMs) through a progressive reshaping of their epigenome and transcriptome^16–18^. We recently identified distinct AM subpopulations in healthy old mice that are significantly increased relative to young mice^2^. AMs in young mice consist mainly of CD11c^+^CD11b^-^ TRAMs^2,19^, whereas in old mice, there are marked increases in subset populations that are CD11c^+^CD11b^+^ and CD11c^−^CD11b^+^. The importance of these increased subpopulations relates to their specialized inflammatory profile and inability to control *Mycobacterium tuberculosis* growth, suggesting a potential sentinel population for tuberculosis susceptibility in old age^2^. The appearance of these AM subpopulations in old age results from 3 potential mechanisms: i) cell-autonomous changes, ii) alterations in the lung microenvironment, and/or iii) the partial replacement of TRAMs with MoAMs due to repeated insults over the lifespan^20^.

In this work, we investigated how healthy aging shapes AM heterogeneity in mice, humans, and non-human primates using complementary phenotypic, transcriptional, and functional approaches. We identify similar age-associated changes in AM populations across species, including increased CD11c^+^CD11b^+^ and CD11c^−^CD11b^+^ populations and defined distinct transcriptional profiles of AM subpopulations. Mechanistic studies in mice demonstrate that the old age alveolar environment contributes to AM heterogeneity through two complementary processes: phenotypic acquisition of an increased CD11c^+^CD11b^+^ state by resident AMs and recruitment of monocyte-derived AMs. Adoptive transfer and fate-mapping approaches support the monocytic origin of recruited AMs and identify CCR2 as an important mediator of their recruitment into the aged alveolar space. These recruited AMs exhibit increased turnover associated with Fas/FasL signaling. Together, our findings reveal how the old age alveolar microenvironment shapes AM phenotype, recruitment, and turnover, providing insight into the mechanisms underlying altered pulmonary immune homeostasis during healthy aging.

## Results

### Characterization of old/elderly AMs from mice (mAMs), humans (hAMs) and non-human primates (NHPs; baboons: bAMs and rhesus macaque: rAMs): increased CD11c^+^CD11b^+^ subpopulations

**Fig. 1:**
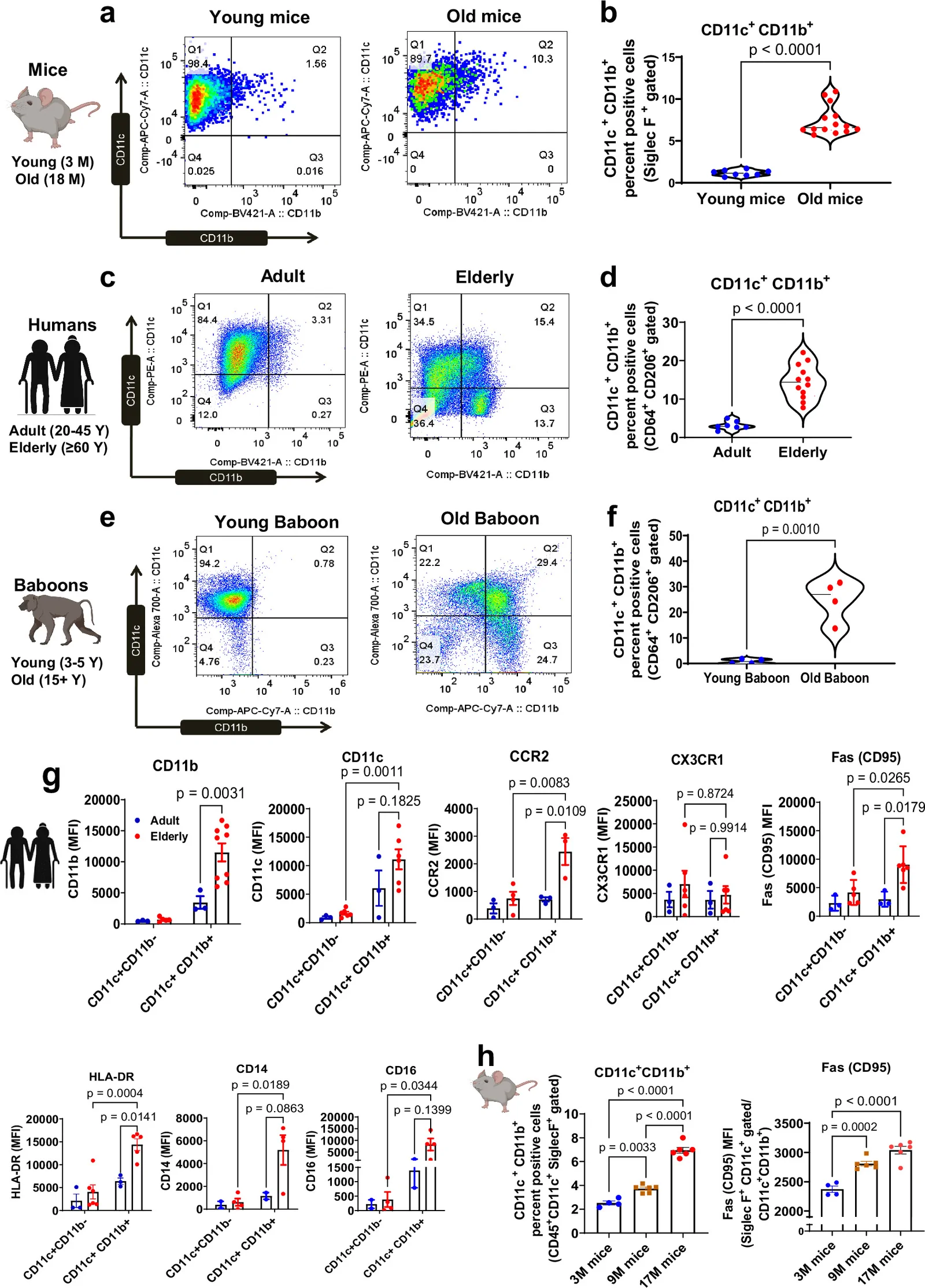
Characterization of AM subpopulations in old mice (mAMs), elderly humans (hAMs), and old non-human primates (baboons, bAMs). **a**, **b** Mouse AMs (mAMs) were isolated from young (3-month-old) and old (18-month-old) mice using bronchoalveolar lavage (BAL). The cells were then stained with fluorochrome-labeled antibodies and analyzed by flow cytometry, specifically gating on the CD45^+^SiglecF^+^CD11c^+^CD11b^+^ AM subpopulations. **c**, **d** Human AMs (hAMs) were isolated by BAL from healthy adult volunteers aged 20–45 years and elderly individuals (≥60 years). The flow-labeled cells were analyzed by gating on the CD45^+^CD64^+^CD11c^+^CD11b^+^ AM subpopulations. **e**, **f** Baboon AMs (bAMs) were isolated by BAL from healthy adult baboons (3-5 years old) and elderly baboons (≥15 years). The flow-labeled cells were also analyzed by gating on the CD45^+^CD64^+^CD206^+^CD163^+^CD11c^+^CD11b^+^ AM subpopulations. **b**, **d**, **f** Violin plot illustrates the percentage of CD11c^+^CD11b^+^ AM subpopulations from hAMs (*N* = 7 adult and 12 elderly human donors) and mAMs (*N* = 8 young and 14 old mice, *N* = 4 young and 4 old baboons). Each dot indicates individual donors, and a two-sided Student’s t-test was used. In elderly individuals, both hAMs and bAMs exhibit a dominant CD11c^+^CD11b^−^ subpopulation, alongside two smaller subpopulations: CD11c^+^CD11b^+^ and CD11c^−^CD11b^+^. mAMs primarily show a dominant CD11c^+^CD11b^-^ subpopulation with an increase in the smaller CD11c^+^CD11b^+^ subpopulation. **g** Flow cytometry analysis of CD11c^+^CD11b^−^ and CD11c^+^CD11b^+^ hAM subpopulations revealed the expression of several cell surface receptors, including CD11b, CD11c, CCR2, Fas (CD95), HLA-DR, CD14, and CD16. Each dot represents an individual human donor with *N* = 3 adults and *N* = 9 elderly donors. Statistical significance was determined using a two-way ANOVA followed by Tukey’s multiple comparisons test (two-sided). **h** Kinetic analysis of CD11c^+^CD11b^+^ and Fas (CD95) mAM subpopulations was conducted in 3-month, 9-month, and 17-month-old mice, pre-gated on CD45^+^SiglecF^+^CD11c^+^CD11b^+^ cells using flow cytometry. Each dot indicates an individual mouse (*N* = 4 for 3 M mice and *N* = 6 for 9 M and 17 M mice). **b**, **d**, **f**–**h** Data are presented as mean ± SEM and were analyzed using an unpaired, two-sided Student’s t-test for two comparisons and an ordinary two-way ANOVA with Tukey’s multiple comparisons test. See also Figs. S1–9. The mice, human and baboon cartoon schematics were created in BioRender. Pahari, S. (2025) https://BioRender.com/x07c111.

To assess AM subpopulations across the species in mice, humans and NHPs, we performed bronchoalveolar lavage (BAL) to obtain their AMs (Fig. 1). We focused on samples from young and old mice (3 months and 18 months old, respectively), healthy adults versus healthy elderly humans (ages 20–45 and ≥ 60 years old), and young and old baboons/rhesus (3–5 years and over 15 years old). We identified the presence of distinct cell populations of AMs, lymphocytes, neutrophils, and eosinophils in the BAL of old/elderly participants compared to young/adults on cytospun samples. AMs were the most prominent population in both groups (Fig. S1). mAM, hAM and bAM subgroups were distinguished by their respective cell surface receptor expression using flow cytometry: mAMs were CD45^+^SiglecF^+^CD11c^+^, hAMs were CD206^+^CD64^+^CD11c^+^, and bAMs were CD45^+^CD64^+^CD206^+^CD163^+^CD11c^+^^21–25^. To identify CD11c^+^CD11b^+^ AM subpopulations, cells were pre-gated on CD45^+^SiglecF^+^ (mAMs) and CD45^+^CD64^+^ (hAMs, bAMs) populations (Figs. 1 and S2–4). In mAMs, the flow cytometry gating strategy was further validated using CD64^+^ and F4/80^+^, with appropriate fluorescence minus one (FMO) and isotype controls (Fig. S2). The data demonstrate that CD45^+^SiglecF^+^CD11c^+^ cells represent the predominant population of mAMs.

### CD11c^+^CD11b^+^ MoAMs are a distinct macrophage subset in old age

TRAMs were the predominant population of cells that express CD11c cell surface receptors. We previously identified an increased subpopulation of CD11c^+^ CD11b^+^ monocyte-derived AMs (MoAM) and a very small subpopulation of CD11c^-^ CD11b^+^ cells in old mice^2^. CD11c^+^ CD11b^+^ MoAMs exhibit a distinct inflammatory signature based on defined markers and enhanced growth of *Mycobacterium tuberculosis*^2^. Our current results confirm these increased subpopulations in old mice (Figs. 1a, b, S5a, b and S7). Our findings in hAMs/bAMs revealed three distinct markedly increased AM subpopulations in the old/elderly groups relative to young individuals (Fig. 1c–f). The most prominent subpopulation was designated as CD11c^+^ CD11b^-^ TRAMs. The 2 smaller subpopulations were CD11c^+^ CD11b^+^ and CD11c^-^ CD11b^+^. Notably, the presence of these two latter subpopulations increased more than tenfold in old baboons and elderly humans when compared to their younger counterparts (Fig. 1c–f). Further analysis indicated that the expression levels of CD11b, CD11c, CCR2, Fas, HLA-DR, CD14, and CD16 were elevated in the CD11c^+^ CD11b^+^ elderly hAM subpopulation (Fig. 1g). There was no significant change in CX3CR1 expression in the CD11c^+^ CD11b^+^ subpopulation of elderly hAMs (Fig. 1g). CD14^+^ CD16^+^ cells are recognized as inflammatory monocytes^26^. We hypothesize that these inflammatory monocytes are recruited to the lungs and in situ differentiated into the CD11c^+^ CD11b^+^ MoAM subpopulation in elderly individuals. Flow cytometry analysis of bAMs revealed that CD11b, CD86, HLA-DR, CD206, CD163, CD64, and CD36 were upregulated in old age when compared to bAMs from young baboons (Fig. S4). Like hAMs, the chemokine receptor CCR2, but not CX3CR1, was upregulated in old age bAMs when compared to young bAMs (Figs. S4 and S8). Further analysis indicated that the expression levels of CD64, CD11c, CD11b, HLA-DR, CD163, and CCR2 were elevated in the CD11c^+^ CD11b^+^ elderly bAM subpopulation (Fig. S8). We also examined the expression levels of AM surface proteins in rhesus macaques. Confocal microscopy showed increased expression of CD163, CD206, CD11b and CD11c in old age rAMs (Fig. S9). Our previous research by Lafuse et al. found that CD11c^+^ CD11b^+^ mAMs from old mice exhibit elevated levels of cell surface receptors Ly6C and CD115, consistent with a monocytic origin^2^. We extended this analysis in mice to examine at what age changes in cell surface receptor expression occur. As age progresses, we observed a gradual increase in CD11c^+^CD11b^+^ recruited AMs with Fas (CD95) expression (Figs. 1h, S5c and S6). Overall, these findings demonstrate significant changes in AM surface receptor expression in healthy old age. The finding of increased subpopulations is consistent across mice, humans and NHPs. Additionally, the findings suggest that the freshly recruited AM subpopulations in old age differentiate from blood monocytes and that the chemokine CCR2 plays an important role in this recruitment.

### Distinct transcriptional profiles of mAM subpopulations in old mice

**Fig. 2:**
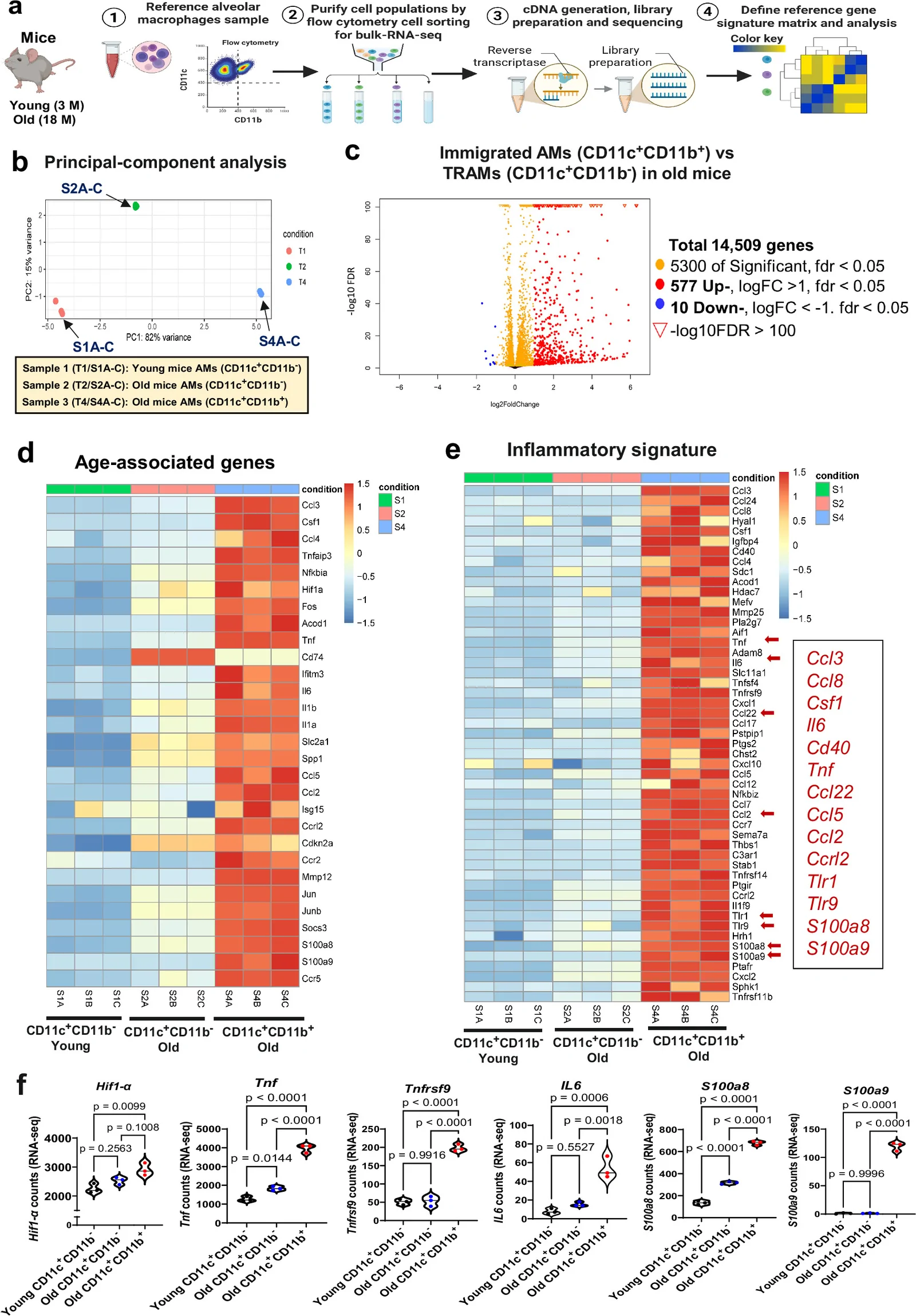
Identification of transcriptional signatures in flow cytometry-sorted young and old mAM subpopulations. The figure presents a transcriptomic analysis using bulk RNA sequencing (RNA-seq) on three flow cytometry-sorted populations: TRAMs from young mice (CD11c^+^ CD11b^-^), TRAMs from old mice (CD11c^+^ CD11b^-^), and immigrated AMs from old mice (CD11c^+^ CD11b^+^). **a** The schematic diagram illustrates the procedure for bulk RNA sequencing. The schematic diagram was created in BioRender. Pahari, S. (2026) https://BioRender.com/x726985. **b** Principal component analysis (PCA) of the transcriptomes from the three different subpopulations of mAMs in young and old mice shows distinct transcriptional signatures and a high degree of similarity among biological replicates within each group (*N* = 10 mice per group, with 3 flow-sorted experiments). **c** The volcano plot displays the false discovery rate (FDR) in relation to the magnitude of gene expression changes across a total of 14,509 genes. The analysis indicates that 577 out of 5300 genes (FDR < 0.05) were significantly upregulated (red), with a log₂ fold change (logFC) > 1, and 10 genes were downregulated (blue) in the CD11c^+^CD11b^+^ AM subpopulations, with a log₂ fold change (logFC) < −1. **d**, **e** The heatmaps show relative expression levels for senescence- and age-associated gene sets and inflammatory signature genes in AM subpopulations from old mice (resident CD11c^+^CD11b^−^ and immigrated CD11c^+^CD11b^+^) and resident CD11C^+^CD11b^−^ from young mice. The red arrow highlights certain inflammatory genes that are highly upregulated in the CD11c^+^CD11b^+^ mAM subpopulations from old mice^,^ indicating specialized signature genes (in red) compared to CD11c^+^CD11b^−^ AMs from either young or old mice. Blue represents low expression, while red represents higher expression. **f** Inflammatory gene signatures (*Hif1α, Tnf, Tnfrsf9, Il6, S1008, S1009*) were identified through RNA-seq analysis, as shown by a violin plot. Data represent 3 independent experiments (*N* = 10 mice per group, with 3 flow-sorted experiments) and were analyzed using an ordinary one-way ANOVA with Sidak’s multiple comparisons test (two-sided). See also Figs. S10 and 11.

To further characterize the AM subpopulations in mice, we conducted a transcriptomic analysis using bulk-RNA sequencing (RNA-seq) on four flow cytometry-sorted populations: TRAMs in young mice (CD11c^+^CD11b^-^), TRAMs in old mice (CD11c^+^CD11b^-^), the increased subpopulation of CD11c^-^CD11b^+^ mAMs in old mice, and the increased subpopulation of CD11c^+^CD11b^+^ mAMs in old mice (Figs. 2a and S10a–c). CD11c^-^CD11b^+^ mAMs in old mice were present in very low numbers and exhibited a transcriptional profile like that of the CD11c^+^CD11b^+^ populations. Thus, we did not include them in our further analyses. CD11c^+^CD11b^+^ mAMs are rare in young mice, comprising less than 1–2% of the total AM population. As a result, they were excluded because it was not feasible to isolate sufficient cells by flow cytometric sorting for downstream transcriptomic analysis. Thus, our primary focus was on comparing three subpopulations of mAMs (CD11c^+^ CD11b^-^ mAMs in young and old mice (TRAMs) and CD11c^+^CD11b^+^ mAMs in old mice).

Principal component analysis (PCA) indicated a high degree of similarity among biological replicates within each group (Fig. 2b). Volcano plots illustrated the false discovery rate (FDR) in relation to the magnitude of change in gene expression across a total of 14,509 genes. 577 out of 5300 genes (FDR <0.05) were significantly upregulated, with a log2 fold change (logFC) > 1, in the CD11c^+^CD11b^+^ mAMs (Supplementary Data 1a). Moreover, 10 genes were downregulated (logFC < −1) when compared to TRAMs (CD11c^+^CD11b^−^) in old mice (Fig. 2c and Supplementary Data 1b). We next focused on CD11c^+^CD11b^+^ mAMs for their relationship to curated age-associated inflammatory senescence-related gene signatures from the literature^27–29^. We identified 29 genes, e.g., *Il1b, Il1a, Tnf, Il6, Ccl2, Ccr2, Ccl3, Ccl4, Ccrl2, Ccr5, Ccl5, Isg15, Ifitm3, Slc2a1, Hif1a, Acod1, S100a9, S100a8, Csf1, Cd74, Nfkbia, Cdkn2a, Socs3, Fos, Jun, Mmp12, Spp1, Tnfaip3,* and *Junb* that were significantly upregulated in CD11c^+^CD11b^+^ mAMs from old mice compared to CD11c^+^CD11b^-^ mAMs in old and young mice (Fig. 2d). Consistent with this, inflammatory pathway genes, including *Ccl3, Ccl8, Csf1, Cd40, Tnf, Ccl22, Ccl2, Ccrl2, Ccr2, Ccl5, Tlr1, Tlr9, S100a8, and S100a9*, were also markedly enriched in CD11c^+^CD11b^+^ mAMs in old mice compared to CD11c^+^CD11b^-^ mAMs in old and young mice (Fig. 2e). TNF signaling-related genes, other immune response-related genes, including those involved in positive regulation of the ERK1/2 pathway, cell migration and chemotaxis were also upregulated in the old mice CD11c^+^CD11b^+^ mAM populations (Fig. S11a–d). Notably, *Hif1a* was upregulated in CD11c^+^CD11b^+^ mAMs, which suggests induction of glycolysis in this AM subpopulation. Inflammatory genes were observed in CD11c^+^CD11b^+^ mAMs (Fig. 2f), highlighting a distinct inflammatory profile consistent with a previous gene expression study^2^. Quantitative PCR analysis in hAMs confirmed significantly higher levels of inflammatory, immunoregulatory, and cell death markers in elderly compared to adult hAMs (Fig. S12).

We also compared TRAM populations in young and old mice, where 311 out of 4,796 genes (FDR < 0.05) were significantly upregulated (logFC > 1) in old TRAMs (Supplementary Data 2a, b). Moreover, of those, 36 genes were downregulated (logFC < −1, FDR < 0.05) in old mice when compared to TRAMs in young mice (Fig. S10b**)**. Interestingly, we also observed that age-associated inflammatory and senescence-related gene signatures were upregulated in TRAMs from old mice compared to young mice (Fig. S10c), indicating that the old age lung environment also impacts resident AM populations.

### Metabolic and oxidative changes of AMs in old age mice

RNAseq results suggested major changes in metabolism and oxidative stress in AMs in old age. HIF-1α regulates glucose metabolism to promote anaerobic glycolysis and conserve ATP^30^. Healthy adult AMs are classically driven primarily through oxidative phosphorylation (OXPHOS)^31^, but there is evidence for a metabolic shift in AMs from OXPHOS to glycolysis in old age^32^. We posited that increased HIF-1α expression in old AMs will lead to increased glycolysis and decreased OXPHOS, and that the complex inflammatory and oxidative alveolar environment^20^ in old age changes AM phenotype.

We measured glycolysis and OXPHOS by quantifying the ECAR and OCR in old versus young total mAMs. Our findings revealed a significant increase in both the basal and maximal respiratory capacity and ATP production, and spare respiratory capacity, along with a moderate increase in proton leak in mAMs from old mice compared to those from young mice (Fig. S13a–f). Additionally, glycolysis (ECAR) in the mAMs from old mice was significantly higher than that in young mice (Fig. S13g–j**)**. Previous data indicated a significant increase in TLR2 expression (both RNA and protein levels) in AMs from old mice, with higher levels observed specifically in the CD11c^+^ CD11b^+^ AM subset^2,33^. Similarly, we found that TLR2 expression was elevated in hAMs from elderly individuals (Fig. S12d). To investigate the TLR2 response in mAMs from old mice, we stimulated these cells in vitro with the TLR2 agonist Pam_3_CSK_4_. Following stimulation, we observed elevated RNA expression levels of aerobic metabolism-related genes, including *Hif1α, Cox2, CD74, Mif, Glut1, Hk2, Pfkb3, Gls1, Slc25a*, and *Acly* (Fig. S14). In old mAMs, there was an increase in both OXPHOS (OCR) and glycolysis (ECAR), attributed to heightened glucose utilization, mitochondrial respiration, and ATP production. However, this pattern was not observed in hAMs, where the glycolysis-related gene (*LDHA*) was elevated, and the OXPHOS-related gene (*NDUFAF6*) decreased in old age (Fig. S15). One possible explanation for these differences is that the metabolic pathways (OXPHOS versus glycolysis) in mice operate independently. Another possibility is that old mAMs have high glucose availability to drive glycolysis, while simultaneously having low ATP levels, which activates the citric acid cycle to produce significantly more ATP through OXPHOS^31^. Finally, we observed a significant increase in mitochondrial ROS in old mAMs as compared to young mAMs (Fig. S16a, b).

### The inflammatory alveolar environment in old age shapes the AM phenotype

AMs within the alveolar environment are bathed in an aqueous alveolar hypophase, recycle surfactant, and contact Type I and Type II epithelial cells^34^. Delicate alveolar air sacs enable gas exchange. Such an environment requires a highly regulated inflammatory response to enable normal lung function. Recent studies in old age have shown significant changes to protein production and function in the alveolar hypophase, e.g., pro-inflammatory cytokines, altered surfactant proteins and lipids, and modifications to complement components. These changes contribute to an environment with heightened oxidative stress, leading to an enhanced lung inflammatory state^3,35^. We next explored the role of this altered alveolar environment in old age in the phenotype and recruitment of the AM subpopulations.

**Fig. 3:**
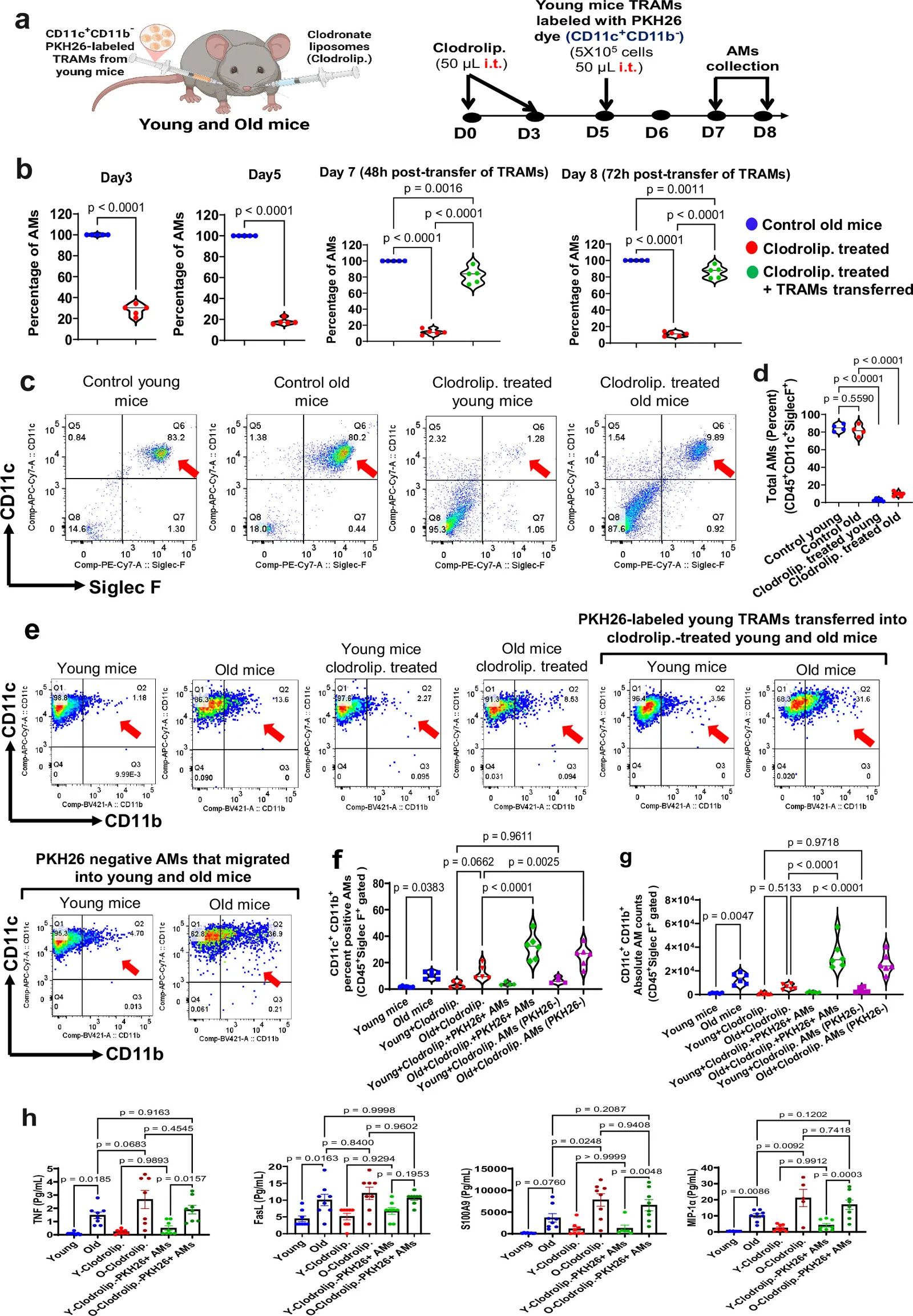
Depletion of resident mAMs to assess the alveolar environment in old age. To characterize the Mo-AM subpopulation and investigate the role of the alveolar environment in old mice, two doses of clodronate liposomes (clodrolip.) were administered intratracheally (i.t.) on Day 0 and Day 3. This treatment effectively depleted resident AMs from the alveolar compartments of both young and old mice. On Day 5, CD11c^+^ CD11b^−^ bronchoalveolar lavage (BAL) isolated resident AMs (donor cells) from young mice were adoptively transferred, i.t. route (using 5 × 10^5^ cells) into both young and old recipient mice. **a** A schematic representation outlines the experimental approaches used. The schematic diagram was created in BioRender. Pahari, S. (2026) https://BioRender.com/b41u275. **b** Cytospin staining analysis presented in the violin plot shows the percentage of AM depletion on Days 3, 5, 7, and 8. Compared with control old mice (blue dots), set as 100%, approximately 10–15% of cells remained detectable after clodronate liposome (clodrolip) treatment (red dots), likely due to immigration of cells into the alveolar space. Transferred CD11c^+^CD11b^−^ donor AMs were detected in recipient mice (green dots). Each dot represents an individual mouse with 5 mice/group (*N* = 5). Data were analyzed by two-sided Student’s t-test for two comparisons and ordinary one-way ANOVA with Sidak’s multiple comparisons test (Two-sided). **c** Flow cytometry dot plot analysis on gated population, shows the percentage of CD11c^+^ Siglec F^+^ AMs depleted (Q6 gate: 83.2 and 80.2% to 1.28 and 9.89%) after clodrolip. treatment on day 5 in young and old mice. **d** The violin plot shows the percentage of AM depleted in the clodrolip-treated group. Data are pooled from 4 control young and 4 control old mice, and from 8 experimental mice per group (*N* = 8), and analyzed using an ordinary one-way ANOVA with Sidak’s multiple comparisons test (two-sided). **e** Flow cytometry dot plot analysis (Q2 gate) reveals the presence of CD11c^+^CD11b^+^ MoAMs (Q2 gate: 1.18 and 13.6%) in young and old mice, while the percentage of MoAMs in the clodrolip-treated group was 2.27 and 8.53% (Q2 gate). Following the adoptive transfer of PKH26-labeled BAL isolated TRAMs (CD11c^+^CD11b^-^) from young mice, the phenotype was reprogrammed to CD11c^+^CD11b^+^ MoAMs (Q2 gate: 3.56 and 31.6%) in young/old mice. The percentage of PKH26-negative MoAMs migrated into young/old mice was (Q2 gate: 4.70 and 36.9%). The red arrow indicates the population of interest. **f**, **g** Violin plot shows the percentage of CD11c^+^CD11b^+^ MoAMs and absolute AM counts in CD45^+^SiglecF gated populations. Data pooled from 6 mice/group (*N* = 6) and analyzed using an ordinary one-way ANOVA (two-sided) with Sidak’s multiple comparisons test. **h** Elevated levels of inflammatory mediators, e.g., TNFα, FasL, S100A9, and MIP-1α proteins, were detected in the BAL fluid of young and old mice, as well as in clodrolip-treated and recipients of PKH26-labeled TRAMs from young mice. Each dot indicates an individual mouse/group (*N* = 8) and analyzed using an ordinary one-way ANOVA (two-sided) with Sidak’s multiple comparisons test. Data represent 3 independent experiments and are expressed as mean ± SEM, analyzed using an ordinary one-way ANOVA with Sidak’s multiple comparisons test. See also Figs. S7 and S12–17.

Based on the transcriptome analyses of TRAMs from young and old mice, we addressed the question of whether the old age alveolar environment impacts the phenotype of TRAMs. We depleted TRAMs from both young and old mice by administering two doses of clodronate liposomes (clodrolip.) intratracheally (i.t.) on Day 0 and Day 3 per standard approach (Fig. 3a, b)^36,37^. We achieved ˃ 85–90% AM depletion by Day 5–8 by Cytospin (Fig. 3b and S17) and flow cytometry (Fig. 3c-d). On Day 5, we introduced PKH26 lipophilic dye-labeled BAL-isolated TRAMs (CD11c^+^ CD11b^−^) from young mice (5 × 10^5^ cells) i.t. into both young and old recipient mice (Fig. 3a). We then performed BAL 48 h later. This approach allowed us to specifically assess the impact of the old age alveolar environment on the phenotype of PKH26-labeled transferred TRAMs. Simultaneously, we identified and characterized PKH26^−^ AMs. A significant percentage (30–40%) of the transferred PKH26^+^ TRAMs from young mice phenotypically reprogrammed to a CD11c^+^CD11b^+^ mAMs in old mice at this time point (Figs. 3e–g and S7). This percentage was also seen in the PKH26^−^ AMs. Thus, we conclude that TRAMs are reprogrammed to a CD11c^+^CD11b^+^ state associated with the inflammatory alveolar milieu in old age^20,35^. In concert with this, we observed elevated levels of soluble TNF, FasL (CD178), S100A9, and MIP-1α proteins in the isolated BAL fluid from old mice compared to young mice, which are maintained after clodrolip. treatment and even after PKH26-labeled young mice AM transfer (Fig. 3h).

### The inflammatory alveolar environment in old age increases the recruitment of MoAMs from the periphery

**Fig. 4:**
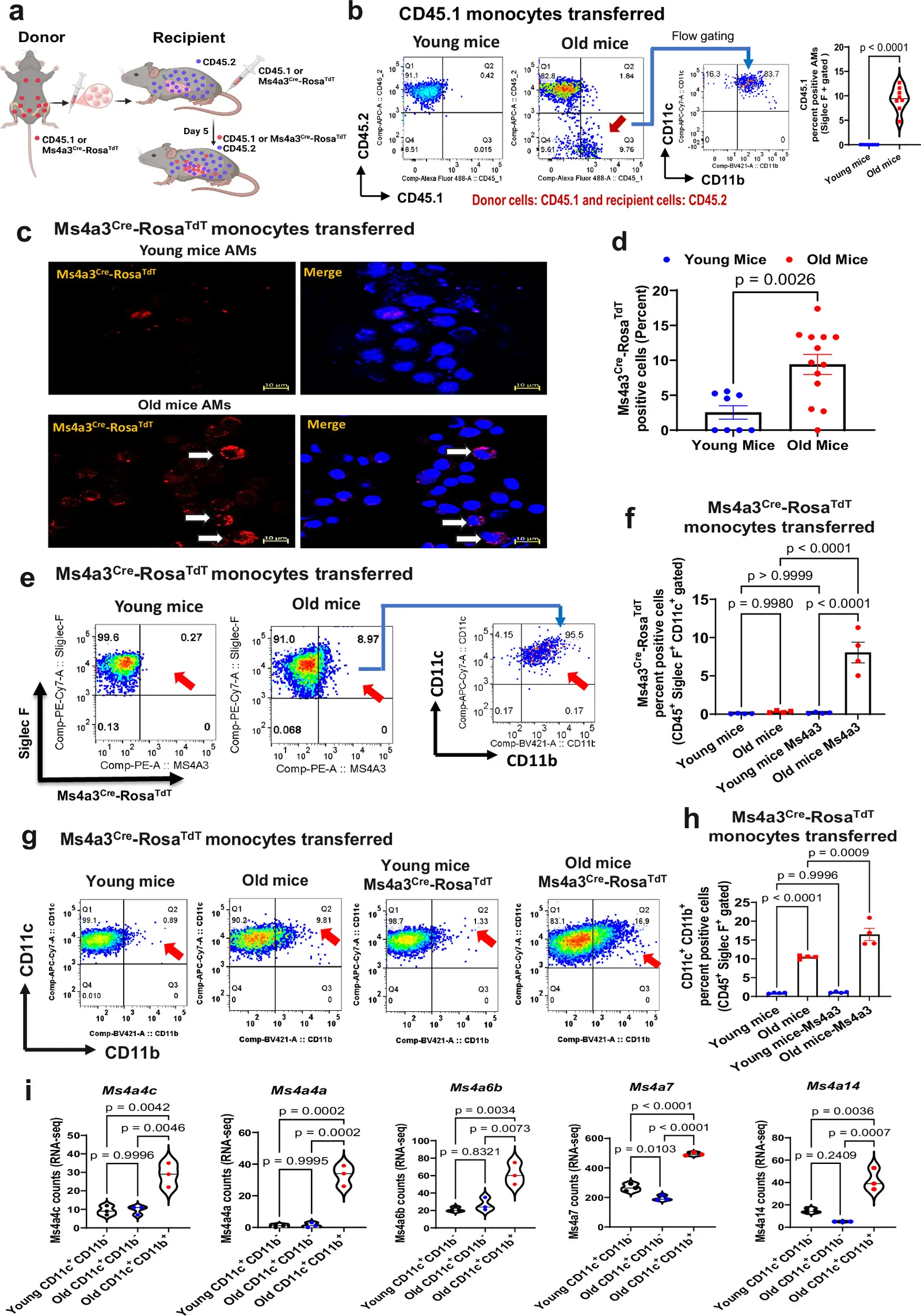
Adoptive transfer of monocytes from young mice into recipient young and old mice. CD45.1 or Ms4a3^Cre^-Rosta^TdT^ monocytes (donor cells) were isolated by magnetic sorting (Ly6c^+^ negative selection kit) from the bone marrow of young mice and transferred into CD45.2 (recipient) young and old mice via tail-vein injection (i.v.). After 5 days, BAL was performed. Recovered AMs were labeled for flow cytometry analysis. **a** A schematic representation of the experimental approaches is shown. Created in BioRender. Pahari, S. (2026) https://BioRender.com/p76z776. **b** Flow cytometry dot plot data indicate that CD45.2-positive cells are consistent among young and old mice AMs. CD45.1 cells were successfully recruited into the alveolar space of old mice, as indicated by the red arrow highlighting the recruited CD45.1-positive cells (Q3 gate: 9.76%). The violin plot shows the percentage of CD45.1^+^ cells in SiglecF-gated populations. Data were pooled from *N* = 8 mice/group, and a two-sided Student’s t-test was used. **c**, **d** Ms4a3^Cre^-Rosa^TdT^ macrophages (in red) were identified using confocal microscopy. The white arrow points to Ms4a3^Cre^-Rosa^TdT^ positive cells. Scale bar represents 10 µm at 63x magnification. In (**d**), the quantification of Ms4a3^Cre^-Rosa^TdT^ positive cells (as a percentage) was performed from over 100 macrophages (DAPI positive in blue) per microscopic field. Data were analyzed from 8 young and 13 old mice AMs, and a two-sided Student’s t-test was used. **e** The dot plot from flow cytometry illustrates the presence of Ms4a3^Cre^-Rosa^TdT^ AMs detected (8.97%) in old mice within the CD45^+^SiglecF^+^CD11c^+^ populations. Further flow gating of the 8.97% population reveals that Ms4a3^Cre^-Rosa^TdT^ positive AMs are also part of the CD11c^+^CD11b^+^ AM population (95.5%). The red arrow indicates the population of interest. **f** A bar graph summarizes cumulative data of Ms4a3^Cre^-Rosa^TdT^ macrophages from 4 mice per group (*N* = 4). Statistical significance was determined using a one-way ANOVA (two-sided) with Sidak’s multiple comparisons test. **g** Another contour plot shows that old mice exhibited an increased recruitment of the CD11c^+^ CD11b^+^ AM subpopulations. The red arrow indicates the population of interest. **h** A bar graph demonstrates cumulative data for the CD11c^+^ CD11b^+^ AM subpopulations from 4 mice per group (*N* = 4). Statistical significance was determined using a one-way ANOVA (two-sided) with Sidak’s multiple comparisons test. **i** RNA sequencing analysis indicates that several Ms4a-related gene sets (*Ms4a4c, Ms4a4a, Ms4a6b, Ms4a7, Ms4a14*) were upregulated in CD11c^+^CD11b^+^ monocyte-derived alveolar macrophages (MoAMs) from old mice compared to the resident AMs (CD11c^+^CD11b^−^) in both young and old mice. Data were analyzed using an ordinary one-way ANOVA (two-sided) with Sidak’s multiple comparisons test. Each dot indicates 3 flow-sorted experiments with *N* = 10 mice per group. Data are representative of 3 independent experiments and are expressed as the mean ± SEM, with an Unpaired Student’s t-test for two comparisons and an ordinary one-way ANOVA (two-sided) with Sidak’s multiple comparisons test. See also Fig. S19.

We next employed adoptive transfer using CD45.1 congenic mice (Fig. 4a). CD45.1 bone marrow monocytes were isolated from young mice and transferred to young and old CD45.2 mice via tail vein injection. After 5 days, BAL was performed, and the recovered CD45.1 mAMs were labeled for flow cytometry analysis, which revealed that they effectively migrated into the alveolar spaces of old mice, but much less so in young mice (Figs. 4b and S18).

Finally, we employed the Ms4a3^Cre^-Rosa^TdT^ fate mapper mouse model to assess the origin of recruited MoAMs (Fig. 4a). The Ms4a3 gene is specifically expressed in monocyte-committed progenitors^38^, and in this model, the bone marrow cells are labeled with an irreversible tdTomato red fluorescent marker, which identifies macrophage progenitor cells^38^. We adoptively transferred BM monocytes from the Ms4a3^Cre^-Rosa^TdT^ mice into both young and old mice via tail vein injection (Fig. 4a). In young mice, we observed the presence of Ms4a3^Cre^-Rosa^TdT^ monocytes in the peripheral circulation, but these cells did not reach the lungs. In contrast, old mice exhibited fewer monocytes in circulation, suggesting that these cells migrated to the lungs and other organs (Fig. S19). Confocal microscopy, flow cytometry, and histopathology analyses revealed a higher recruitment of Ms4a3-derived MoAMs in old mice into the alveolar space (Figs. 4c–f and S19). Furthermore, the Siglec F^+^Ms4A3 double-positive AM population (8.97%) corresponded to the CD11b^+^CD11c^+^ mAM (95.5%) subpopulation (Fig. 4e). Additional flow cytometry data corroborated that after the adoptive transfer of Ms4a3^Cre^-Rosa^TdT^ monocytes, old mice showed increased recruitment of both the Ms4a3^Cre^-Rosa^TdT^ and CD11c^+^ CD11b^+^ AM subpopulations (Figs. 4g, h and S20).

RNA sequencing analysis revealed that several Ms4a-related gene sets (*Ms4a4c, Ms4a4a, Ms4a6b, Ms4a7*, and *Ms4a14*)^39^ are upregulated in CD11c^+^CD11b^+^ MoAMs from old mice compared to the TRAMs in both young and old mice (Figs. 2d and 4i). These data correlate with the evidence that the MoAMs in old mice originate from bone marrow monocytes (CD11b^+^, Ly6c^+^, CD11c^−^), which subsequently differentiate into CD11c^+^CD11b^+^ MoAMs within the lung environment. Overall, the findings demonstrate the importance of the altered alveolar environment in old age in recruiting distinct AM subpopulations derived from peripheral monocytes.

### Recruitment of MoAMs in old age is dependent on a CCR2-mediated pathway

**Fig. 5:**
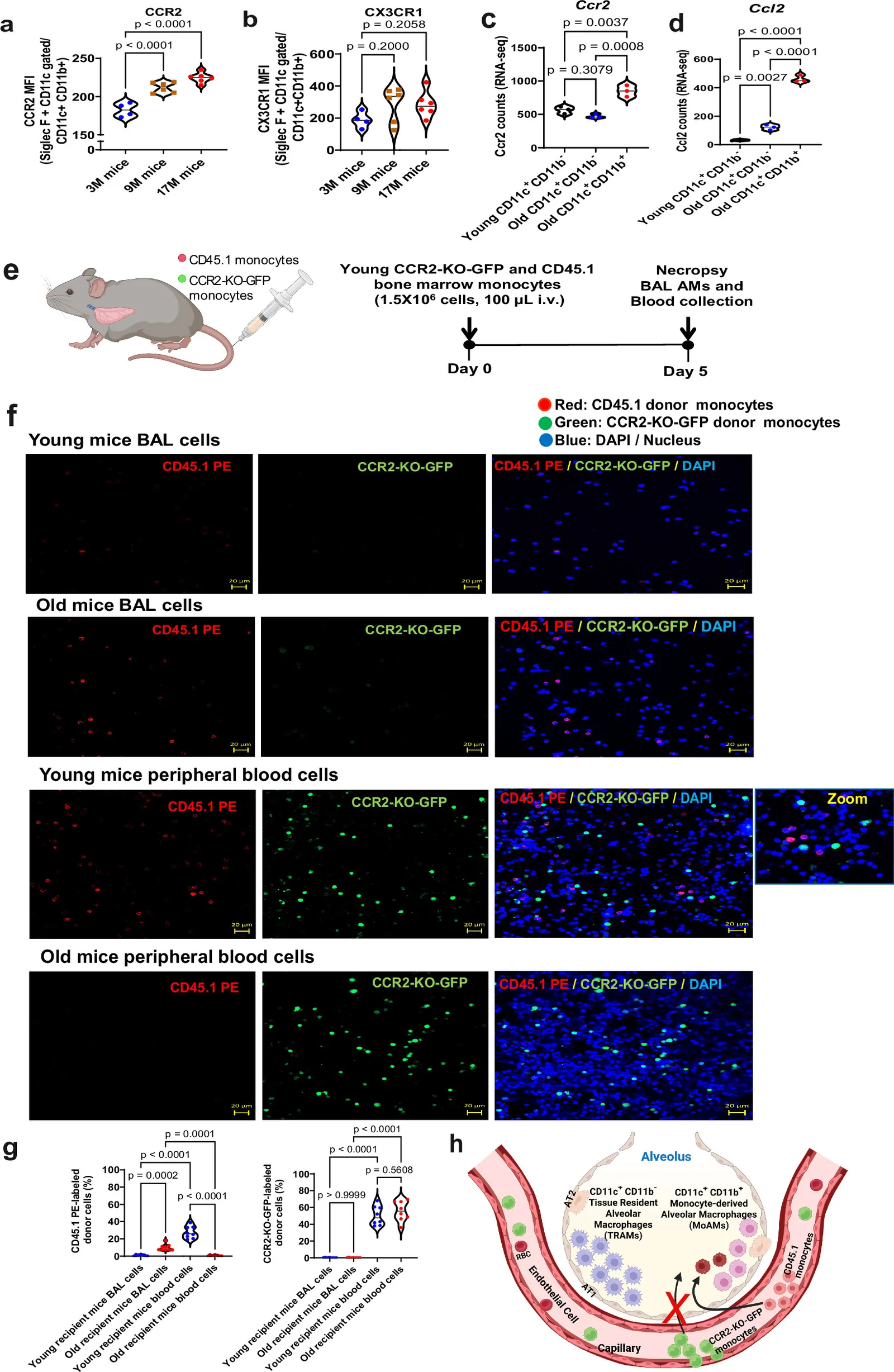
Recruitment of monocyte-derived macrophages (MoAMs) through a CCR2-dependent pathway. MoAM were isolated from mice aged 3 months (3 M), 9 months (9 M), and 17 months (17 M). **a**, **b** Kinetic analysis of CCR2 and CX3CR1 expression in 3 M, 9 M, and 17 M old mice was conducted using flow cytometry, pre-gated on CD45^+^SiglecF^+^CD11c^+^CD11b^+^ cells. Each dot indicates an individual mouse (*N* = 4 for 3 M mice and *N* = 6 for 9 M and 17 M mice) with ordinary one-way ANOVA (two-sided), Sidak’s multiple comparisons test applied. **c**, **d** RNA sequencing (RNA-seq) data showed increased expression of *Ccr2* and *Ccl2* genes in CD11c^+^CD11b^+^ MoAMs from old mice compared to the resident AMs (CD11c^+^) in both young and old mice. Data were analyzed using an ordinary one-way ANOVA (two-sided) with Sidak’s multiple comparisons test. Each dot indicates 3 flow-sorted experiments (*N* = 3) with 10 mice per group. **e** Schematic representation of the experimental approaches used. Competitive adoptive transfer of bone marrow monocytes from both CD45.1 and CCR2 knockout-GFP mice were performed via tail vein injections (i.v.) into both young and old recipient mice. On day 5, BAL was collected from the recipients. Created in BioRender. Pahari, S. (2026) https://BioRender.com/p14c931. **f** Confocal microscopy revealed GFP-positive cells (GFP: green, Nucleus: DAPI-blue) primarily observed in the bloodstream, rather than the alveolar compartment. At the same time, CD45.1^+^ (CD45.1 PE: Red, Nucleus: DAPI-blue) donor cells efficiently populated the alveolar space in old mice but were detected at low frequency in young mice, as assessed by CD45.1 antibody labeling in BAL cells. The majority of CD45.1 cells are found in the bloodstream in young mice. **g** Violin plot summarizes the cumulative data of CD45.1% and CCR2-KO-GFP% from 8 young (3 M) and 8 old (18 M) mice, and analyzed using an ordinary one-way ANOVA (two-sided) with Sidak’s multiple comparisons test. Each dot represents an individual mouse (*N* = 8), with 5–7 microscopic fields (20x) containing more than 100 (BAL cells) to over 500 (blood cells). **h** Pictorial diagram illustrates a significant migration of CD45.1 cells (Red) in old mice into the alveolar space and a migration defect in CCR2-KO-GFP bone marrow monocytes (green), identifying the critical role of CCR2 in this recruitment process. Created in BioRender. Pahari, S. (2026) https://BioRender.com/o8btp10. The data are representative of 3 independent experiments and expressed as mean ± SEM, with ordinary one-way ANOVA and Sidak’s multiple comparisons test applied.

To investigate the mechanism(s) involved in the recruitment of MoAMs to the lungs in old age, we focused on C–C chemokine receptor type 2 (CCR2), which in adult mice is essential for activating and recruiting monocytes and macrophages to kidney, liver, myocardium and skin injury tissue sites, contributing to tissue inflammation^40,41^. TRAMs are known to lack CCR2 expression^42^. In contrast, our results show that the CD11c^+^ CD11b^+^ MoAMs express CCR2 on their surface in the alveolus, which increases with age (Figs. 5a and S21), a finding also shown on hAMs (Fig. 1g) and bAMs (Figs. S4b and S8b). There was no significant change in surface expression of CX3CR1 with age in CD11c^+^ CD11b^+^ MoAMs, although gene expression increased in hAM (Figs. 1g, S4b, S8b and S12k). Although a trend toward increased CX3CR1 expression was observed, multiple independent experiments confirmed no consistent, significant difference between old and young mice (*N* = 15, *P* = 0.33) (Figs. 5b and S22). RNA-seq data also demonstrated significant upregulation of both *Ccr2* and *Ccl2* genes in CD11c^+^ CD11b^+^ MoAMs (Figs. 2f and 5c, d) and increased CCR2 expression in old hAMs (Fig. S12L). To determine the role of CCR2, we conducted competitive adoptive transfer of BM monocytes from CD45.1 and CCR2 knockout-GFP knockin mice via tail vein injections into both young and old recipient mice (Fig. 5e). CD45.1^+^ donor cells efficiently populated the alveolar space in old mice but much less so in young mice, as assessed by CD45.1 antibody labeling in BAL cells (Fig. 5f, g). In contrast, the majority of the CCR2-KO-GFP bone marrow monocytes remained in the bloodstream (Fig. 5f, g) and did not reach the alveolar space in young or old mice, indicating an essential role of CCR2 in facilitating this process in old age (Fig. 5h).

### Recruited MoAMs are relatively short-lived when compared to resident AMs

**Fig. 6:**
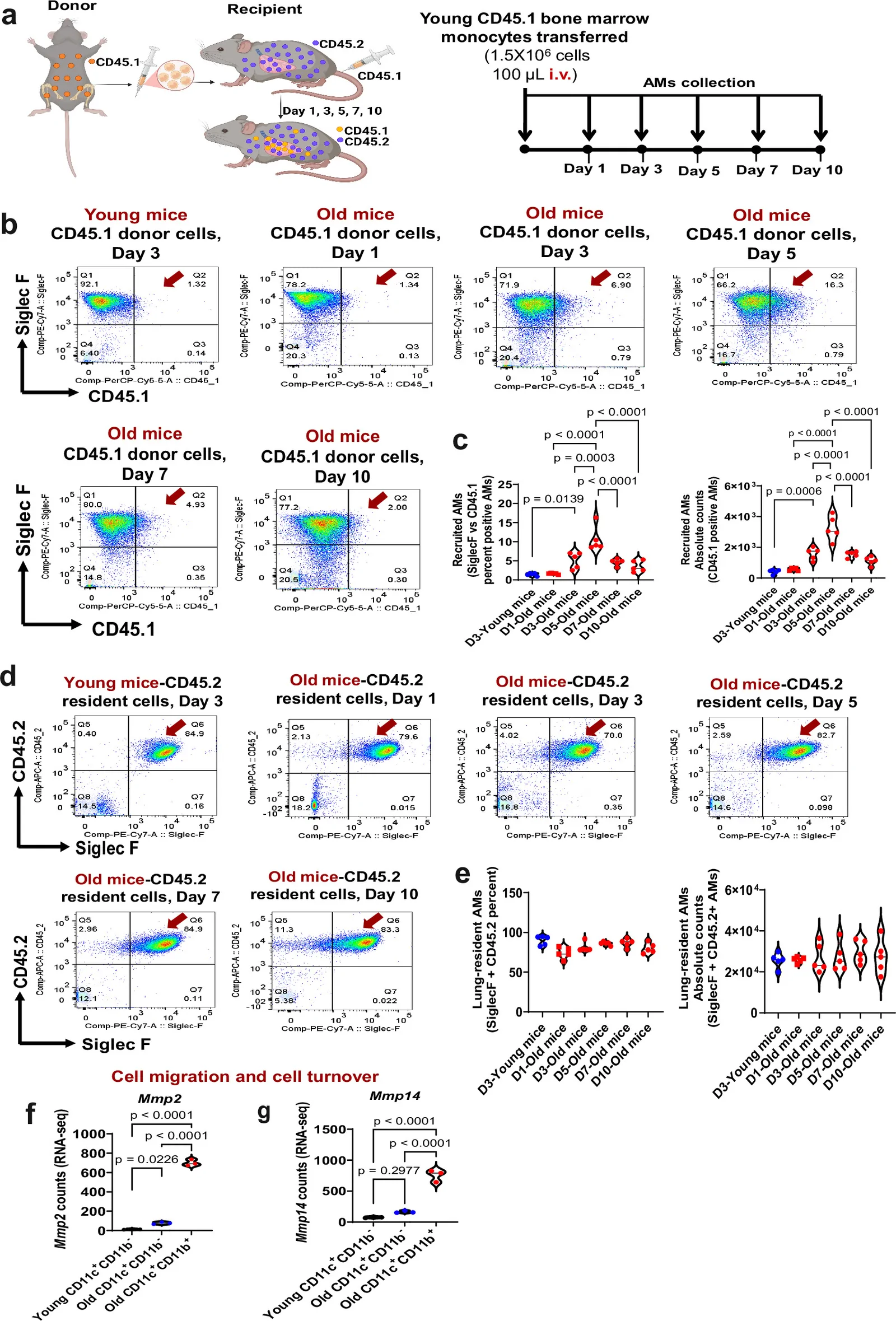
Kinetic adoptive transfer study determines mAM turnover. CD45.1 monocytes (donor cells) were isolated from the bone marrow of young mice and transferred (at 1.5 × 10^6^ cells per mouse) via tail-vein injections (i.v.) into young and old CD45.2 recipient mice. After 1, 3, 5, 7, and 10 days, BAL was performed. Recovered CD45.1 AMs were labeled for flow cytometry analysis. **a** A panel provides a schematic representation of the experimental approaches used. Created in BioRender. Pahari, S. (2026) https://BioRender.com/9dilu6q. **b** Flow cytometry dot plot data indicate that CD45.1 monocytes actively migrated into the alveolar region and differentiated into AMs, with peak differentiation occurring by Day 5. The red arrow highlights the maximum recruitment of CD45.1-positive cells (% SiglecF^+^CD45.1^+^) at Day 5, followed by a gradual reduction in the percentage of recruited AMs over time in old mice. **c** The violin plot presents cumulative data showing the percentage of CD45.1 cells pre-gated on SiglecF^+^CD11c^+^ AMs from five mice per group (*N* = 5). The absolute number of CD45.1^+^ recruited AMs recovered from BAL is shown in the violin plot. Data were analyzed using an ordinary one-way ANOVA (two-sided) with Sidak’s multiple comparisons test. **d** Flow cytometry dot plot illustrates CD45.2 resident AMs pre-gated on SiglecF^+^CD11c^+^ populations. **e** Violin plot demonstrates cumulative data on CD45.2-positive resident AMs (% CD45.2^+^ SiglecF^+^) and absolute number, which remain consistent in both young and old mice. Data are expressed from 5 mice per group. **f**, **g** RNA-seq analysis reveals that the genes *Mmp2* and *Mmp14* are upregulated in CD11c^+^CD11b^+^ MoAMs from old mice compared to the resident AMs (CD11c^+^ CD11b^−^) in both age groups. Each dot indicates an individual experiment (*N* = 3) with 10 mice/group/experiment. Data are representative of 3 independent experiments and are expressed as mean ± SEM, analyzed using ordinary one-way ANOVA (two-sided) with Sidak’s multiple comparisons test.

To investigate the turnover of recruited MoAMs, we adoptively transferred CD45.1 young monocytes into both young and old mice via tail vein injection. At specified time points (Days 1, 3, 5, 7, or 10), we isolated and analyzed the mAM subpopulations in the alveolar space, identified by the markers SiglecF^+^CD45.2 for TRAMs and CD45.1 for recruited cells (Fig. 6a).

**Fig. 7:**
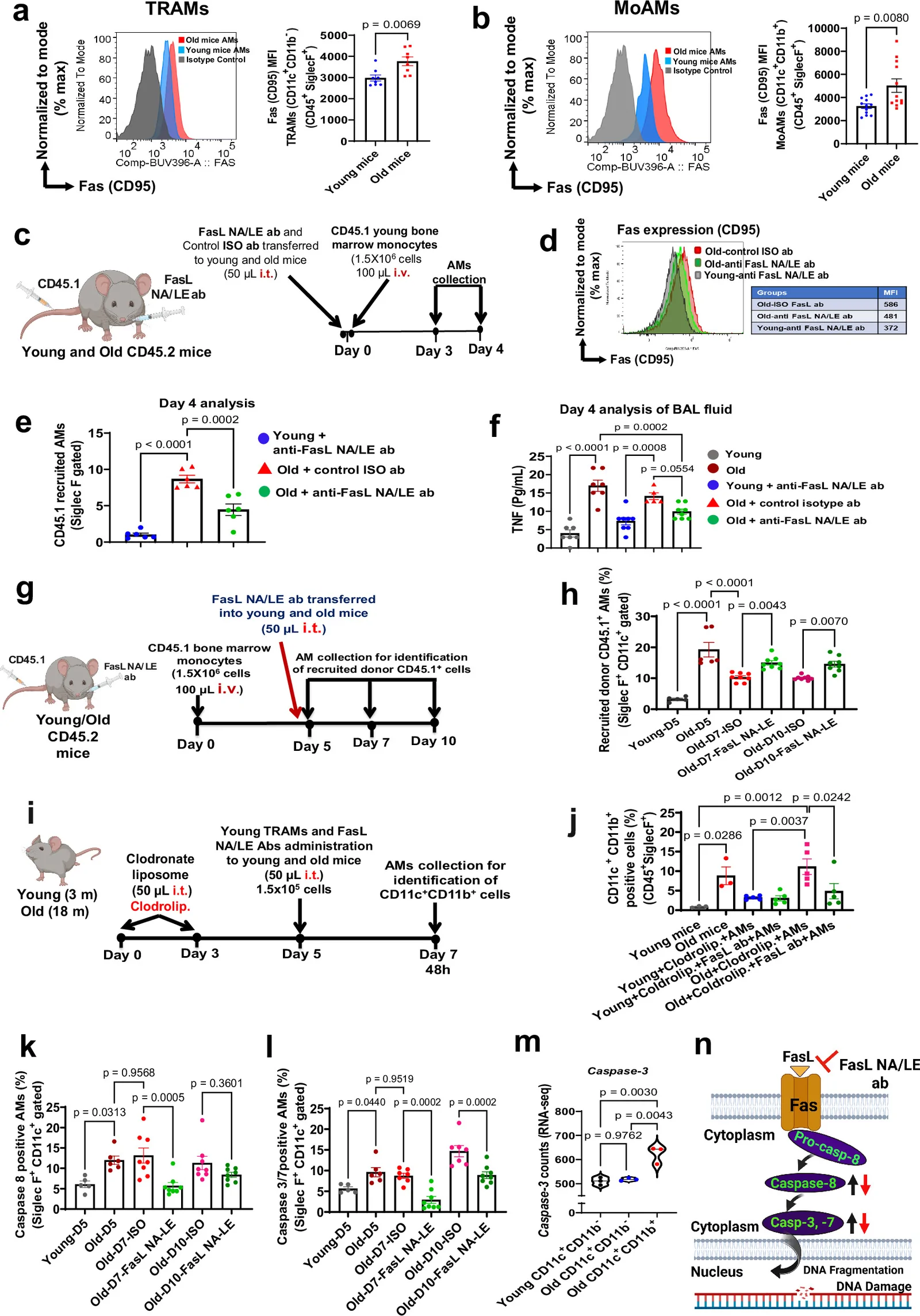
Adoptive transfer of CD45.1 monocytes and treatment with FasL (CD178) NA/LE antibody extends the lifespan of mAMs. **a**, **b** Mouse AMs (mAMs) were isolated through BAL from young (3 months) and old (18 months) mice, then stained with fluorochrome-labeled CD45, CD64, SiglecF, CD11c, CD11b, and Fas (CD95) antibodies. mAM subpopulations were analyzed by flow cytometry, using CD45^+^SiglecF^+^CD11c^+^ gating. The histogram plot represents Fas (CD95) expression in TRAM and MoAM populations in young (blue) and old (red) mice. Bar graph presents cumulative data on Fas (CD95) mean fluorescence intensity (MFI) from 8 mice per group (TRAMs, *N* = 8) and (MoAMs, *N* = 12), expressed as mean ± SEM. Statistical analysis was performed using an unpaired Student’s t-test (two-sided). **c** Schematic representation of the experimental approach is shown. Young and old CD45.2 mice were treated i.t. with FasL neutralizing antibody (CD178, FasL NA/LE Ab) (50 µL, 0.5 mg/mL), or an isotype control antibody. Concurrently, CD45.1 young mice bone marrow monocytes (isolated by magnetic sorting for Ly6c^+^, negative selection) were transferred i.v. AMs were isolated from the BAL of each group on day 4. Cartoon schematics in panel c were created in BioRender. Pahari, S. (2026) https://BioRender.com/q98c015. **d** Flow cytometry analysis histogram plots demonstrate a reduction in Fas (CD95) receptor expression following treatment with FasL Ab and corresponding MFI value (MFI 586 to 481). **e** Adoptive transfer of CD45.1 monocytes, combined with the blockade of FasL by anti-FasL Ab, effectively reduced the recruitment of CD45.1 MoAMs in old mice compared to the control isotype-treated group. Data are expressed as mean ± SEM from 6 mice per group (*N* = 6), analyzed by ordinary one-way ANOVA (two-sided) with Sidak’s multiple comparisons test. **f** TNF levels were measured in young and old mice treated with FasL Ab in the isolated day 4 BAL fluid. Data are expressed as mean ± SEM from 8 mice per group (*N* = 8), analyzed by ordinary one-way ANOVA (two-sided) with Sidak’s multiple comparisons test. **g** CD45.1 young monocytes were transferred to both young and old CD45.2 mice by i.v., with i.t. treatment using FasL Ab on day 5 to assess the survival of already immigrated CD45.1 cells in the alveolus. BAL conducted 2- and 5-day post i.t. Ab administration for identification of recruited donor CD45.1^+^ cells. The experimental approach is depicted. Cartoon schematics in panel g were created in BioRender. Pahari, S. (2026) https://BioRender.com/q98c015**h** Flow cytometry analysis shows CD45.1 recruited donor cells (%) were maintained with FasL Ab treatment. In contrast, CD45.1 recruited donor cells (%) were reduced in the isotype control Ab-treated group. Data are expressed as mean ± SEM from 5 control young mice (*N* = 5), 6 control old mice (*N* = 6), and 8 experimental mice per group (*N* = 8), analyzed by ordinary one-way ANOVA (two-sided) with Sidak’s multiple comparisons test. **i** Another experimental approach aimed at depleting resident AMs by clodrolip and subsequent i.t. installation of FasL Ab together with CD11c^+^ AMs from young mice. BAL was conducted 2 days after AM and Ab administration, and donor AMs were analyzed by flow cytometry. Cartoon mouse in panel **i** was created in BioRender. Pahari, S. (2026) https://BioRender.com/x07c11. **j** Flow cytometry analyzed bar graph shows the phenotypic activation of CD11c^+^ donor AMs to a CD11c^+^CD11b^+^ phenotype in old but not young mice. Clodrolip-treated old mice treated with FasL Ab show less phenotypic change into CD11c^+^CD11b^+^ mAMs. The absolute number of CD11c^+^CD11b^+^ AMs recovered from BAL is shown in the bar graphs in Fig. S27b. Data are expressed as mean ± SEM from 3 control young mice, 3 control old mice, and 5 experimental mice per group (*N* = 5), analyzed by ordinary one-way ANOVA (two-sided) with Sidak’s multiple comparisons test. **k**, **l** Flow cytometry-generated bar graphs illustrate the induction of Caspase-8 and Caspase-3/7 mediated apoptosis in the isotype-treated groups, which was reduced following treatment with FasL Ab. Data are expressed as mean ± SEM from 5 control young mice (*N* = 5), 6 control old mice (*N* = 6), and 8 experimental mice per group (*N* = 8), analyzed by ordinary one-way ANOVA with Sidak’s multiple comparisons test (two-sided). **m** RNA-seq analysis revealed increased *Caspase-3* gene expression in CD11c^+^CD11b^+^ MoAMs from old mice compared to resident AMs (CD11c^+^ CD11b^−^) in both young and old mice. Data are representative of 3 independent experiments and are expressed as mean ± SEM, analyzed by ordinary one-way ANOVA with Sidak’s multiple comparisons test (two-sided). Each dot represents an individual experiment (*N* = 3), with 10 mice/group/experiment. **n** A schematic representation shows the extrinsic pathway of apoptosis, specifically those mediated by FasL, Caspase-8 and Caspase-3/7 in CD11c^+^CD11b^+^ MoAMs. Created in BioRender. Pahari, S. (2025) https://BioRender.com/f26m685. Each dot indicates an individual mouse/group. See also Figs. S25–S30.

As shown earlier (Fig. 4b), CD45.1 monocytes actively migrated into the alveoli and differentiated into MoAMs in old age. Peak migration occurs on Day 5 and following this peak, we noted a gradual reduction in the number of recruited MoAMs over time (Figs. 6b, c and S23). In contrast, the population of TRAMs remained stable throughout the observed period (Figs. 6d, e and S24). Based on these observations and induction of Fas expression on MoAMs (Figs. 1g, h and 7b), we hypothesized that recruited MoAMs have a transient existence characterized by rapid turnover, primarily driven by Fas-mediated apoptosis and continuous turnover of CD11c^+^CD11b^+^ MoAMs. Moreover, RNA-seq analysis of flow cytometry sorted CD11c^+^CD11b^+^ MoAMs revealed a significant increase in *Mmp2* (matrix metallopeptidase-2) and *Mmp14* (Fig. 6f, g), suggesting their involvement in cell migration and turnover within the alveoli. During cell migration, MMP-2 breaks down extracellular matrix components to enable movement, while MMP-14 activates MMP-2 at the cell surface, initiating the process^43^. RNA-seq data also showed upregulation of *Mmp9* gene expression in CD11c^+^CD11b^+^ MoAMs (Fig. 2d). Mmp9 cleaves the extracellular region of FasL (CD178), leading to the release of a soluble form of FasL (sFasL)^44^. Overall, our findings support the notion of a dynamic balance between resident and recruited AM populations in old age, where freshly recruited MoAMs exhibit high turnover due to the inflammatory environment they encounter.

### Role of Fas in monocyte recruitment to the lungs and controlling the inflammatory environment of the alveolus in old age

Our results indicate a significant increase in Fas receptor (CD95) expression in old mice (Fig. 1h and 7a, b) associated with turnover of recruited MoAMs. Fas signaling can induce the production of pro-inflammatory cytokines^45^, including those from macrophages, and higher Fas expression in the elderly correlates with an increase in inflammatory cytokines^45^. Chronic inflammation driven by Fas in older adults, particularly observed for alveolar epithelial cells, is associated with age-related diseases^46–48^. The role of resident and freshly immigrated AMs in Fas activation has not been explored.

We hypothesized that enhanced Fas-mediated signaling of resident AMs in old age creates a more favorable inflammatory environment for the recruitment of monocytes to the lungs, facilitating their differentiation into MoAMs (Fig. 7a, b). To test this, we combined Fas Ligand (FasL, CD178) neutralizing (NA/LE) Ab administered i.t. with the adoptive transfer of CD45.1 monocytes by i.v. on Day 0 in young and old mice (Fig. 7c). We observed that FasL neutralization effectively reduced Fas expression and the recruitment of CD45.1 MoAMs in old mice (Figs. 7d, e and S25). TNF levels increased significantly in the isolated day 5 BAL fluid of old mice, and administration of FasL Ab on day 0 effectively reduced TNF levels in these old mice (Fig. 7f). These studies suggested that Fas neutralization can reverse the inflammatory alveolar environment of old mice to more closely resemble the environment of young mice, leading to reduced MoAM recruitment.

We next hypothesized that the turnover of recruited MoAMs in old mice can be slowed by the addition of FasL-neutralizing Ab. To test this, we administered FasL Ab 5 days after the adoptive transfer of CD45.1 monocytes via i.v. [day in which recruited MoAMs is maximal (Figs. 6b, c and 7g)]. With Ab treatment, the recruited MoAMs were maintained for a longer period relative to those in the isotype control group (Figs. 7h and S26). These findings indicate that FasL signaling contributes to the turnover or loss of recruited MoAMs in the old age alveolar space, whereas blockade of FasL prolongs their persistence.

To further understand how Fas regulates the lung microenvironment, we depleted TRAMs by Clodrolip treatment and then added FasL neutralizing Ab, along with adoptively transferring TRAMs from young mice to young and old mice via i.t., then assessed the phenotype of donor AMs (Fig. 7i). Ab treatment had no effect on the freshly added TRAMs in young recipient mice. In contrast, Ab treatment nearly abolished the increased CD11c^+^CD11b^+^ AM population in the control group in old mice (Figs. 7j and S27).

### The role of FasL NA/LE Ab treatment in controlling AM apoptosis in old age

Our results on Fas/FasL expression and AM turnover indicated that one mechanism for the observed turnover is FasL-mediated induction of apoptosis^49,50^. The use of FasL NA/LE Ab may improve macrophage turnover and functionality by preventing premature apoptosis^13^. We extended the experimental approach noted above, where we combined FasL NA/LE Ab administration by i.t. with the adoptive transfer of CD45.1 monocytes via i.v. on Day 0 in young and old mice (Figs. 7c and S28a) to assess the degree of apoptosis using the Annexin-V assay. The results showed that the addition of FasL NA/LE Ab reduced the level of apoptosis in MoAMs (Fig. S28a–c), with the apoptotic pathway mediated through caspase 3/7 (Fig. S28d).

Fas-mediated apoptosis occurs through the extrinsic pathway rather than the intrinsic pathway^49^. Caspase 3/7 is an executor caspases involved in both pathways. Our results provide evidence that recruited MoAMs primarily undergo apoptosis through the extrinsic pathway, specifically those mediated by FasL and Caspase-8, followed by the downstream activation of Caspases-3/7 (Figs. 7k, l, S28 and S29). In concert with this, RNA-seq analysis of flow cytometry sorted CD11c^+^CD11b^+^ MoAMs revealed a significant increase in Caspase-3 (Figs. 7m and S30). Overall, our data provide evidence that enhanced Fas-mediated signaling in old age leads to extrinsic apoptosis and, consequently, high turnover of recruited MoAMs (Fig. 7n).

## Discussion

**Fig. 8:**
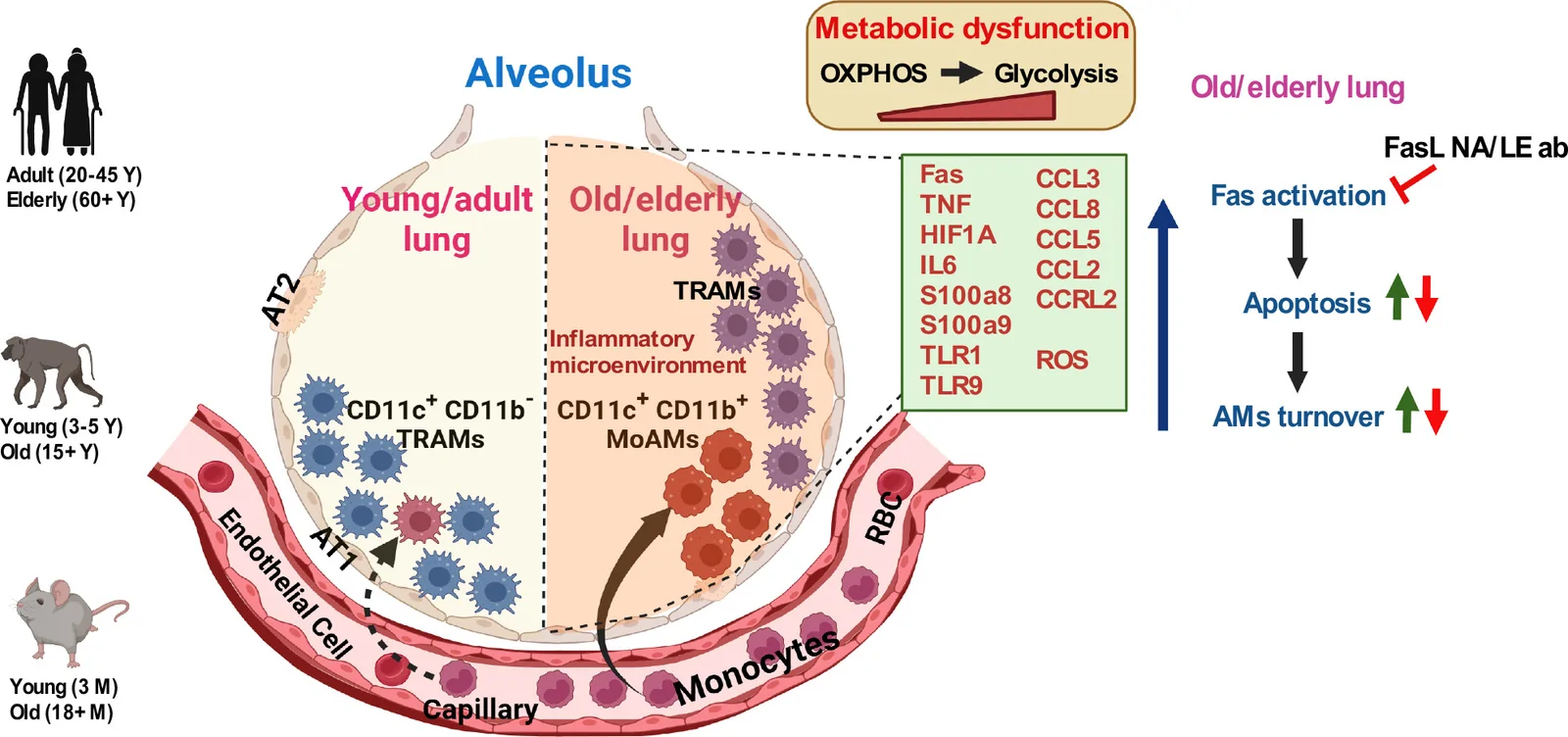
The pictorial model compares the lung alveolar space and alveolar macrophages (AMs) in young/adult versus old/elderly individuals. In young/adult lungs, the environment is less inflammatory, resulting in lower recruitment of CD11b^+^ Ly6C^+^ CD11c^−^ monocytes via the CCR2 receptor. Most resident AMs (TRAM) are CD11c^+^ CD11b^−^ phenotypically. In elderly lungs, inflammation increases due to molecules like Fas, CCL2, and CCL3, enhancing monocyte recruitment and leading to more monocyte-derived AMs (MoAMs) with a CD11c^+^ CD11b^+^ Ly6C^+^ phenotype. This shift occurs in mouse AMs (mAMs), human AMs (hAMs), and baboon/rhesus AMs (bAMs/rAMs). AMs show a metabolic shift from OXPHOS to glycolysis in old age. CCR2-deficient monocytes remain in circulation and cannot reach the alveolar region. Neutralizing Fas reduces inflammation and caspase-mediated apoptosis in mAMs and recruits fewer inflammatory cells. This model was created using BioRender. Pahari, S. (2026) https://BioRender.com/r77q644.

The impact of healthy aging on the nature of cellular function in tissue environments is significant, particularly in the lung, which is constantly exposed to inhaled particulates and microbes. This study offers important insights into how aging, in the absence of disease, affects the phenotype, recruitment, and turnover of AMs across mammalian species and lays the groundwork for developing interventions to mitigate respiratory diseases in older adults. A significant finding is the increased presence of CD11c^+^ CD11b^+^ MoAM subpopulations in healthy elderly participants, which exhibit elevated inflammatory, immunoregulatory, and cell death markers compared to the resident CD11c^+^ CD11b^−^ TRAMs. The inflammatory alveolar environment in old age can more effectively 1) phenotypically reprogram TRAMs from young to a CD11c^+^ CD11b^+^ phenotype in old age, and 2) recruit MoAMs from monocytes in the periphery that undergo continuous replenishment due to a relatively short half-life resulting from increased apoptosis in the inflammatory milieu (Fig. 8). This shift towards a more inflammatory AM phenotype underscores the role of aging as a driving force behind heightened inflammatory responses and likely contributes to the increased susceptibility to respiratory diseases in older individuals.

RNA sequencing analysis demonstrates significant transcriptional shifts in mAM subpopulations in healthy old age, particularly regarding CD11c^+^CD11b^+^ mAMs. These cells exhibit significant upregulation of inflammatory genes, e.g., *Ccl2, Ccl3, Ccl8, Ccrl2, Cd40, Hif1α, Il6, Tnf, S100a8, S100a9, Csf1, Mmps* and *Tlr1, 2, 9*. The identified inflammatory profile, characterized by increased expression of TNF signaling-related genes and pro-inflammatory cytokines, aligns with the chronic low-grade inflammation commonly observed in age-associated diseases such as COPD and asthma^51^. Another notable finding is the metabolic shift towards glycolysis in old age mAMs, consistent with the Warburg effect, supporting the energetic demands of their inflammatory role and may contribute to tissue remodeling in the lungs in old age. Quantitative PCR analysis of hAMs corroborates our mouse RNAseq data, revealing higher levels of inflammatory and cell death markers in elderly individuals, indicating common mechanisms of age-related inflammation across mammalian species. Our findings suggest that the expanded CD11c^+^CD11b^+^ AM population in old mice is not solely explained by a normal monocyte-to-macrophage maturation process. Instead, the old age alveolar niche appears to provide chronic inflammatory cues that reprogram TRAMs and promote recruitment and phenotypic programming of incoming AMs. The enrichment of inflammatory, senescence-associated, and metabolic stress pathways supports a model in which the old age lung microenvironment drives the accumulation of recruited macrophages with a distinct inflammaging-like transcriptional state.

As we age, baseline oxidative stress and inflammation significantly contribute to the development of an inflammatory microenvironment. These two factors are interconnected and can accelerate the aging process, increasing the risk of chronic diseases^52,53^. Increased baseline inflammation is linked to mitochondrial dysfunction and increased mitochondrial ROS production^54^. The metabolic reprogramming of mAMs in old mice, characterized by a shift from OXPHOS to glycolysis, has important implications for the heightened inflammatory responses within the lungs. Our findings align with studies that have demonstrated shifts in cellular metabolism with age in other cells and tissues. Previous research has shown that M1 macrophages or aged BMDMs display altered metabolic profiles, supporting our notion of a glycolytic shift contributing to inflammation^55,56^. Unexpectedly, we found that OXPHOS and glycolysis are both elevated in old mAMs, indicating that these pathways operate independently in mice. Extracellular flux and qPCR assays were performed on the total AM compartment rather than on individually sorted AM subsets. Therefore, the observed age-related differences in ECAR, OCR, and gene expression likely reflect both intrinsic metabolic changes and shifts in the relative abundance of resident versus recruited AM populations. While subset-specific (CD11c^+^CD11b^+^ cells) analyses would provide additional resolution, recovery of sufficient viable sorted cells for Seahorse assays was technically challenging. Future studies using low-input metabolic platforms will help define the bioenergetic properties of individual AM subsets during aging. The interplay between increased ROS production and mitochondrial dysfunction is consistent with previous work regarding the relationship between oxidative stress and inflammation^57^. Incorporating comparative studies across mammalian species provides a deeper understanding of the cellular dynamics at play and their common underlying mechanisms.

Adoptive transfer experiments provide evidence that with old age, the alveolar environment becomes less favorable for maintaining TRAM homeostasis, leading to the recruitment and activation of MoAMs. The Ms4a3^Cre^-Rosa^TdT^ fate mapper mouse model has proven instrumental in studying the origin of tissue macrophages^38^. The observed upregulation of Ms4a-related gene sets in MoAMs suggests that old age is associated with a shift in the immune landscape within the lungs, potentially driven by a chronic inflammatory microenvironment. Our studies corroborate those indicating that the aging process or exposure to other environmental insults alters the immune cell composition within the lungs^5,8,16,35,58^. In all, the findings further support the premise that an inflammatory microenvironment drives differentiation of circulating monocytes into specialized tissue macrophage populations^59,60^. PKH26-based adoptive transfer studies further refined our interpretation of macrophage dynamics in the healthy old age lung. These experiments indicate that the expanded CD11c^+^CD11b^+^ population in old mice reflects two complementary processes: phenotypic reprogramming of the transferred young TRAMs within the old age niche and increased recruitment of MoAMs into the alveolar space. Thus, rather than a complete lineage conversion, the old age alveolar environment promotes macrophage plasticity while simultaneously supporting the accumulation of recruited cells. Notably, this phenotypic shift was time-dependent, suggesting that longer residence within the old age alveolar niche may further increase acquisition of the CD11c^+^CD11b^+^ phenotype beyond that observed in the current study. It is important to note here that CD11b is not a stable lineage-specific marker for AMs, since its expression can be upregulated in response to activation or inflammatory signals. Together, these findings highlight the importance of the local lung microenvironment in shaping AM composition during aging.

Our adoptive transfer and fate-mapping studies support the increased capacity of bone marrow-derived monocytes to enter the old age alveolar niche and contribute to expanded MoAM populations. However, these approaches do not exclude the possibility that additional mechanisms, including local proliferation or long-term persistence of resident cells, also participate during physiological aging. Thus, the increased macrophage heterogeneity observed in old lungs is likely shaped by several complementary processes. Future studies using parabiosis or long-term chimera models will help define the relative contribution of circulating versus resident sources over time.

TRAMs lack CCR2 expression^42^, whereas CCR2 expression is high in monocytes in peripheral circulation^61^. We found that CD11c^+^CD11b^+^ MoAMs express CCR2. Competitive, adoptively transferred CD45.1 and CCR2-KO-GFP bone marrow monocytes revealed that CCR2-KO-GFP^+^ cells remain in circulation and do not reach the alveoli. These findings further support the concept that the old age lung provides a permissive niche for monocyte entry into the alveolar compartment. CD45.1^+^ donor cells efficiently populated the alveolar space in old recipients but were detected only at low frequency in young mice, indicating that recruitment is enhanced with age. This preferential accumulation in old mice is consistent with age-related changes in local chemokine gradients, niche availability, or inflammatory signals that favor monocyte-derived macrophage recruitment. This suggests that CCR2 is essential for the migration of MoAMs into the lungs (and likely to other organs) in healthy old age. Increased levels of circulating monocytes in elderly populations correlate with hyperactivated immune cells^62^. Our data provide evidence for the distinct role of CCR2 in monocyte migration in healthy old age. In contrast, we did not observe consistent differences in CX3CR1 protein levels among old/elderly individuals across species.

Our experiments reveal that old age leads to the recruitment of short-lived MoAMs, which are prone to rapid apoptosis via Fas activation through the extrinsic Caspase-8, 3/7 pathway. Thus, while recruitment of these immune cells is increased, their lifespan is constrained, with ongoing turnover, potentially as a strategy (albeit unsuccessful) to mitigate excessive inflammation. Our study focused on characterizing established migrated AM subsets within the alveolar space, rather than capturing monocytes immediately after their recruitment. Accordingly, CD11c^+^SiglecF-based gating enriches cells that have already acquired AM characteristics within the lung environment. Therefore, early SiglecF^−^ transitional populations may be underrepresented in our analysis. This is particularly relevant in aging, where recruitment and maturation occur in parallel, and our data primarily reflect AMs that have adapted to the alveolar niche. Future approaches using broader myeloid markers in interstitial tissue sections or lineage-tracing strategies will help better define these early transitional states during monocyte-to-macrophage differentiation in the lung.

Our findings regarding Fas (CD95), a member of the TNF receptor superfamily, contribute to the increasingly recognized role of Fas signaling in regulating apoptosis and inflammatory responses^63^ in the context of aging^64,65^. This contrasts with apoptosis in adults, which is considered anti-inflammatory and promotes antigen presentation and microbial control^66,67^. Previous research has demonstrated that Fas ligand (FasL)-mediated activation of Fas on immune cells, e.g., human monocytes and monocyte-derived macrophages or epithelial cell types during acute lung injury, leads to increased apoptosis and the production of pro-inflammatory cytokines^8,45,68^. In older individuals, Fas activation is associated with the dysregulation of immune homeostasis, which contributes to the chronic low-grade inflammation frequently observed in aging. Our evidence suggests that Fas signaling is crucial in maintaining the inflammatory environment in the lungs associated with old age, reinforcing the concept that Fas-induced apoptosis in immune cells is a significant contributor to age-associated inflammatory responses. Our findings emphasize the detrimental effects of Fas in the aging context, where its activation appears to skew the immune response toward chronic inflammation and accelerate immune dysfunction. Indeed, Yu et al. (2011) demonstrated that Fas-deficient mice exhibit decreased inflammatory cell infiltration and reduced production of pro-inflammatory cytokines in an acute spinal cord injury model^63,69^.

The implications of this research are significant, opening further potential therapeutic strategies aimed at improving immune function and tissue homeostasis in the healthy elderly population. By reducing the alveolar inflammatory environment and the presence of short-lived, apoptotic AMs, we may be able to reverse some age-associated changes within the lung, ultimately leading to healthier aging and lessening the incidence of age-related respiratory conditions.

### Limitations of the study

A practical limitation of this study is the low number of AMs recoverable from BAL in mice, which restricted some subset-specific transcriptomic and metabolic analyses. In particular, CD11c^+^CD11b^+^ AMs were rare in young mice (<1–2%), preventing recovery of sufficient cells for direct RNA-seq comparison with the expanded CD11c^+^CD11b^+^ population in old mice. Similarly, flow sorting yielded insufficient viable AM subsets for reliable Seahorse analysis in CD11c^+^CD11b^+^ populations. We therefore focused transcriptomic comparisons on CD11c^+^CD11b^−^ TRAMs from young and old mice and CD11c^+^CD11b^+^ AMs from old mice, while complementing these analyses with flow cytometry, imaging, BAL protein measurements, and pooled RNA-seq. Another limitation relates to the dynamic nature of AM depletion and replenishment in vivo. Following clodronate-mediated AM depletion, approximately 10–15% of the AM population remained detectable in the alveolar compartment. This residual population may reflect, at least in part, continuous recruitment and replenishment of immigrating cells during the depletion period, making it difficult to precisely distinguish residual cells from freshly recruited populations. Although our adoptive transfer and fate-mapping studies support contributions from both recruited monocytes and phenotypic changes in resident AMs, future parabiosis or long-term chimera studies will further define the relative contribution of circulating versus resident macrophage sources during aging.

This study focuses exclusively on comparing young and old age groups. It does not evaluate the effects of Fas in regulating Fas-mediated inflammatory conditions across the aging spectrum. Also, although we provide evidence that the Fas-mediated extrinsic pathway contributes to macrophage apoptosis associated with old age, we have not ruled out the involvement of the intrinsic apoptosis pathway. Finally, this study focuses on the lung alveolus and AMs; it did not examine the tissue immune response in other organs. Finally, future studies will need to directly link our findings to their impact on host susceptibility to airborne infections, which is our next goal.

## Methods

### Human participants and ethics statement

Human participant studies were conducted in strict accordance with the US Code of Federal and Local Regulations, as overseen by The Ohio State University (OSU) institutional review board with the number 2012H0135. The studies were also overseen by the Texas Biomedical Research Institute with IRB number HSC20170673H. Most samples were collected from the Division of Pulmonary and Critical Care Medicine, UT Health Science Center, San Antonio. Bronchoalveolar lavage (BAL) samples were collected from healthy adults (20–45 years) and elderly (≥60 years) individuals of both sexes, with an average age of adults (33 years) and elderly (69 years), without discrimination of race or ethnicity, following written consent (see supplementary Table S1). The donors had a preoperative diagnosis of lung nodule, lung nodule with mass, or abnormal parathyroid and thymus, without clinical features. BAL was performed on the healthy lobe of each donor’s lung, yielding a cell count range of 2–5 million. Individuals with specific comorbidities and health-related risk factors were excluded from the study to minimize confounding effects. Exclusion criteria included current smoking or smoking cessation within the past month, excessive alcohol use, recreational drug use (injection or non-injection), active pneumonia, asthma, bilateral cancer or ongoing chemotherapy, chronic obstructive pulmonary disease (COPD), prediabetes or diabetes (hemoglobin A1c > 5.7% within the previous three months or fasting blood glucose >110 mg/dL), body mass index (BMI) ≥ 40, hepatitis, human immunodeficiency virus (HIV/AIDS), immunosuppression or use of nonsteroidal anti-inflammatory biologics such as TNF antagonists, leukemia or lymphoma, liver failure, renal failure, rheumatoid arthritis, pregnancy or childbirth within the previous three months, recent antibiotic use, and regular use of over-the-counter anti-inflammatory medications. Each “N” value represents a different human BAL donor as specified in the figure legends.

### Mice

Specific pathogen-free C57BL/6 female mice were obtained from Charles River Laboratories (Wilmington, MA) at either 3 months of age (young) or 18 months of age (old) through a contract with the National Institute on Aging (NIA). Mice were housed in individually ventilated cages (IVC) and allowed to acclimate to the facility for one week before being used for the study. Mice were provided with Nestlets, Shepherd Shacks, sterile food (2920X irradiated diet), water (Braun Sterile Water for Irrigation), bedding (7097 A Teklad 1/4″ Corncob Bedding) and cages were changed weekly under sterile conditions within a biosafety cabinet. Mice were housed on a 12 h white light/12 h dark/red light cycle, at 68–79^◦^F ambient temperature with relative humidity of 30–70%. Animal health was routinely monitored by trained animal care personnel and a designated veterinarian at the Texas Biomedical Research Institute (Texas Biomed) animal facility. Animals exhibiting age-related health issues were humanely euthanized in accordance with institutional guidelines. All procedures were approved by Texas Biomed Institutional Laboratory Animal Care and Use Committee (Texas Biomed-IACUC number 1608-MU). In each experiment, mice were euthanized by gradual-fill carbon dioxide (CO₂) inhalation followed by a secondary physical method to ensure death, in accordance with the AVMA Guidelines for the Euthanasia of Animals: 2020 Edition. C57BL/6 young CD45.1 congenic mice (3 months old), B6.SJL-Ptprc^a^Pepc^b^/BoyJ (B6.CD45.1, Strain # 002014) and CByJ.SJL-Ptprc^a^/J (CD45.1, Strain # 006584) BALB/c mice were purchased from The Jackson Laboratory. Ms4a3^Cre^-Rosa^TdT^ mice bone marrow was acquired from Florent Ginhoux, Singapore Immunology Network (SIgN), Agency for Science, Technology and Research (A*STAR). Ccr2^gfp/gfp^ KI/KO mice (C57BL/6, Strain # 027619) were purchased from The Jackson Laboratory.

### Collection and isolation of human alveolar macrophages (hAMs)

To isolate and culture hAMs from BAL^70^ samples collected within 6 h were centrifuged (250 g) and washed twice in PBS at 4 °C. The cell pellet was then resuspended in RPMI 1640 with Penicillin-G (Pen-G stock diluted to 1:50 to achieve 10,000 U/mL), and cells were allowed to adhere for 2 h in 24-well tissue culture wells (Falcon/ Corning Life Sciences). After adherence, Pen-G was removed by washing. A portion of the cell suspension underwent cytospin, followed by staining and microscopy to determine the percentage of hAMs in BAL (>98%). Following cell counting on a hemocytometer, hAMs (1 × 10^5^) were plated for morphometric microscopic analysis. In a separate experiment, hAMs (5 × 10^5^) were immediately lysed in TRIzol reagent (Invitrogen) for qRT-PCR analysis. Another set of cells was utilized for flow cytometry for phenotypic analysis.

### Collection and isolation of baboon and Rhesus alveolar macrophages (bAMs; rAMs)

Olive baboons (*Papio anubis*) and Rhesus macaque (*Macaca mulatta*), both young (ages 3–5 years) and old (ages 15^+^ years) of both sexes, were obtained from our colony at the Southwest National Primate Research Center (SNPRC) at Texas Biomed. These animals were acquired through a biomaterials request, involving opportunistic BAL collection conducted without any disease conditions. This occurred during routine necropsies due to age or through live animal BAL collections, following Texas Biomed-IACUC Protocol number 1516 PC and 1516 MM. BAL was performed by experienced pathologists in 0.9% saline at the SNPRC-Pathology lab. AMs were collected through centrifugation at 300 × *g* for 10 min and washed. Depending on the experimental requirements, BAL cells from individual baboons were analyzed using cytospin staining, confocal microscopy or flow cytometry.

### Isolation of mouse alveolar macrophages (mAM) and Alveolar Lining Fluid (ALF)

Both young and old mice were euthanized using CO_2_ following an approved protocol (Texas Biomed-IACUC 1608-MU). AMs and BAL fluid (BALF) were obtained by washing the lungs of mice with sterile, endotoxin-free saline containing 100 µM EDTA (0.5 M), and either 0.2% BSA (for BAL fluid needed for Luminex assay) or 5% FBS. Prior to BAL collection, the isolation buffer was pre-warmed in a water bath and maintained at 37 °C. After washing the lungs 6–10 times with 0.5 ml of the saline solution, AMs were collected through centrifugation at 300 × *g* for 10 min. Depending on the experimental requirements, individual BAL cells were analyzed using confocal microscopy or flow cytometry. For qRT-PCR, BALs from 6 to 10 mice were pooled, and the RNA was isolated using TRIzol reagent (Invitrogen) for measuring basal RNA levels. The supernatant fraction containing ALF from each individual mouse was promptly frozen and stored at −80 °C until used for the Luminex assay.

### Cytospin analysis

Healthy adult and elderly hAMs were collected for cytospin analysis. Shandon non-coated cytoslides (Thermo Scientific) were placed in a Shandon EZ single cytofunnel with white filter cards. A single cell suspension was created, and 200 μL of cells (5 × 10^4^) were placed into the cytofunnel and centrifuged at 150 × *g* for 5 min using Program 1. The cells on cytoslides were dried and then stained with HEMA 3 differential staining (Fisher Health Care). Afterward, the slides were washed with water and dried. The slides were placed in Hema 3 fixative solution for 30 s. They were then dipped 30 times for 1 s each in Hema 3 Solution I (eosin Y) and subsequently dipped 30 times for 1 s each in Hema 3 Solution II (methylene blue). It was important to allow excess stains to drain during each step. After staining, the slides were washed with water and left to dry. Hema 3 Solution I (eosin Y) was used to stain cytosolic proteins, while Hema 3 Solution II (methylene blue) stained the nuclear membrane. Finally, cells were examined using a Motic AE2000 microscope.

### Isolation of mouse monocytes and adoptive transfer

Bone marrow cells (BMCs) from the femurs and tibias of C57BL/6 wild-type, CD45.1, Ms4a3^Cre^-Rosa^TdT^, and Ccr2^gfp/gfp^ KI/KO young mice were flushed aseptically. The Ly6C-positive monocytes were isolated by negative selection using the EasySep Mouse Monocyte Isolation Kit (Cat#19861RF, Stem Cell Technologies, Cologne, Germany), according to the manufacturer’s instructions. The isolated monocytes were then processed for the adoptive transfer experiments.

To study macrophage turnover, monocytes from B6.CD45.1 young mouse (donor cells) were injected intravenously (tail vein injections, i.v.) [1.5 × 10^6^ donor monocytes/mouse] in recipient young or old C57BL/6 mice (CD45.2). After 0, 3, 5, 7, and 10 days, depending on the experimental strategy shown in the Figs. (4–6), 5–8 recipient mice in each group were sacrificed, and AMs were obtained by BAL to perform flow cytometry to verify cell turnover. The immigrated MoAMs were distinguished through CD45.1 cell labeling, while resident AMs were identified from CD45.2-labeled cell populations.

To identify the origin of recruited cells in the alveolar region and parenchyma (Figs. 4 and S19), the fate mapper model (Ms4a3^Cre^-Rosa^TdT^) was used for adoptive transfer to track monocytes and their bone marrow progenies^38^. The Ms4a3^Cre^-Rosa^TdT^ cells utilize a Cre-loxP recombination system, where the Ms4a3^Cre^ promoter drives Cre recombinase expression, leading to activation of the TdTomato (TdT) reporter in specific cell lineages. The purified young mice Ms4a3^Cre^-Rosa^TdT^ bone marrow monocytes (donor cells) were adoptively transferred (1.5 × 10^6^ donor monocytes/mice) via i.v. in recipient young or old C57BL/6 mice. On day 5, BAL cells were isolated to analyze recruited cells in the alveolar space by flow cytometry, confocal microscopy and histopathological analysis. For histopathological analysis, entire lung lobes were collected from the recipient mice. The lung lobes were preserved in 10% formalin and sent to the Texas Biomed path lab for histopathological analysis by H & E staining. The recruited dense, accumulated cells (blue hematoxylin-stained cells) and Rosa TdTomato-labeled cells were identified through HALO analysis.

To assess the role of CCR2 in cell recruitment in the alveolar region and parenchyma (Fig. 5), purified Ccr2^gfp/gfp^ knockout mice bone marrow monocytes were adoptively transferred (1.5 × 10^6^ donor monocytes/mice) through the i.v. route into young or old C57BL/6 recipient mice. On day 5, during the necropsy, blood was collected from the heart using heparin (1000 U/mL) to prevent coagulation. PBMCs were isolated from the blood of recipient mice using Ficoll-Hypaque density cushion centrifugation, while AMs were isolated by BAL. The presence of GFP^+^ cells was confirmed in both BAL cells and blood-derived cells. Cells (6 × 10^4^/ 200 μL) were then placed in a cytofunnel and centrifuged in the Shandon Cytospin 4 at 150 × *g* for 5 min using Program 1. Subsequently, the cells were fixed with 2% paraformaldehyde for 3 min. The slides were washed three times with 1X DPBS (Gibco) and mounted on coverslips with Prolong Gold, along with DAPI (Thermo Fisher Scientific). The stained slides were examined using a Zeiss LSM 800 confocal microscope (20x, 63x magnification). Mean fluorescence intensity (MFI) and percentage of positive cells were quantified using ZEN 2.3 blue edition (Zeiss) and ImageJ software (NIH, Bethesda, MA).

### Depletion of AMs in mice by treatment with clodronate liposomes

Young and old mice were treated with Clodronate liposomes or control liposomes (Liposoma research, Cat#CP-005-005) at a dose of 50 µL/mouse (250 µg/mouse) via the i.t. route on day 0 and 3 (two doses). On days 3, 5, 6, and 7, the cells were isolated by BAL. The cells were counted, and cytospin analysis was performed to demonstrate the percentage of AMs that are depleted from the alveolar space. For experimental purposes, TRAMs from a separate set of young mice (donor mice) were isolated by BAL. On day 5, PKH26 lipophilic dye-labeled TRAMs (5 × 10^5^ cells) were intratracheally (i.t.) instilled into clodronate-treated young and old recipient mice. On day 7, BAL was performed on young and old recipient mice to assess the impact of the lung environment on changes in the phenotype of TRAMs (donor young mice) in the alveolar space of young and old mice. We assessed both PKH26-labeled donor cells and unlabeled cells that had immigrated into the alveolar space during this time for their phenotypic changes in response to the inflammatory milieu of the old-age alveolar microenvironment.

### FasL NA/LE Ab treatment in mice

For the FasL (CD178) NA/LE Ab treatment study, donor bone marrow monocytes (1.5 × 10^6^ donor monocytes/mice) were isolated from B6.CD45.1 mice and adoptively transferred via the i.v. route in recipient young or old C57BL/6 mice (CD45.2). After day 0 or day 5, depending on the experimental strategy shown in Figs. 7 and S25–S30, FasL NA/LE Ab was i.t. installed to block the Fas-mediated inflammatory response^63,71^. After 0, 3, 5, 7, and 10 days, depending on the experimental strategy shown in Figs. 7 and S25–S30, 5 to 8 recipient mice in each group were sacrificed, and AMs were obtained by BAL to perform a flow cytometry assay. Fas (CD95)-mediated apoptosis via Caspase 3, 7 and 8, as well as cell turnover were verified.

### Confocal microscopy

For confocal microscopy analysis, Ms4a3^Cre^-Rosa^TdT^ (Red), CD45.1 and Ccr2^gfp/gfp^ KI/KO (Green) positive cells (BAL and blood derived) (6 × 10^4^/ 200 μL) were placed in a cytofunnel and centrifuged in the Shandon Cytospin 4 at 150 × g for 5 min using Program 1. For CD45.1 labeling, cells were stained with anti-mouse CD45.1-PE antibody and incubated for 40 min in the dark at 4 °C. After washing with cell staining buffer, cells were stained with DAPI (400 ng/ml) for 10 min in the dark at room temperature to label nuclei. Subsequently, the cells were fixed with 2% paraformaldehyde for 3 min. Slides were washed three times with 1x DPBS (Gibco) and mounted on coverslips with Prolong Gold or Prolong Diamond (Thermo Fisher Scientific). The stained slides were examined using a Zeiss LSM 800 confocal microscope (20×, 63× magnification). The Percentage of GFP and CD45.1-positive cells was quantified using ZEN 2.3 blue edition (Zeiss) and ImageJ software (NIH, Bethesda, MA).

### Annexin V and EthD-2 cell viability assay

Monocytes were taken from B6.CD45.1 mice and 1.5 × 10^6^ donor monocytes were i.v. injected (via tail vein injections) into recipient young or old C57BL/6 mice (CD45.2). On day 0, FasL (CD178) NA/LE antibodies were administered directly into the lungs to block Fas (CD95)-mediated apoptosis. On days 3 and 4, recipient mice in each group were sacrificed, and AMs were obtained by BAL to perform a flow cytometry viability assay. The cells were then suspended in Annexin binding buffer [0.01 M HEPES (pH 7.4), 0.14 M NaCl, and 2.5 mM CaCl_2_]. APC-labeled Annexin V (4 μL per tube) was added to all samples and incubated for 15 min in the dark at room temperature. After washing, Ethidium Homodimer-2 (EthD-2, 4 µM) was added and incubated for another 10 min at room temperature. The cells were fixed with 2% paraformaldehyde (PFA). After washing with 1% binding buffer (400 μL), cells were analyzed by the BD FACS Symphony instrument immediately, and the data were analyzed using FlowJo software.

### Flow cytometry

AMs (1–2 × 10^5^) were placed into FACS tubes (Falcon round-bottom polypropylene tubes, Cat# 352063) and centrifuged at 250 × *g* for 10 min. The cell pellets were then resuspended in 50 µL of cell staining buffer from Biolegend (Cat# 420201) along with TruStain FcX PLUS (anti-mouse CD16/32) Antibody (Ab) from Biolegend (Cat# 156604) for FC receptor blocking. After a 30 min incubation on ice, the cells were stained with fluorochrome-tagged antibodies and corresponding isotype-matched control antibodies in BD Horizon Brilliant Stain Buffer Plus (BD, Cat#566385) for 40 min in the dark at 4 °C. Following this, the cells were washed once, fixed in 2% paraformaldehyde (Thermo Scientific, Cat# J19943-K2) in cell staining buffer for 10 min, centrifuged (250 × *g* for 10 min), and then resuspended in 300 µL cell staining buffer. Cells were then filtered through a round-bottom polystyrene tube with a cell strainer snap cap (Falcon, Cat#352235). Samples were then acquired using a BD FACSymphony multi-color flow cytometer, and compensation, analysis, and data visualization were carried out using FlowJo 10.8.1 software (BD Biosciences). Instrument voltages were kept consistent across samples within each experiment. Isotype and “Fluorescence minus one” controls were utilized as needed to establish gates. The gating strategy employed for macrophage analysis is depicted in Figs. S2–S30.

The fluorochrome-tagged antibodies were purchased from BD Biosciences, San Jose, CA, and Biolegend, San Diego, CA. The list of mouse Abs were: BV421 anti-mouse/human CD11b (clone M1/70, Cat# 101236), APC-Cy7 anti-mouse CD11c (clone N418, Cat#117324), BV786 anti-mouse CD64 (clone X54-5/7.1, Cat#741024), BUV395 anti- mouse CD95 (Fas, Clone Jo2, Cat#740254), BUV496 anti-Mouse CD192 (CCR2, Clone 475301, Cat#750043), Alexa Fluor 488 anti-mouse CD45.1 (Clone A20, Cat#110718), PerCP/Cyanine5.5 anti-mouse CD45.1 (Clone A20, Cat# 110728), CellEvent Caspase-3/7 Green Flow Cytometry Assay Kit (Cat# C10427). The antibodies and their respective isotypes, sources, clones, and dilutions are listed in the “Key resources Table S2”.

### Luminex multiplex analysis

The release of cytokines, chemokines, and other secretory factors in the culture supernatant was measured using Luminex assays. The following analytes were measured using the Luminex mouse Discovery Assay (10-Plex, code mxmbhKZn) LXSAMSM-10kit (R&D Systems, Inc.): GM-CSF, TNF-alpha, S100A9, S100A8, CCL2/JE/MCP-1, Fas Ligand/TNFSF6, IL-1 beta/IL-1F2, MMP-2, CCL3/MIP-1a, and IL-10. BAL was performed, and the BALF and AMs were separated by centrifugation at 250 × *g* for 10 min. The 4 to 5 mL BALF was concentrated by passing it through a 10 kDa filter (Amicon, EMD Millipore, MWCO 10 kDa, Ref. UFC9010) and then centrifuged at 2800 × *g*, at 4 °C, for 30 min with a brake at 2. The upper concentrated ALF was collected, and a protease inhibitor cocktail (A + L) was added to prevent protein degradation. Selected analytes were measured from ALF (1:2 dilutions in 50 µL) in the Luminex 100/200 System (Luminex Corporation) following the manufacturer’s protocol. Data were analyzed using Belysa Immunoassay Curve Fitting Software (Millipore Sigma). Analytes/mL concentrations were calculated and plotted.

### Cell sorting using flow cytometry for bulk RNA-seq analysis

AMs were isolated from both 10 young and 10 old mice and then sorted into CD11c^+^ CD11b^-^ populations from young and old mice, and CD11c^−^ CD11b^+^ and CD11c^+^ CD11b^+^ populations from old mice using the BD FACSAria II flow cytometer. CD11c^−^CD11b^+^ populations were found in very low numbers and displayed a transcriptional signature like the CD11c^+^CD11b^+^ populations. Consequently, we did not include them in further analyses. Samples were collected in three different batches as three replicates for bulk RNA-seq. This was achieved by flow cytometry staining and sorting, using BV421 anti-CD11b (clone M1/70) and PE anti-CD11c (clone N418) antibodies. The post-sorted cells were then verified based on the gating strategy, and the purity of the populations was confirmed through gating.

### Bulk RNA-Seq from flow-sorted populations and analyses

Flow-sorted CD11c^+^ CD11b^-^ AMs from young mice and CD11c^+^ CD11b^−^ and CD11c^+^ CD11b^+^ AM populations from old mice were collected and washed at 250 × *g* for 10 min with 1% PBS. The CD11c^+^ CD11b^+^ AM population was rare in young mice, and insufficient numbers of flow-sorted cells were recovered to perform RNA sequencing. The RNA was isolated using TRIzol reagent (Invitrogen) and a Direct-zol RNA Microprep kit (Zymo Research) according to the manufacturer’s instructions. The isolated RNA was quantified using the Qubit 4 Fluorimeter (Invitrogen), and its quality was assessed with the 4200 TapeStation System (Agilent). Samples with an RNA integrity number (RIN) higher than 7 were used for RNA-seq. RNA-seq libraries were prepared from 300 ng of total RNA using the NEB Next RNA Ultra Kit (Qiagen, Redwood City, CA) with poly(A) enrichment, and 50-bp single-read sequencing with approximately 25–30 M reads per sample. The RNA sequencing was performed at the Genome Sequencing Facility (GSF) at UT Health San Antonio using the HiSeq 3000 platform (Illumina).

The raw sequence reads were performed using Trim Galore! to remove adapters and low-quality sequences^72^. Then, the trimmed reads were mapped to the mouse mm10 reference genome using HISAT2^73^. After that, read counts for each sample were obtained using featureCounts^74^. The next step involved conducting differential gene expression analysis using DESeq2^75^ with control of the false discovery rate (FDR) using the Benjamini-Hochberg procedure. Shrunken log2 fold changes (LFCs) were calculated using the adaptive shrinkage (ash) estimator with an Empirical Bayes approach. Genes with FDR-adjusted *P* value < 0.05 and LFC of more than 1 or less than −1 were considered differentially expressed. Finally, heat maps of specific genes were generated using the pheatmap package in R.

### RNA isolation, quantification and qRT-PCR

hAMs from adult and elderly donors or mAMs from young and old mice were collected by BAL. In select experiments, mAMs were cultured with Pam_3_CSK_4_ (100 ng/mL) for 24 h. Cells were washed at 250 × *g* for 10 min with 1% PBS (Gibco) and then treated with TRIzol reagent (Invitrogen). RNA was extracted using a Direct-zol RNA Microprep kit (Zymo Research) according to the manufacturer’s instructions and quantified using the Qubit 4 Fluorimeter (Invitrogen). The precipitated RNA was reconstituted with DNase/RNase-free water and reverse transcribed using random primers and SuperScript III Reverse Transcriptase (Invitrogen). cDNA synthesis using reverse transcriptase was carried out at 65 °C for 5 min, followed by 25 °C for 5 min, 50 °C for 60 min, 70 °C for 15 min, and then cooled to 4 °C. mRNA expression was analyzed using quantitative real-time RT-PCR (qRT-PCR) with TaqMan Universal PCR Master Mix (Applied Biosystems). The amplification conditions were selected at 50 °C for 10 min, 95 °C for 10 min, followed by 40 cycles of 95 °C for 15 s and 60 °C for 60 s in the Applied Biosystems 7500 Real-Time PCR System. Thermo Fisher Scientific, the TaqMan human primers with the best coverage and most citations were used. Relative expression was calculated using the ΔΔCT method, with β-actin (*ACTB*) as the housekeeping gene. The selected genes for humans are *MRC1, CD11B, CD11C, TLR2, NOS2, HIF1A, PIEZO1, FAS, CX3CR1, CCR2*. Mouse *Hif1α, Cox2, Cd74, Mif, Glut1, Hk2, Pfkb3, Gls1, Slc25a, Acly*, and *Actb* were analyzed for qRT-PCR expression using IQ SYBR Green Supermix (BioRad). β-actin (*Actb*) was used as the housekeeping gene. Validated mouse primers listed on PrimerBank^76^ were used, and primer sequences are listed in Key Resources Table S2.

### Extracellular flux analysis (Agilent Seahorse)

The real-time cell metabolism of mouse AMs was measured using a Seahorse XF Extracellular Flux Analyzer XFe96 (Agilent Technologies). This involved determining the oxygen consumption rate (OCR, pmol/min) and extracellular acidification rate (ECAR, mPh/min) according to the manufacturer’s instructions. To investigate the metabolic changes in old mAMs, we assessed mitochondrial respiration, ATP production, OCR, and ECAR using the Seahorse XFe96 analyzer. For the mitochondrial stress assays, mAMs were placed in a supplemented XF assay medium, followed by sequential injections of oligomycin (O), FCCP, and rotenone/antimycin (R/A). Under the same setup, injections of O and R/A alone were used to determine the ATP production rate specifically from mitochondria. 3-month-old and 18-month-old mice (5 × 10^4^/well) were adhered in 96-well Seahorse plates for 2 h. The cells were then washed and replenished with XF DMEM Seahorse media supplemented with 25 mM D-Glucose, 1 mM Sodium pyruvate, and 2 mM L-glutamine. After incubating in a non-CO_2_ incubator at 37 °C for 1 h, the basal levels of OCR and ECAR were measured. A Mito stress assay was performed with sequential addition of 5 μM oligomycin (an ATP synthesis inhibitor), 4 μM carbonyl cyanide 4-(trifluoromethoxy) phenylhydrazone (FCCP, an uncoupling agent), and 2 μM rotenone and antimycin A (inhibitors of complex I and III of the respiratory chain). For glycolysis stress analysis, cells were injected with 2 μM rotenone and 2 μM antimycin A, followed by 100 mM 2-deoxyglucose (2-DG) to determine glycolytic rate.

Each of these components has specific roles. Oligomycin decreases electron flow through the ETC, verifying that the basal OCR measurement impacts mitochondrial respiration or OCR. FCCP interrupts the mitochondrial membrane potential, resulting in continuous electron flow through the ETC and maximal oxygen consumption by complex IV. The spare respiratory capacity (SRC) was calculated by measuring the difference between FCCP-induced maximal respiration and basal respiration. The third injection uses a combination of rotenone and antimycin A to block mitochondrial respiration, enabling the measurement of non-mitochondrial respiration in the cells. In the ECAR glycolytic rate assay, glycolytic pathway inhibitor 2-DG is used to inhibit glycolysis through competitive binding to glucose hexokinase.

Because the CD11c^+^CD11b^+^ population was limited in number, especially after BAL recovery from young and old mice and flow sorting, obtaining sufficient viable cells for Seahorse extracellular flux analysis was technically challenging. In addition, the prolonged workflow involving BAL isolation, antibody staining, sorting, and assay preparation reduced cell responsiveness. Therefore, Seahorse measurements were performed using the total AM compartment rather than individually sorted subsets.

### MitoSox assay for mitochondrial ROS detection

To assess the production of mitochondrial reactive oxygen species (ROS) in AMs from both young and old mice, cells were stained with 5 μM MitoSOX (Thermo Fisher Scientific, Inc.) for 30 min. After staining, the cells were washed with cell staining buffer and centrifuged at 250 × g for 10 min at room temperature. Following centrifugation, the stained cells (5 × 10^4^/ 200 μL) were loaded into a cytofunnel and centrifuged in the Shandon Cytospin 4 at 150 × *g* for 5 min using Program 1. The cells were then fixed with 2% paraformaldehyde for 3 min. Subsequently, the slides were washed three times with 1X DPBS (Gibco) and mounted on coverslips with Prolong Gold containing DAPI (Thermo Fisher Scientific). The stained slides were visualized using a Zeiss LSM 800 confocal microscope at 20x and 63x magnification. Mean fluorescence intensity (MFI) and percentage of positive cells were quantified using ZEN 2.3 blue edition (Zeiss) and ImageJ software (NIH, Bethesda, MA).

### Chromogenic immunohistochemistry of mAMs

mAMs were isolated from BAL of young and old mice and cytospun onto positively charged glass slides to generate monolayer preparations. The slides were immediately fixed in methanol at −20 °C for 10 min, air dried, and stored at 4 °C until staining if not processed immediately. Multiplex chromogenic immunohistochemistry for CD11b and CD11c was performed using the Roche Ventana Discovery Ultra IHC/ISH automated staining platform (Roche Diagnostics). Endogenous peroxidase activity was blocked using Discovery Inhibitor (cat. #760–4840) for 8 min. For CD11b detection, slides were incubated with anti-CD11b rabbit polyclonal antibody (Invitrogen, cat. #PA5-79533; 1:500 dilution) for 1 h at ambient temperature. Detection was carried out using Discovery anti-Rabbit HQ (cat. #760–4815) for 8 min at 36 °C, followed by Discovery anti-HQ HRP (cat. #760–4820) for 8 min at 36 °C. Signal was visualized using the Discovery Purple chromogen kit (cat. #760-229) for 32 min. Slides were then denatured using ULTRA Cell Conditioning Solution (CC2) (cat. #950-223) at 95 °C for 8 min prior to the second stain. For CD11c detection, slides were incubated with anti-CD11c (D1V9Y) rabbit Ab (Cell Signaling Technology, cat. #97585; 1:150 dilution) for 1 h at ambient temperature. Detection was performed using the same anti-Rabbit HQ and anti-HQ HRP reagents as above. Signal was visualized using the Discovery Yellow HRP chromogen kit (cat. #760-250) for 72 min. Slides were counterstained with Hematoxylin (cat. #760–2021) for 16 min followed by Bluing Reagent (cat. #760–2037) for 4 min. Whole-slide brightfield images were acquired using the Zeiss Axio Scan.Z1 slide scanner (Carl Zeiss, Germany) and quantified using HALO image analysis software (Indica Labs, Albuquerque, NM, USA) and ZEN 2.3 blue edition software.

### Statistics

Graphs were created, and statistical comparisons were conducted using GraphPad Prism version 10. An unpaired two-tailed Student’s t-test was used for statistical comparisons of two groups. Where applicable (as indicated in figure legends), ordinary one-way and two-way analysis of variance (ANOVA), two-sided with Sidak’s and Tukey’s multiple comparisons test (GraphPad Prism vr 10) were applied for multiple testing. Spearman’s rank test was used for Correlation analysis. Statistical significance is reported for *P* value of ≤ 0.05. Precise *p*-values are reported in the figures. Data are presented as mean ± SEM for individual mice/donors and mean ± SD for representative data plots.

### Reporting summary

Further information on research design is available in the Nature Portfolio Reporting Summary linked to this article.

## Supplementary information

Supplementary Information

Description of Additional Supplementary Files

Supplementary Data 1

Supplementary Data 2

Reporting Summary

Transparent Peer Review file

## Source data

Source Data

## Data Availability

All data associated with this study are presented in this paper and are located either in the results section or in the supplementary files. Requests for further details on the dataset, resources, protocols, and reagents can be directed to the corresponding authors. Raw data generated during this study is provided with this paper as a separate Excel file labeled “Source Data file” and in the Supplementary Information. The RNA-seq data generated in this study have been deposited in NCBI’s Gene Expression Omnibus database under accession number: GSE297392. Source data are provided with this paper.
