# Supplementary material for: Lung environment in healthy old age shapes the phenotype and CCR2-mediated recruitment of a subset of apoptotic, high-turnover alveolar macrophages
Source: Nat Commun. 2026 Oct 7;17:11399. doi: 10.1038/s41467-026-78156-x (PMC13736887; doi:10.1038/s41467-026-78156-x)
Supplement: Supplementary file 1 [file 41467_2026_78156_MOESM1_ESM.pdf]

Lung environment in healthy old age shapes the phenotype and CCR2-mediated recruitment of a subset of apoptotic, high-turnover alveolar macrophages

Susanta Pahari[1]**, Miranda Lumbreras[1, 2], Arkajyoti Paul[1], Abul Azad[1], Hao Zhang[3], Hong Cai[3], Zhao Lai[4], Diego Jose Maselli[5], Jay Peters[5], Jordi B Torrelles[1,6], Joanne Turner[7], Florent Ginhoux[8], William P. Lafuse[9], Yufeng Wang[3], Eusondia Arnett[1], Larry S. Schlesinger[1]*

1. Host Pathogen Interactions and Population Health programs, International Center for the Advancement of Research & Education (*I•CARE*), Texas Biomedical Research Institute, San Antonio, TX, USA

2. Duke University School of Medicine, Durham, NC, USA

3. Department of Molecular Microbiology and Immunology, South Texas Center for Emerging Infectious diseases, University of Texas at San Antonio, San Antonio, TX, USA

4. Department of Molecular Medicine, UT Health San Antonio, San Antonio, TX, USA

5. Division of Pulmonary and Critical Care Medicine, UT Health Science Center, San Antonio, TX 78207

6. Integrated Research Center for Infectious Diseases, Division of Infectious Diseases, Department of Internal Medicine, and Infectious Diseases Institute, The Ohio State University, Columbus, OH 43210

7. Abigail Wexner Research Institute at Nationwide Children's Hospital,700 Children's Drive Columbus, OH 43205

8. Singapore Immunology Network (SIgN), Agency for Science, Technology and Research (A*STAR)

Singapore 138648

9. Department of Microbial Infection and Immunity, The Ohio State University, Columbus, OH 43210

This Supplementary information includes:

Supplementary Figures 1-30, and Supplementary Table 1-2

Figure S1

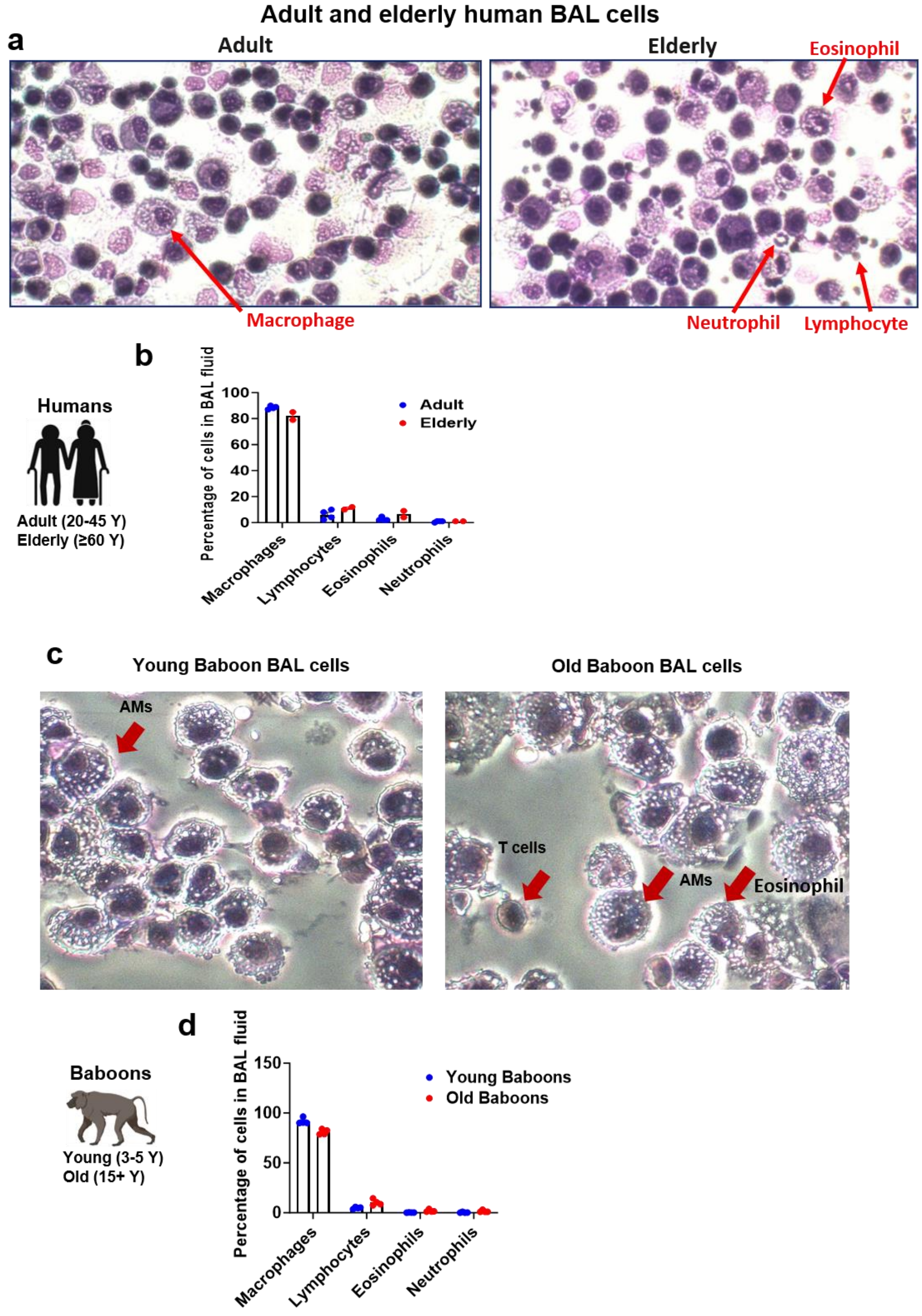

Figure S1. Cytospin analysis of BAL cells from adults and elderly individuals. (a) Cells were isolated from BAL samples of healthy volunteers: adults (ages 20-45) and elderly individuals (age 60 and older). Morphology of these cells was assessed using Wright-Giemsa staining to analyze the presence of various cell populations, including AMs, lymphocytes, neutrophils, and eosinophils, comparing elderly samples to those from adults. The red arrow highlights the identified cell populations. (b) The different cell populations were counted and plotted. The bar graph demonstrates that more than 80% of the population consists of

AMs. Approximately 10% are T cells, less than 5% are neutrophils and less than 1% eosinophils in the elderly donors. Data are presented from 4 adult individuals (N=4) and 2 elderly individuals (N=2). (c) Wright-Giemsa staining of AMs from both young and old baboons is presented. The red arrow indicates different cell types. The morphology of the AMs from young baboons was compared to that of AMs from old baboons after staining cytospin cells and observed using light microscopy. (d) Differential cell counts in the BAL of old baboons, a slight increase in T cells and other cell types was noted. The data were obtained from 4 young baboons (N=4) and 4 old baboons (N=4). The human and baboon cartoon schematics were created in BioRender. Pahari, S. (2025) https://BioRender.com/x07c111.

Figure S2

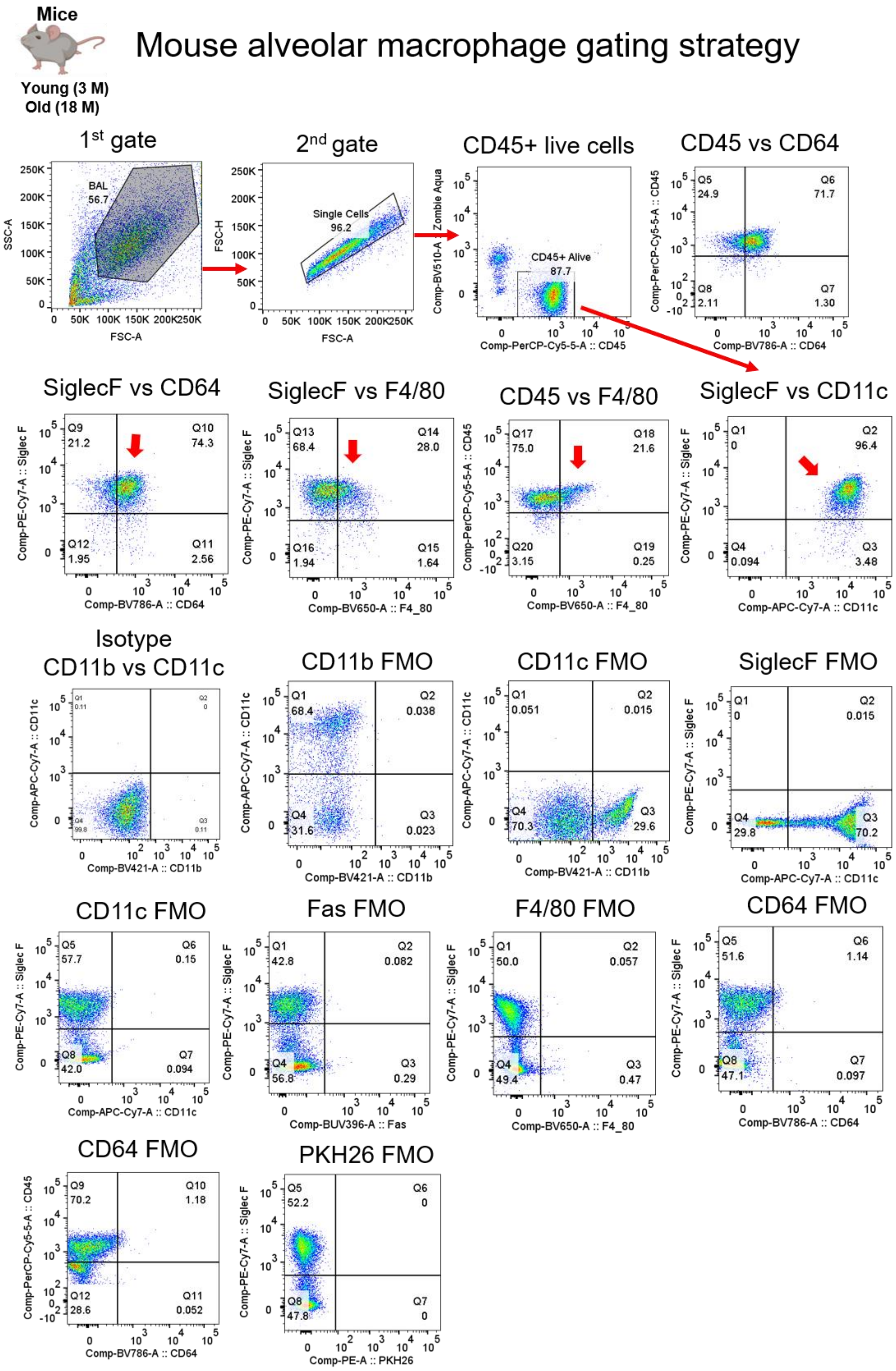

Figure S2. Flow cytometry gating strategy of mAMs (Figures 1, 3, 6, & 7). The initial gate was to identify the macrophage populations. The 2nd gate shows single cell selection. The next gate selects CD45^{+} live cells which were then sorted by SiglecF versus CD11c, or alternatively, by CD45 versus CD64, or by SiglecF versus CD64, or Siglec-F versus F4/80, or by SiglecF versus CD45. Both Isotype and FMO control were used to select the specific gated populations. After this, the following markers were used for selection: CD11b, CD11c, Fas (CD95), CCR2, CX3CR1, CD45.1, CD45.2, Ms4A3^{Cre}-Rosa^{TdT}, Caspase-3/7, Caspase-8, CD64, and F4/80. We assessed the percentage of positive cells and MFI. The mice cartoon schematic was created in BioRender. Pahari, S. (2025) https://BioRender.com/x07c111.

Figure S3

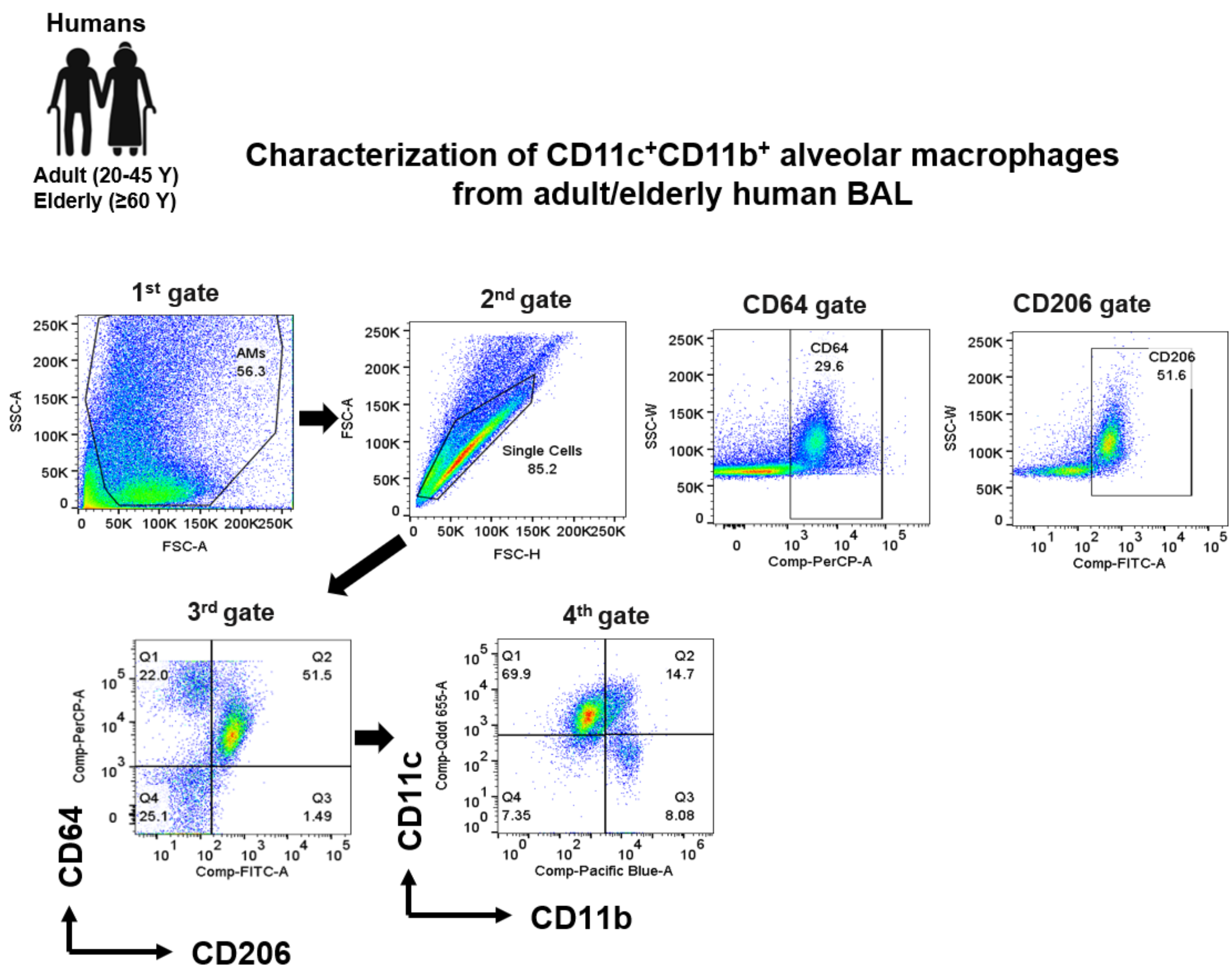

Figure S3. Flow cytometry gating strategy of hAMs (Figures 1). The early step in the gating strategy was to identify the macrophage populations within BAL cells. The second gate was used to select single-cell populations and separate cell clumps. The next gate focused on selecting CD64^{+} and CD206^{+} cells, which were then further analyzed based on the following cell surface receptors: CD64, CD11b, CD11c, CD206, CCR2, CX3CR1, Fas (CD95), HLA-DR, CD14, and CD16. We assessed the percentage of positive cells and their MFI. The human cartoon schematic was created in BioRender. Pahari, S. (2025) https://BioRender.com/x07c111.

Figure S4

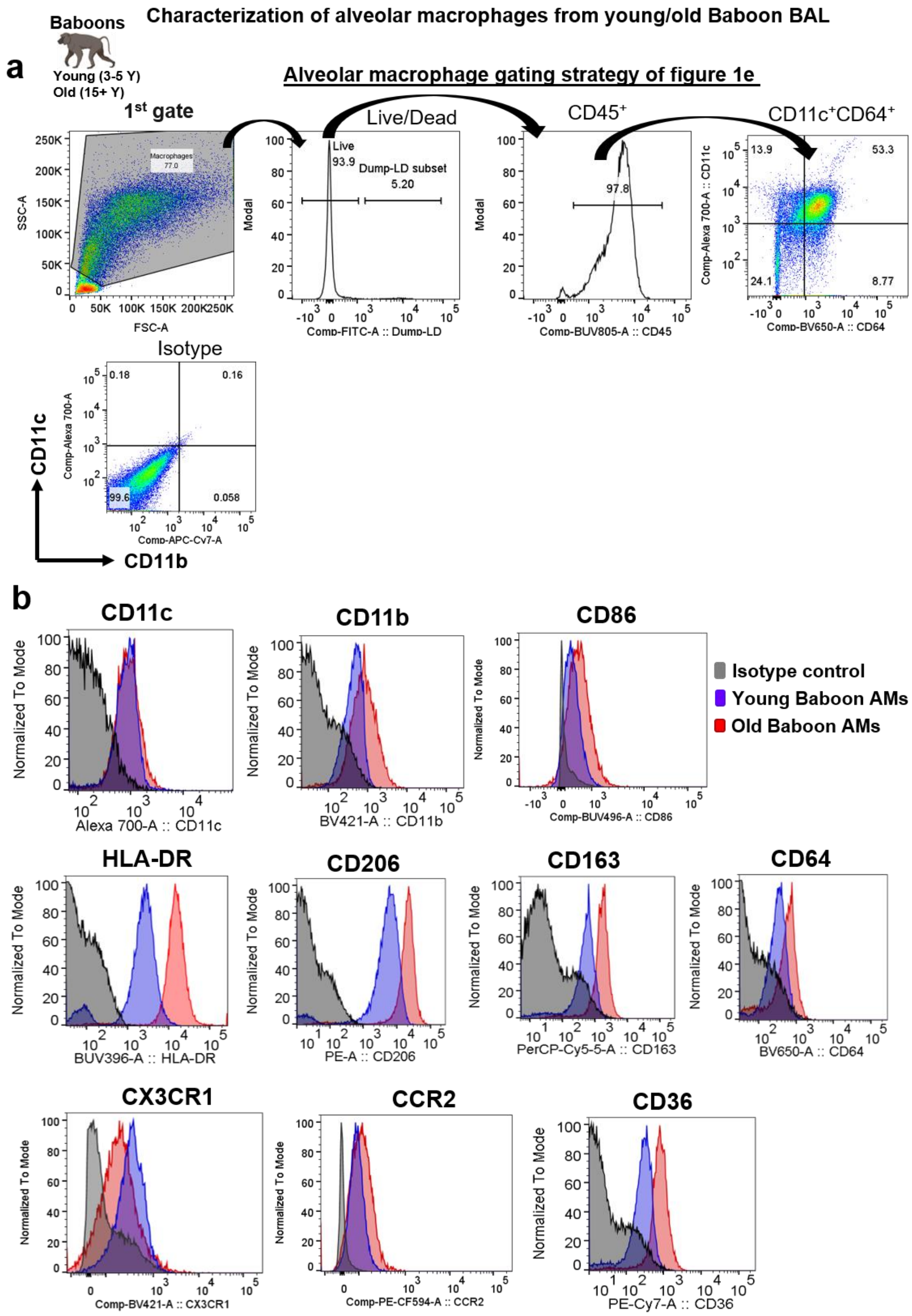

Figure S4. Gating strategy for characterizing bAMs from young and old baboons. The initial gate was to identify the macrophage populations from BAL. Next, dead cells were excluded to select only live cell populations. Then $CD45^{+}$ cells were identified and gated based on CD64 versus CD11c expression. The gating was determined using isotype control as a reference. Subsequently, the $CD64^{+}CD11c^{+}$ populations were further identified with additional markers, including CD11c, CD11b, CD86, HLA-DR, CD206, CD163, CD64, CX3CR1, CCR2, and CD36. The data are representative of 4 young and 4 old baboons. The baboon cartoon schematic was created in BioRender. Pahari, S. (2025) https://BioRender.com/x07c111.

Figure S5

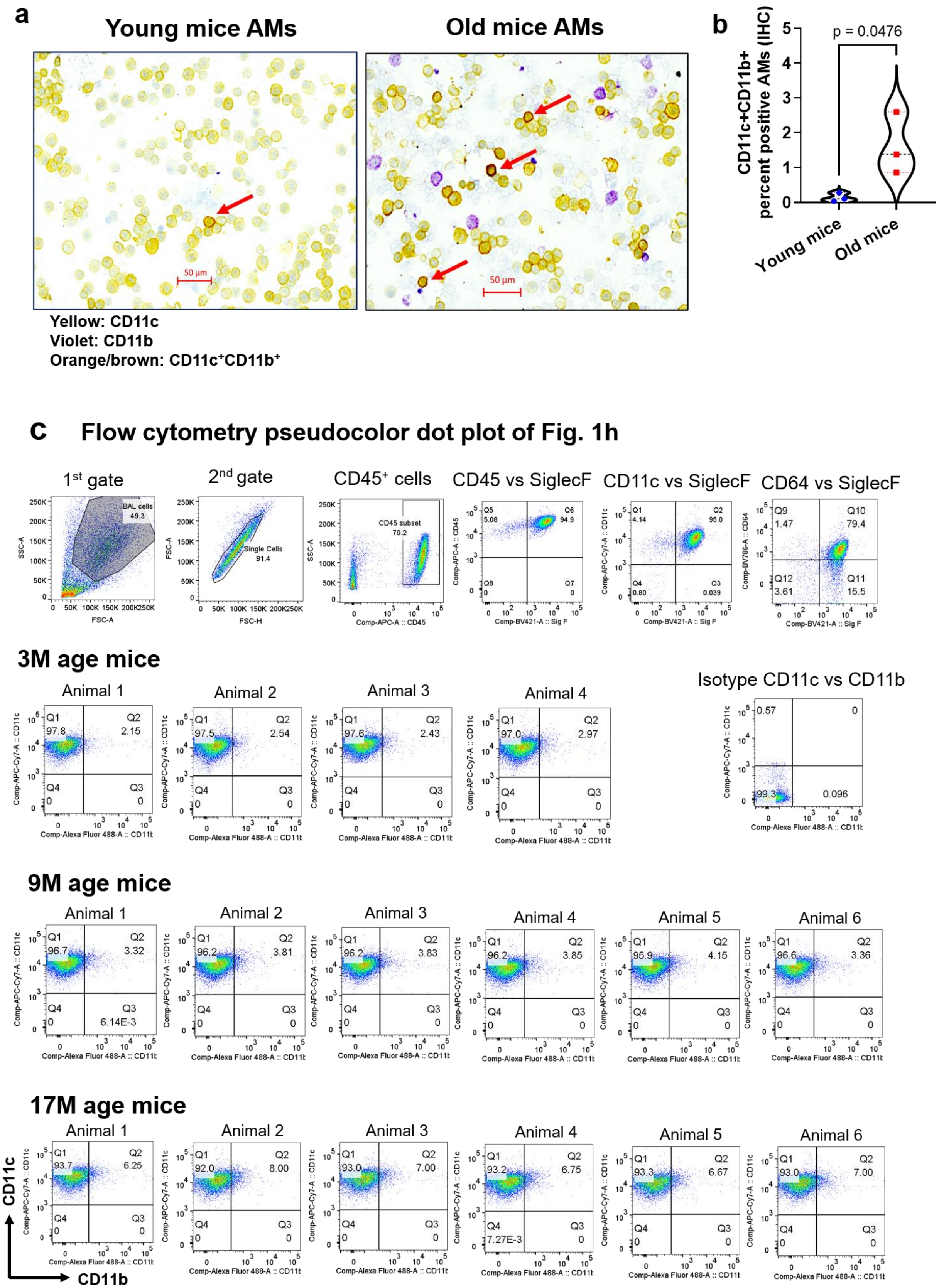
a
Young mice AMs
Old mice AMs
50 µm
Yellow: CD11c
Violet: CD11b
Orange/brown: CD11c+CD11b+
b
p = 0.0476
CD11c+CD11b+ percent positive AMs (IHC)
Young mice
Old mice
c Flow cytometry pseudocolor dot plot of Fig. 1h
1st gate
2nd gate
CD45+ cells
CD45 vs SiglecF
CD11c vs SiglecF
CD64 vs SiglecF
BAL cells 49.3
Single Cells 91.4
CD45 subset 70.2
3M age mice
Animal 1
Animal 2
Animal 3
Animal 4
Isotype CD11c vs CD11b
9M age mice
Animal 5
Animal 6
17M age mice
CD11c
CD11b

Figure S5. Identification of CD11c and CD11b positive cells by IHC in mAMs from young and old mice. mAMs were isolated by BAL. Cells from individual mice were cytospun onto slides and fixed with methanol. (a) Immunohistochemistry was performed using chromogenic anti-mouse CD11c (yellow) and CD11b (violet) antibodies. Red arrows indicate $CD11c^{+}CD11b^{+}$ cells in AMs from old mice (orange/brown). (b) Violin plot showing pooled frequencies of $CD11c^{+}CD11b^{+}$ cells from 3 mice per group. Data are presented as mean ± SEM and were analyzed using an unpaired two-sided Student's t-test. Each dot represents one individual mouse (N=3). (c) Flow cytometry pseudocolor dot plot of Figure 1h showing gating strategy and $CD11c^{+}CD11b^{+}$ percent population (quadrant Q2 gate) in 3M-, 9M- and 17M-old mice.

Figure S6

Flow cytometry pseudocolor dot plot of Fig. 1h

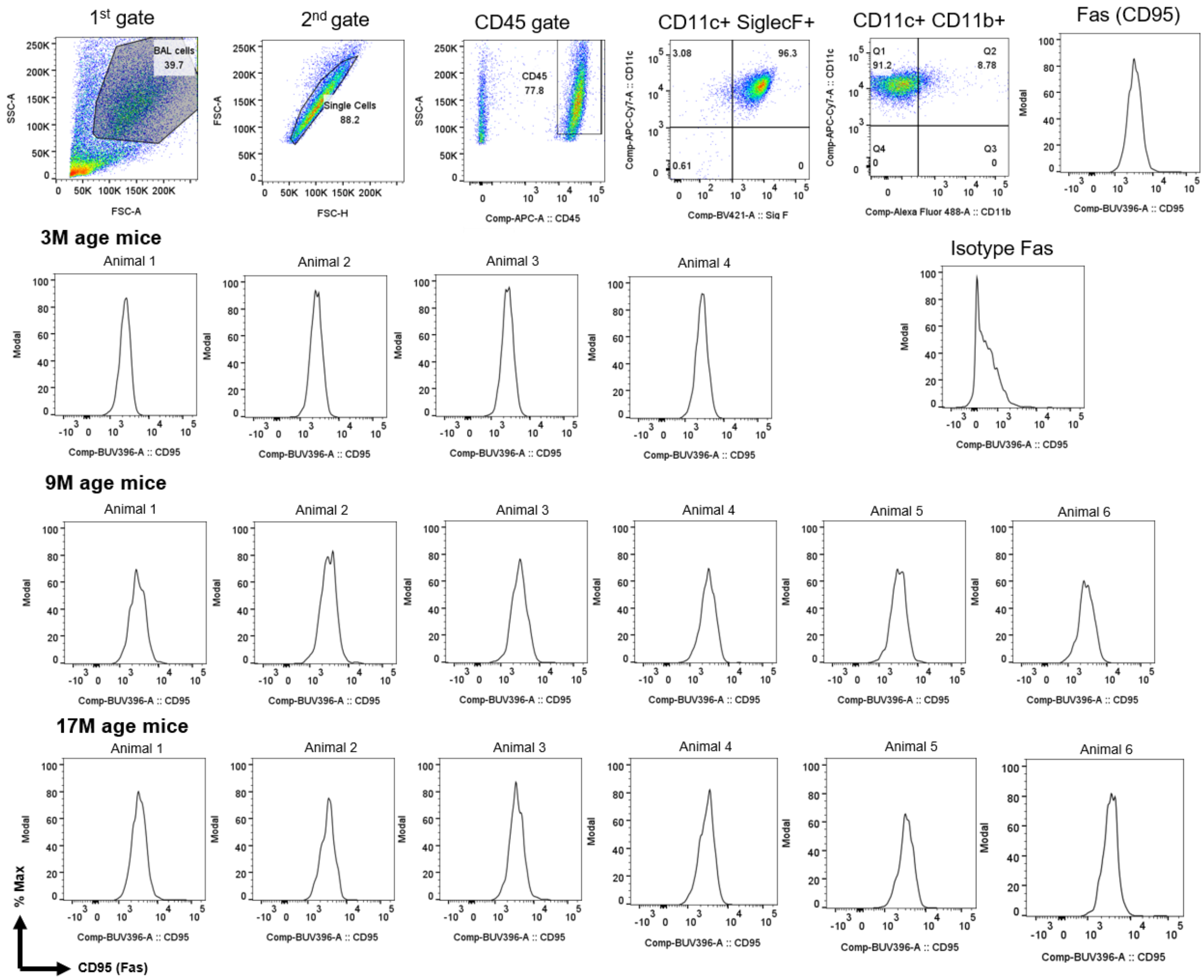

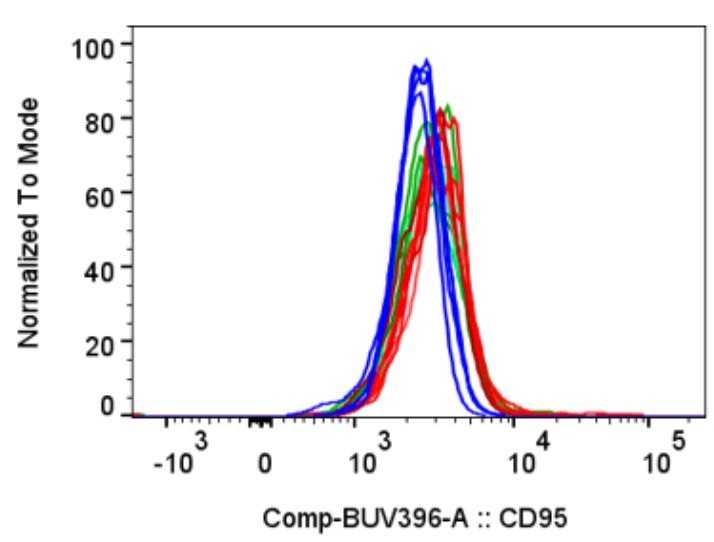

Sample Name	Subset Name	Geometric Mean : Comp-BUV396-A
BAL_3M 1.fcs	Q2: CD11b+ , CD11c+	2277
BAL_3M 2.fcs	Q2: CD11b+ , CD11c+	2441
BAL_3M 3.fcs	Q2: CD11b+ , CD11c+	2487
BAL_3M 4.fcs	Q6: CD11b+ , CD11c+	2285
BAL_17M 1.fcs	Q2: CD11b+ , CD11c+	3079
BAL_17M 2.fcs	Q2: CD11b+ , CD11c+	3009
BAL_17M 3.fcs	Q2: CD11b+ , CD11c+	3097
BAL_17M 4.fcs	Q2: CD11b+ , CD11c+	2734
BAL_17M 5.fcs	Q2: CD11b+ , CD11c+	3095
BAL_17M 6.fcs	Q2: CD11b+ , CD11c+	3175
BAL_9M 1.fcs	Q2: CD11b+ , CD11c+	2748
BAL_9M 2.fcs	Q2: CD11b+ , CD11c+	2823
BAL_9M 3.fcs	Q2: CD11b+ , CD11c+	2803
BAL_9M 4.fcs	Q2: CD11b+ , CD11c+	2717
BAL_9M 5.fcs	Q2: CD11b+ , CD11c+	2983
BAL_9M 6.fcs	Q2: CD11b+ , CD11c+	2781

Figure S6. Flow cytometry pseudocolor dot plots and histogram plots corresponding to Figure 1h. Data showing Fas (CD95) expression within pre-gated CD45$^+$SiglecF$^+$CD11c$^+$CD11b$^+$ populations. Data points represent individual MFI values from 3-month-old and 17-month-old mice. Individual mouse data are shown. Please see the corresponding main figure for additional details.

Figure S7

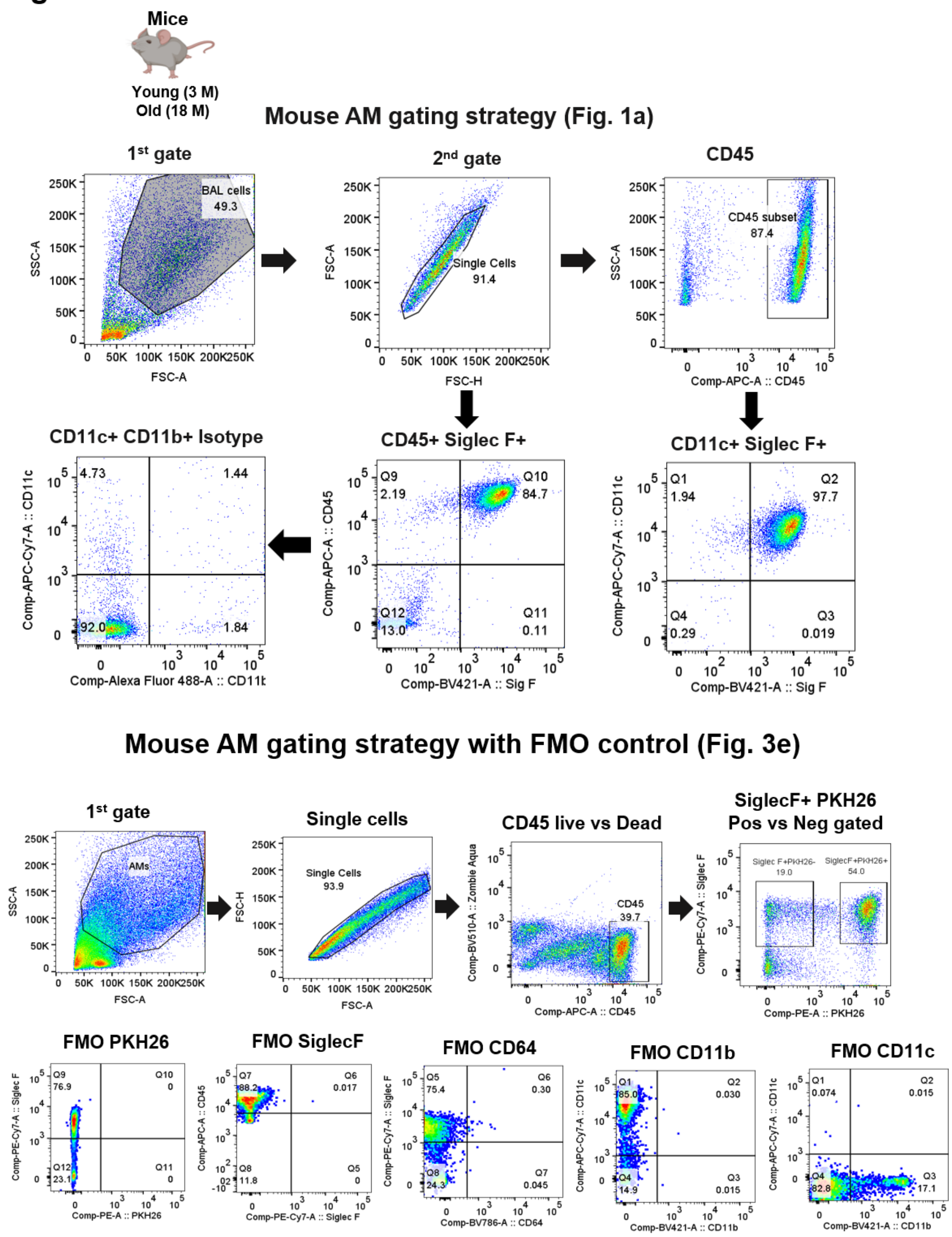

Figure S7. Gating strategy of Figure 1a and Figure 3e. In figure 1a, the initial gate was to select the macrophage populations from mouse BAL. Next, single cells were selected. Then $CD45^+$ cells were identified and gated based on either SiglecF versus CD11c expression or CD45 versus SiglecF double positive populations. Further, gating was performed to identify $CD11c^+$ $CD11b^+$ populations. Gating was determined using isotype control as a reference. In figure 3e, the initial gate was to identify the macrophage populations (AMs). The 2nd gate shows single cell selection. The next gate selects $CD45^+$ live cells from a zombie Aqua dead cell population which were then sorted by SiglecF versus $PKH26^+$ and $PKH26^-$ populations. PKH26, SiglecF, CD64, CD11b, CD11c FMO controls were used to select the specific gated populations. The mice cartoon schematic was created in BioRender. Pahari, S. (2025) https://BioRender.com/x07c111.

Figure S8

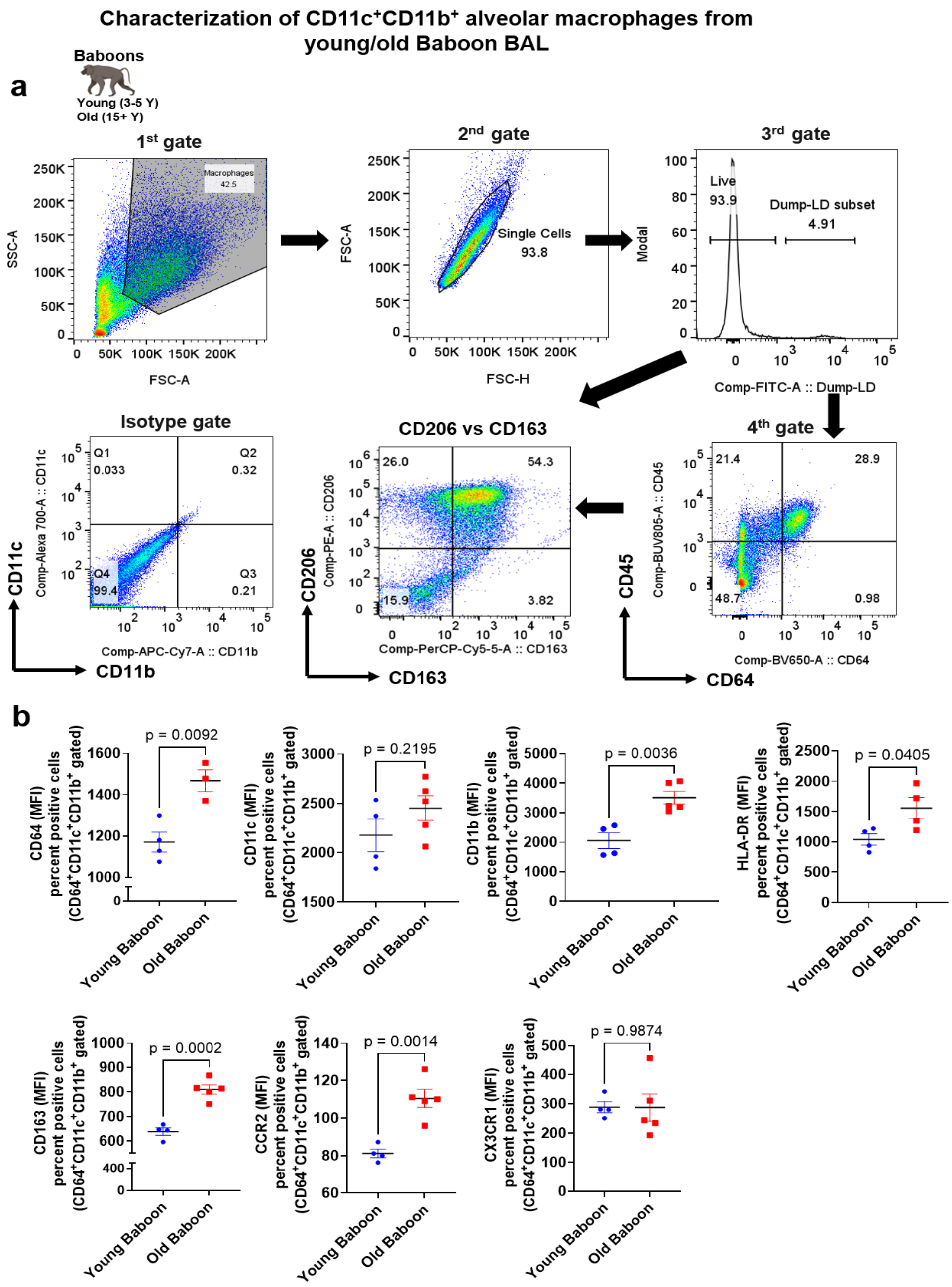

Figure S8. Characterization of $CD11c^{+}CD11b^{+}$ bAMs from young and old baboons. Isolated bAMs were gated on the macrophage populations from BAL (1st gate). Single cells were then separated by gating (2nd gate). Next, dead cells were excluded to select only live cell populations (3rd gate). Then AM populations were separated by gating on $CD45^{+}CD64^{+}$ double-positive populations (4th gate), followed by CD206 vs CD163 gating. The gating was determined using isotype control as a reference. Subsequently, the $CD11c^{+}CD11b^{+}$ double-positive populations were further gated with additional markers, including CD64, CD11c, CD11b, HLA-DR, CD163, CCR2, and CX3CR1. Each dot indicates individual donors. Data are presented as the mean ± SEM from 4 young and 5 old baboons. Statistical analysis was performed using an unpaired, two-sided Student's t-test. The baboon cartoon schematic was created in BioRender. Pahari, S. (2025) https://BioRender.com/x07c111.

Figure S9

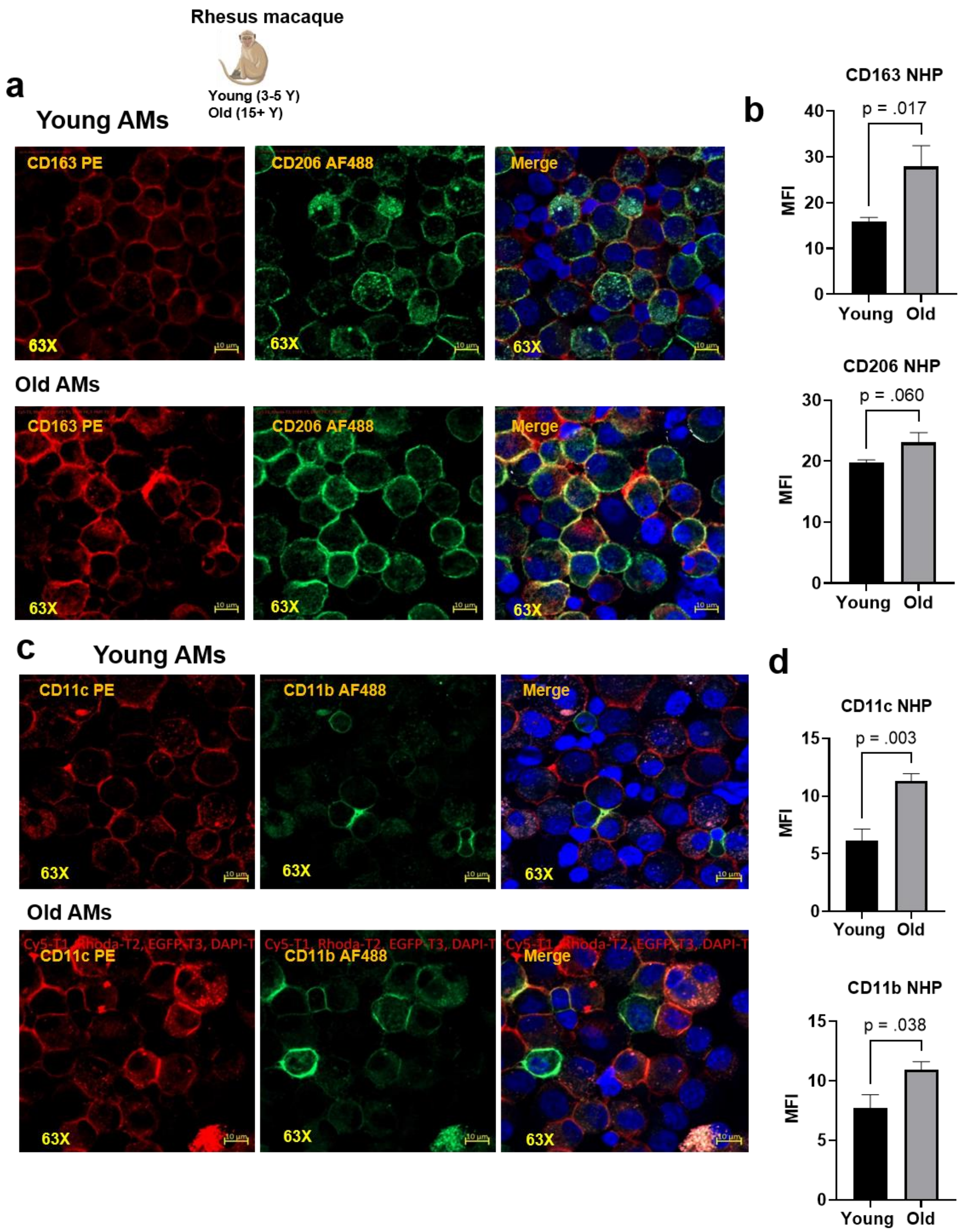

Figure S9. Phenotypic and functional characterization of old Rhesus macaque AMs (rAMs) compared to young rAMs. (a-d) RAMs were isolated from BAL samples. Confocal microscopy data show the expression of CD163 and CD206 cell surface receptors in panels (a) and (b). Panels (c) and (d) show the expression of CD11c and CD11b in positive populations of old rAMs compared to those from young rAMs. Anti-human/NHP CD163 PE and CD11c PE are shown in red, while green indicates anti-human/NHP CD206 and CD11b Alexa Fluor 488 antibodies. DAPI is used for nuclear staining, appearing blue. The scale bar represents 10 µm, with images at 63× magnification. (b) and (d) The bar graph displays cumulative data from two young (N=2) and two old donors (N=2). The confocal data were quantified as MFI and presented as bar graphs. Data from representative experiments are shown as mean ± SEM and analyzed using an unpaired, two-sided Student's t-test. The rhesus macaque cartoon schematic was created in BioRender. Pahari, S. (2025) https://BioRender.com/x07c111.

Figure S10

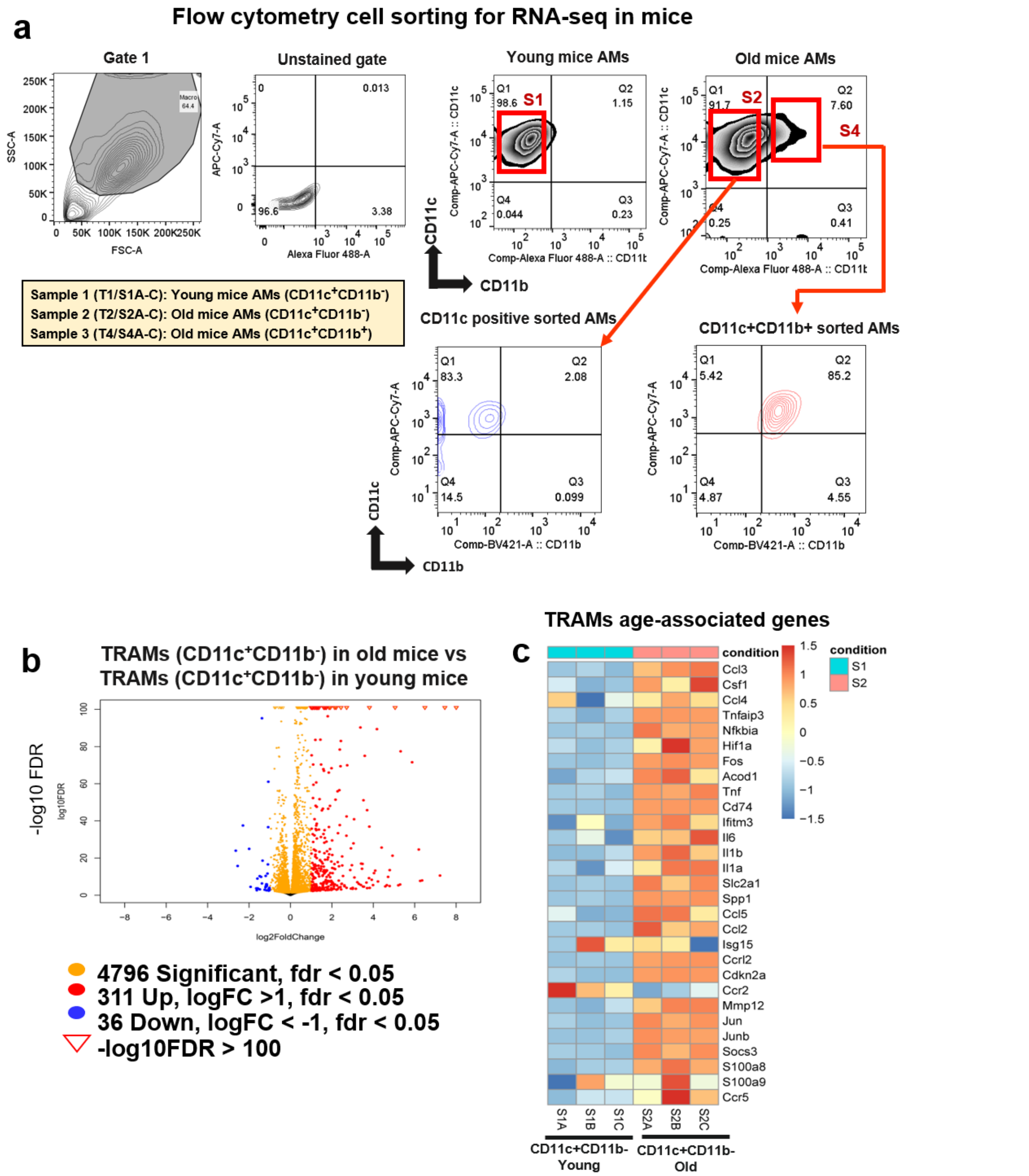

Figure S10. Transcriptional profile in flow cytometry-sorted young and old mAM subpopulations. The figure presents a transcriptomic analysis using bulk-RNA sequencing (RNA-seq) on four flow cytometry-sorted populations (**Figure 2**). S1: resident AMs from young mice (CD11c^{+} CD11b^{-}), S2: resident AMs from old mice (CD11c^{+}CD11b^{-}), S4: immigrated AM population (CD11c^{+}CD11b^{+}). (a) Flow cytometry cell sorting for bulk-RNA-seq in mAM subpopulations pre- and post-sorting are shown for S2

and S4 populations. (b) The volcano plot compares resident mAMs ($CD11c^+$ $CD11b^-$) in old *versus* young mice. The false discovery rate (FDR) in relation to the magnitude of gene expression changes across a total of 14,509 genes. The analysis indicates that 311 out of 4,796 significant genes are significantly upregulated (red), with logFC >1 and FDR <0.05, and 36 genes are downregulated (blue) in the CD11c+CD11b- AM subpopulations, with logFC < -1 and FDR < 0.05. (c) The heatmaps show relative expression levels for the senescence- and age-associated gene set comparing mTRAMs ($CD11c^+$ $CD11b^-$) in old versus young mice.

Figure S11

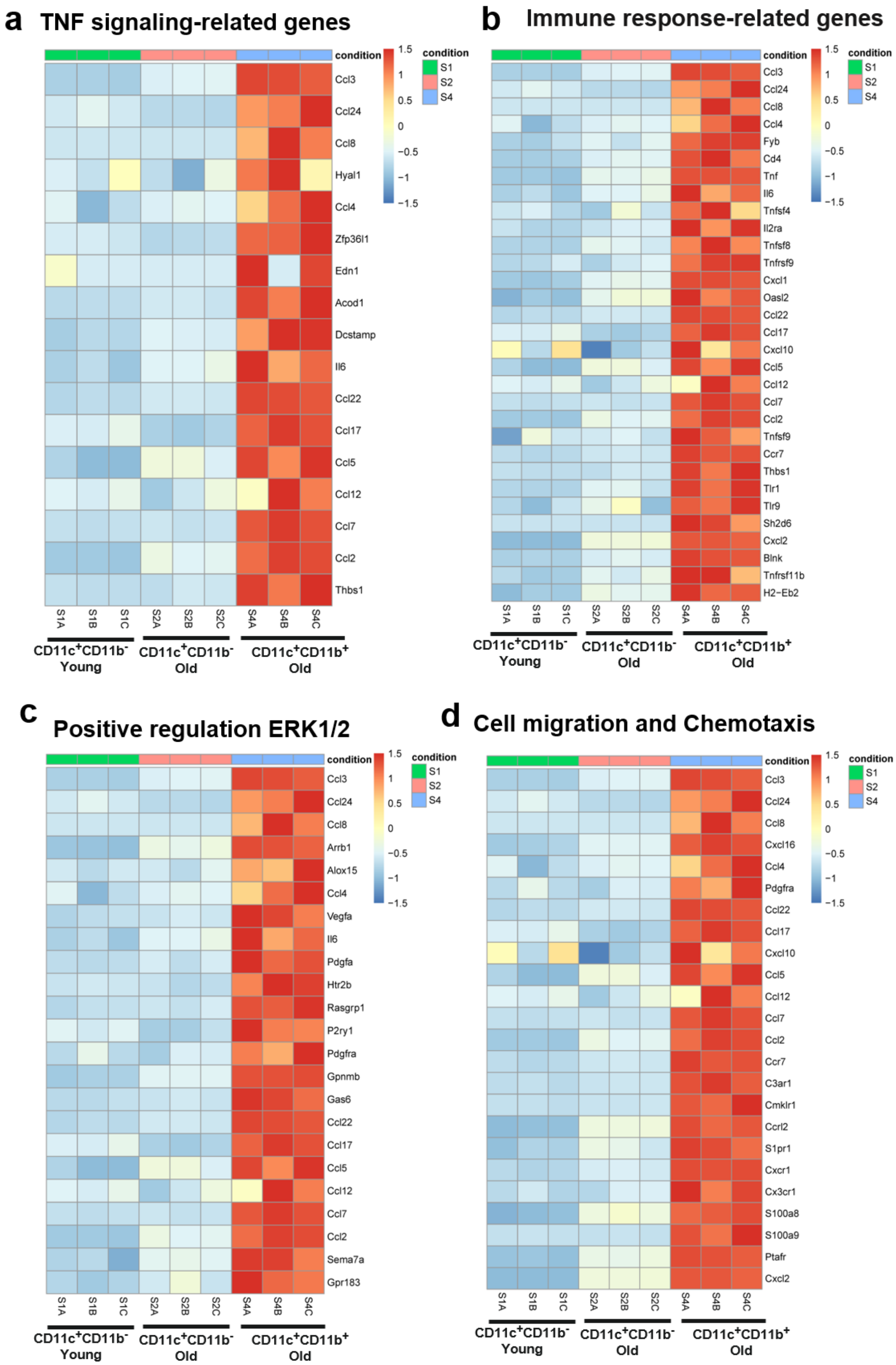

a TNF signaling-related genes
b Immune response-related genes
c Positive regulation ERK1/2
d Cell migration and Chemotaxis
condition
S1
S2
S4
1.5
1
0.5
0
−0.5
−1
−1.5
Ccl3
Ccl24
Ccl8
Hyal1
Ccl4
Zfp36l1
Edn1
Acod1
Dcstamp
Il6
Ccl22
Ccl17
Ccl5
Ccl12
Ccl7
Ccl2
Thbs1
Fyb
Cd4
Tnf
Tnfsf4
Il2ra
Tnfsf8
Tnfrsf9
Cxcl1
Oasl2
Cxcl10
Tnfsf9
Ccr7
Tlr1
Tlr9
Sh2d6
Cxcl2
Blnk
Tnfrsf11b
H2−Eb2
Arrb1
Alox15
Vegfa
Pdgfa
Htr2b
Rasgrp1
P2ry1
Pdgfra
Gpnmb
Gas6
Sema7a
Gpr183
Cxcl16
C3ar1
Cmklr1
Ccrl2
S1pr1
Cxcr1
Cx3cr1
S100a8
S100a9
Ptafr
S1A
S1B
S1C
S2A
S2B
S2C
S4A
S4B
S4C
CD11c+CD11b- Young
CD11c+CD11b- Old
CD11c+CD11b+ Old

Figure S11. Transcriptional profile in flow cytometry-sorted young and old mAM subpopulations. Part of Figure 2. The heatmap shows relative expression levels for (a) TNF signaling -related genes, (b) immune response-related genes, (c) positive regulation of ERK1/2 signaling-related genes, (d) cell migration and chemotaxis-related genes in mAMs from: the resident subpopulation from old mice ($CD11c^+CD11b^-$), immigrated mAMs ($CD11c^+CD11b^+$) and resident $CD11C^+CD11b^-$ subpopulation from young mice. The red arrow highlights certain highly upregulated genes in the $CD11c^+CD11b^+$ mAM subpopulation from old mice, indicating a distinct genetic profile (in red) compared to $CD11c^+$ $CD11b^-$ AMs from either young or old mice. Blue represents low expression, while red represents higher expression.

Figure S12

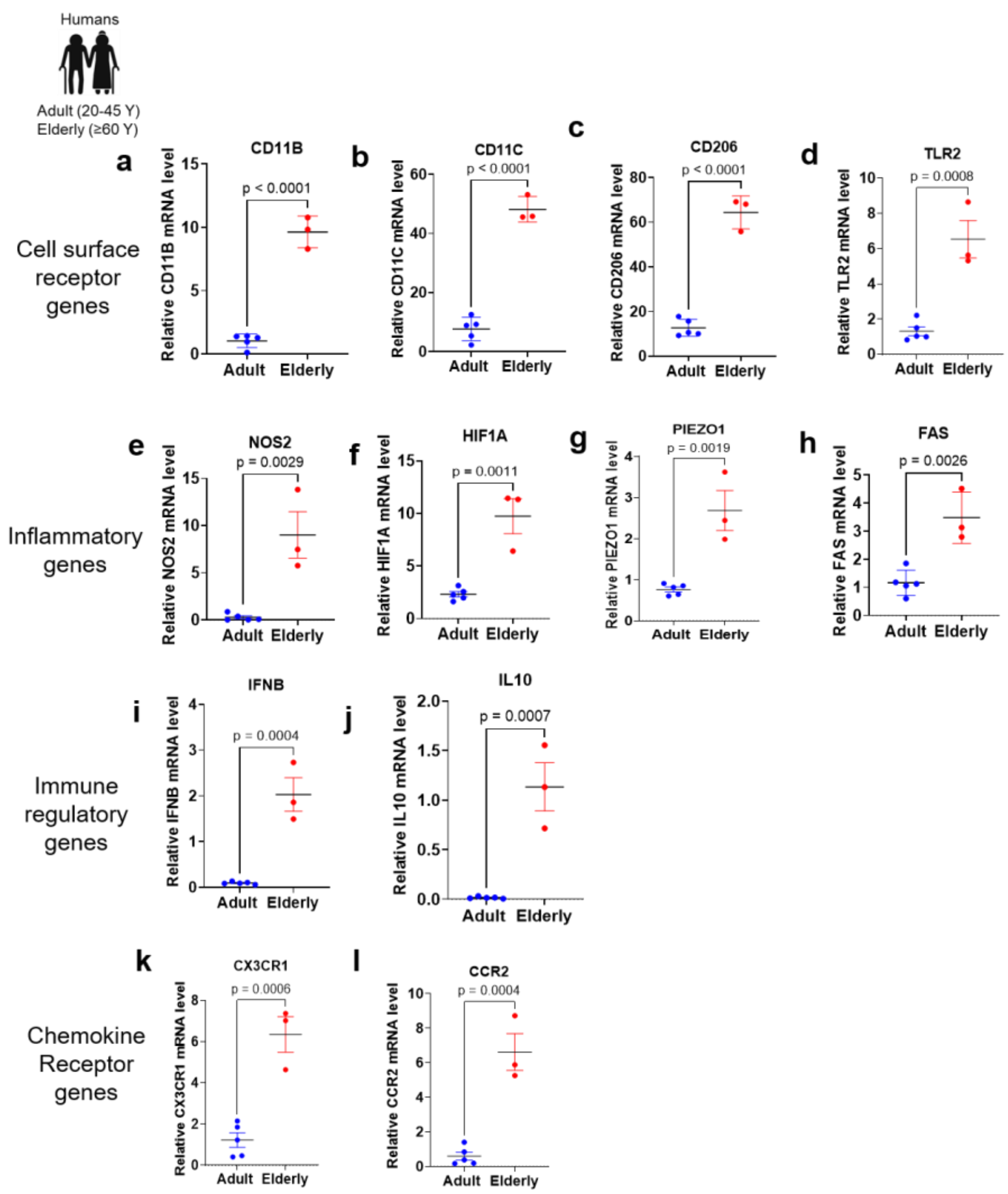

Figure S12. Analysis of hAM gene expression. Part of Figure 2. RNA was isolated from hAMs immediately after BAL without any culturing. The expression of various genes was analyzed: Cell Surface Receptors: CD11B, CD11C, CD206, and TLR2 (Panels a-d); Inflammatory Genes: NOS2, HIF1A, PIEZO1, and FAS (Panels e-h); Immune Regulatory Genes: IFNB and IL10 (Panels i-j) and Chemokine Receptors: CX3CR1 and CCR2 (Panels k-l). Gene expression was determined using qRT-PCR. Cycle threshold (Ct) values were normalized to ACTB (β-actin), and mRNA levels were assessed relative to hAMs from adult individuals. Data are presented as the mean ± SEM from 5 adult (N=5) and 3 elderly donors (N=3). Statistical analysis was performed using an unpaired, two-sided Student's t-test. The human cartoon schematic was created in BioRender. Pahari, S. (2025) https://BioRender.com/x07c111.

Figure S13

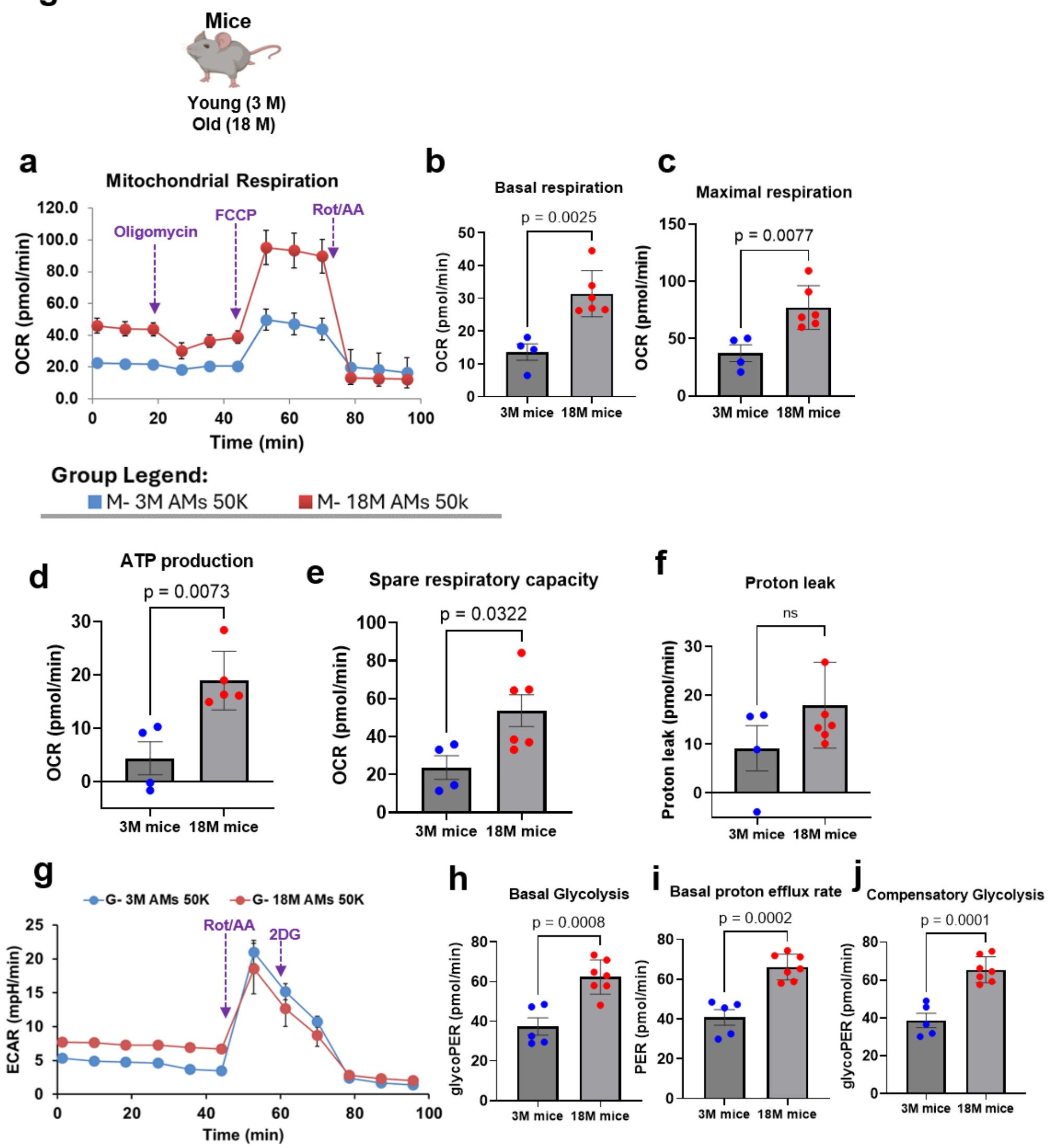

Figure S13. Metabolic and oxidative changes in mAMs from old mice. Part of Figure 3. Extracellular flux analysis was performed in freshly isolated AMs from young and old mice by Seahorse analyzer. The oxygen consumption rate (OCR) and extracellular acidification rate (ECAR) were analyzed under basal conditions and in response to Mito Stress Test reagent. (a) Kinetics analysis of mitochondrial respiration detected by OCR and ATP production. The dashed lines indicate when O: oligomycin; FCCP: Carbonyl cyanide-4 (trifluoromethoxy) phenylhydrazone; Rot/AA: rotenone and antimycin A were added. (b-f) Cumulative data from 5 young and 6 old mice showing Mito Stress Test bar graphs with higher basal respiration, maximal respiration, ATP production, and higher Spare respiratory capacity (SRC) and moderate proton leak in AMs from old mice compared to young mice AMs. Data are mean ± SEM, and analyzed by unpaired, two-sided Student's t-test. (g) Glycolytic rate (ECAR) kinetics graph demonstrates an increase in the glycolytic rate in AMs from old mice as compared to young mice AMs. Rot & AA were used to accelerate, and 2-Deoxy-D-glucose (2-DG) was used to inhibit glycolysis. (h-j) Bar graph shows the quantification of basal glycolysis, basal proton efflux, and compensatory glycolysis in young and old mice AMs. Cumulative data are shown for N = 5 young and 7 old mice, mean ± SEM, and analyzed by unpaired, two-sided Student's t-test. The mice cartoon schematic was created in BioRender. Pahari, S. (2025) https://BioRender.com/x07c111.

Figure S14

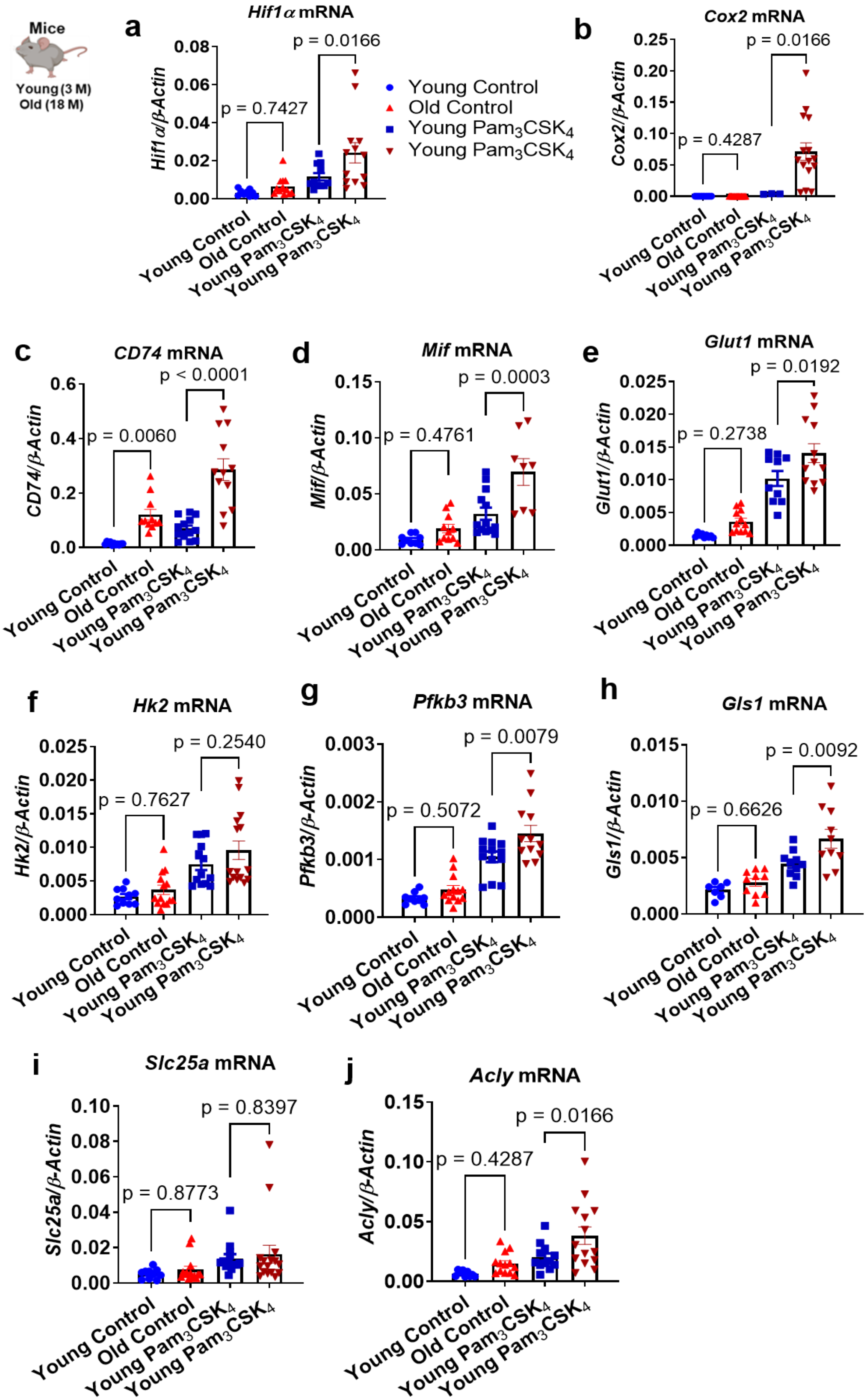
Mice
Young (3 M)
Old (18 M)
a
Hif1α mRNA
Hif1α/β-Actin
p = 0.7427
p = 0.0166
Young Control
Old Control
Young Pam3CSK4
Young Pam3CSK4
b
Cox2 mRNA
Cox2/β-Actin
p = 0.4287
p = 0.0166
c
CD74 mRNA
CD74/β-Actin
p = 0.0060
p < 0.0001
d
Mif mRNA
Mif/β-Actin
p = 0.4761
p = 0.0003
e
Glut1 mRNA
Glut1/β-Actin
p = 0.2738
p = 0.0192
f
Hk2 mRNA
Hk2/β-Actin
p = 0.7627
p = 0.2540
g
Pfkb3 mRNA
Pfkb3/β-Actin
p = 0.5072
p = 0.0079
h
Gls1 mRNA
Gls1/β-Actin
p = 0.6626
p = 0.0092
i
Slc25a mRNA
Slc25a/β-Actin
p = 0.8773
p = 0.8397
j
Acly mRNA
Acly/β-Actin
p = 0.4287
p = 0.0166

Figure S14. RNA expression levels of aerobic metabolism-related genes, including *Hif1α, Cox2, CD74, Mif, Glut1, Hk2, Pfkb3, Gls1, Slc25a,* and *Acly* in mAMs isolated from young and old mice. BAL cells were isolated from young (3 month) and old (18 month) mice and then cultured with Pam_3CSK_4 (100 ng/mL) for 24h. RNA was extracted, and expression of various genes was analyzed: (a) *Hif1α,* (b) *Cox2,* (c) *CD74,* (d) *Mif,* (e) *Glut1,* (f) *Hk2,* (g) *Pfkb3,* (h) *Gls1,* (i) *Slc25a,* and (j) *Acly* by qRT-PCR and normalized to β-actin. Data were pooled from 2-4 BAL isolations with 5-10 mice per isolation. AMs from each isolation were stimulated in triplicate with Pam_3CSK_4. Each dot indicates individual replicates. Data are expressed as mean ± SEM, analyzed by ordinary one-way ANOVA (two-sided) with Sidak's multiple comparisons test. The mice cartoon schematic was created in BioRender. Pahari, S. (2025) https://BioRender.com/x07c111.

Figure S15

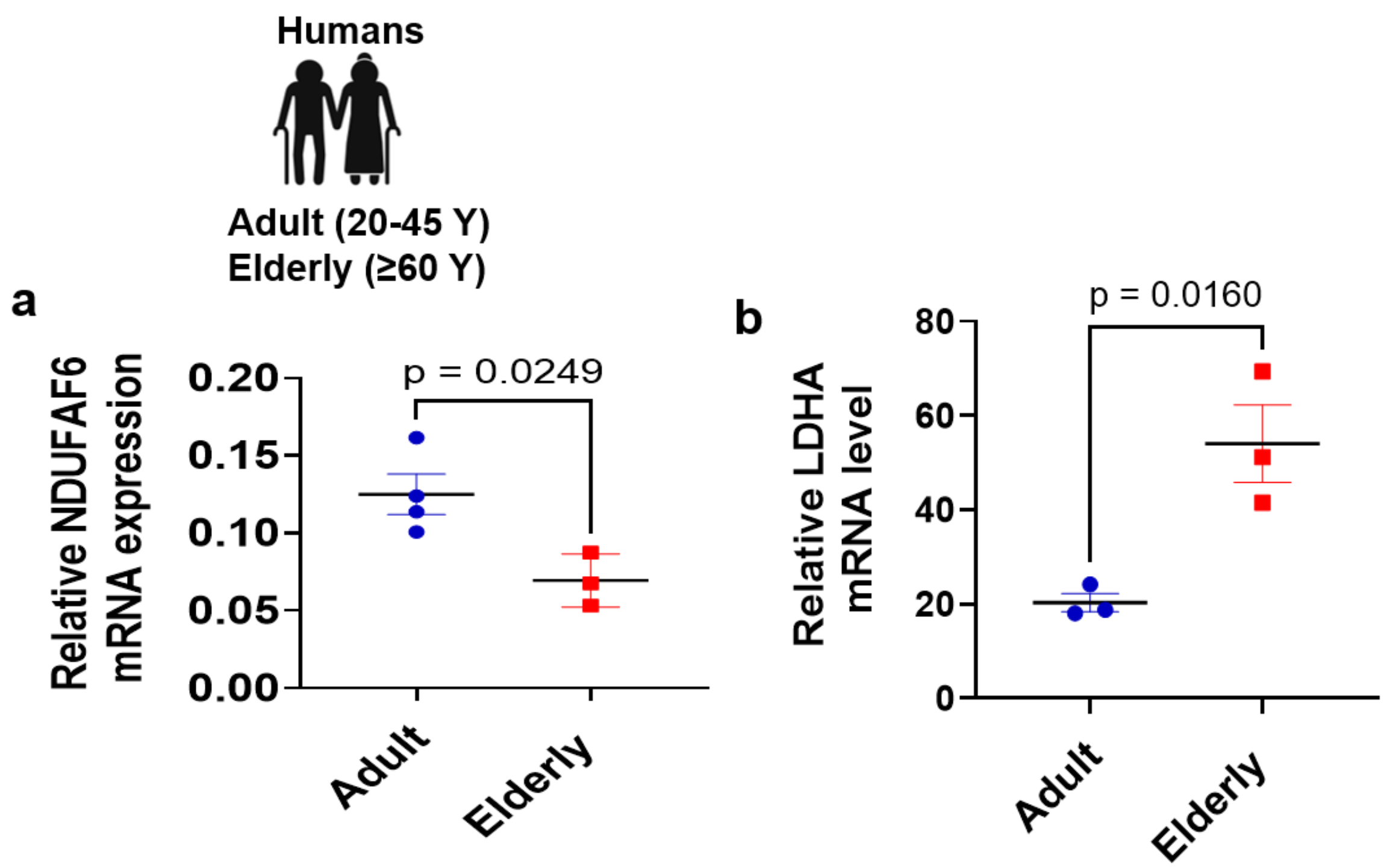

Figure S15. RNA expression levels of genes related to metabolic activity of hAMs isolated from adult or elderly humans. mRNA expression of human *NDUFAF6* (OXPHOS gene), *LDHA* (glycolytic gene) were assessed by qRT-PCR after normalizing with β-actin. Data are from N=4 adults and 3 elderly humans and are mean ± SEM, analyzed by unpaired, two-sided Student's t-test. The human cartoon schematic was created in BioRender. Pahari, S. (2025) https://BioRender.com/x07c111.

Figure S16

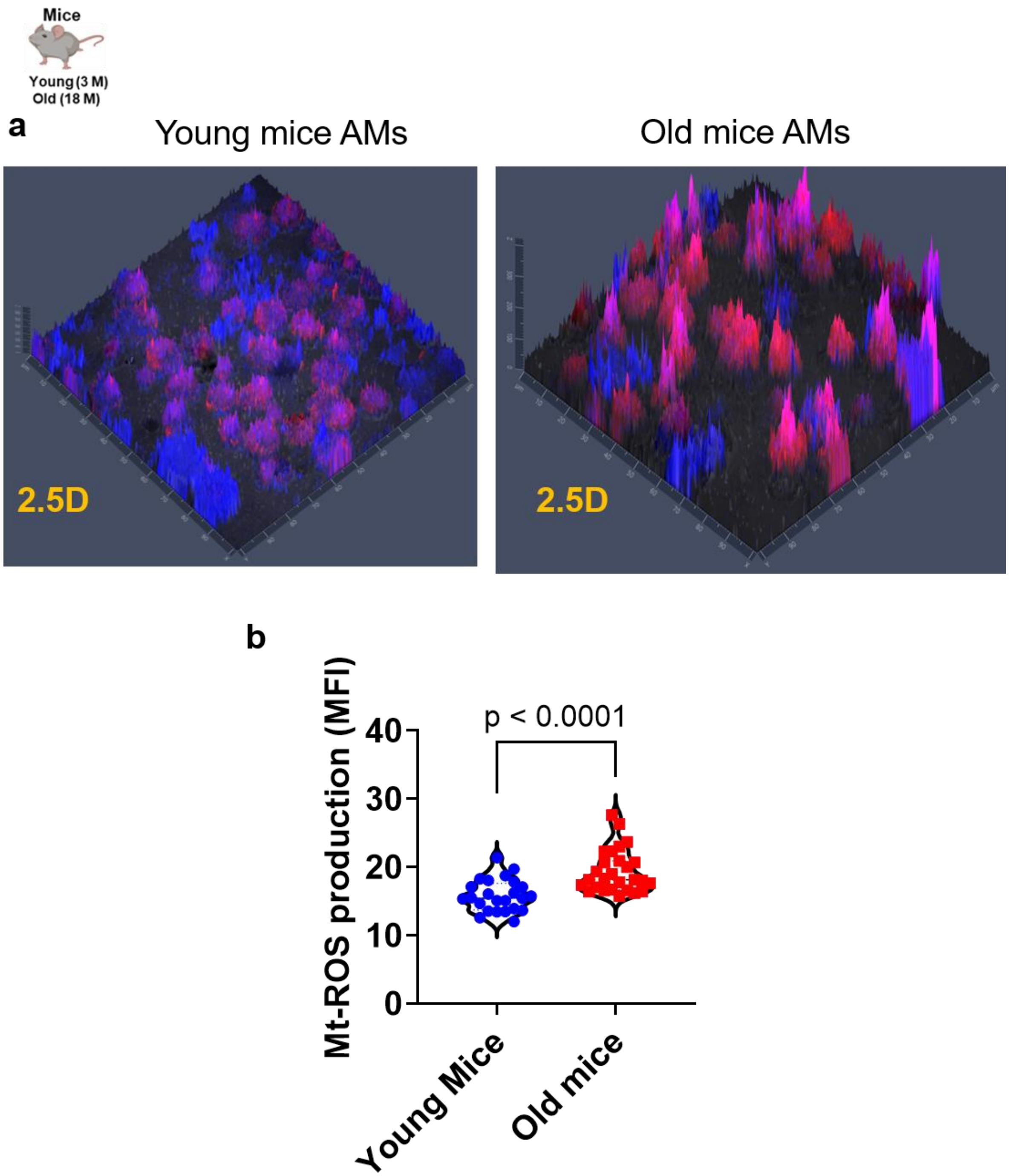

Figure S16. Mitochondrial ROS production in young and old mAMs. (a) AMs were isolated from young & old mice. Cells were stained with MitoSOX (5 μM) to demonstrate mitochondrial ROS by confocal microscopy. Magnification: 63×, scale bar: 5 μM. (b) The violin plot shows mitochondrial ROS represented as MFI (Arithmetic mean intensity). Cumulative data are shown for N = 3 mice/group with 6-8 microscopic fields with more than 25 AMs per field and analyzed by unpaired, two-sided Student's test. The mice cartoon schematic was created in BioRender. Pahari, S. (2025) https://BioRender.com/x07c111.

Figure S17

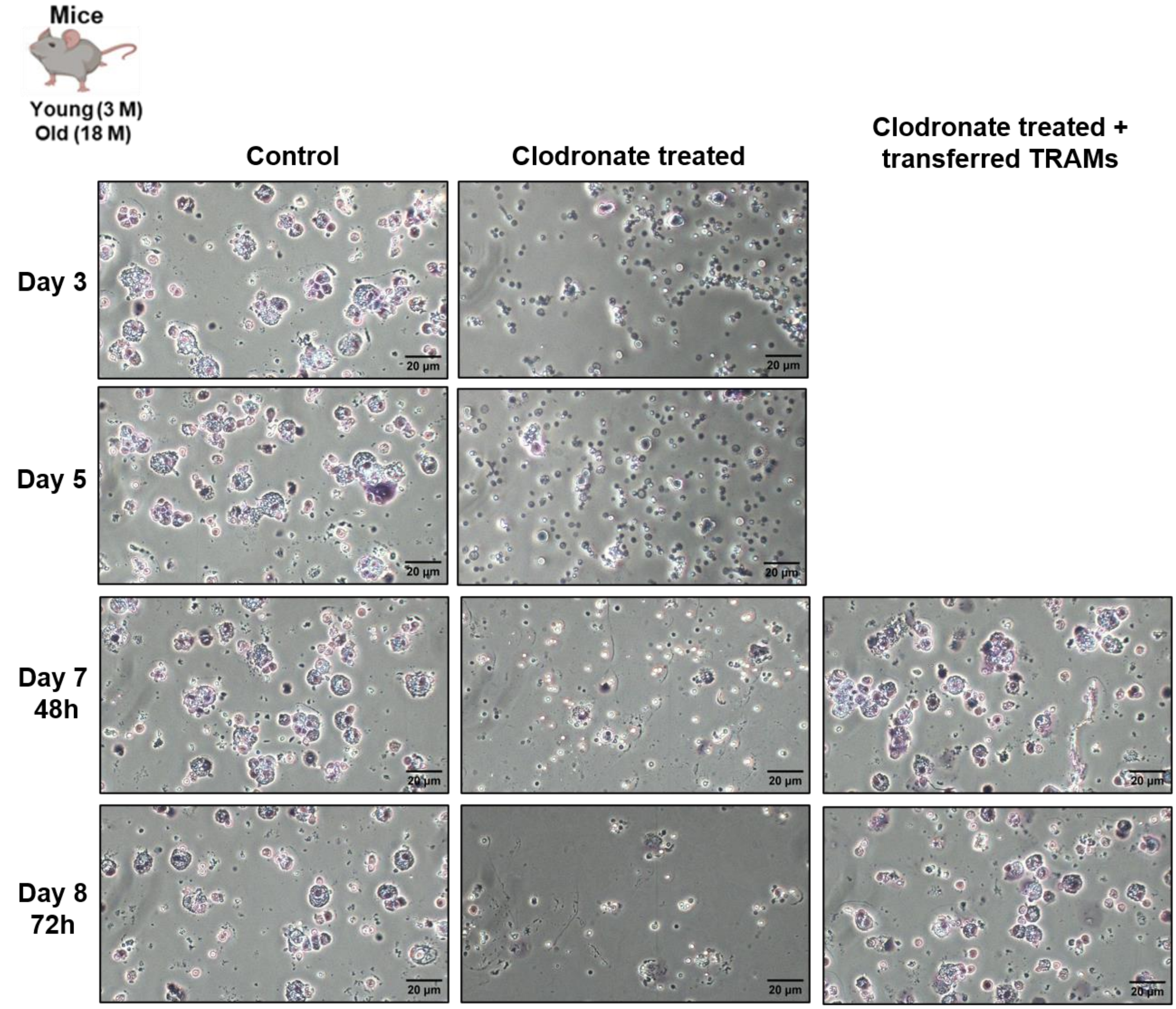

Figure S17. Morphological analysis of mAMs. Part of Figure 3. Young and old mice received two doses of clodronate liposomes (clodrolip.) administered i.t. on Day 0 and Day 3, effectively depleting resident mAMs from the alveolar compartments in young and old mice. On Day 5, CD11c^{+} BAL isolated resident AMs (donor cells) from young mice were adoptively transferred i.t. (using $1x10^{6}$ cells) into old recipient mice. On Day 7 and Day 8, AMs were isolated, and Wright-Giemsa staining was performed. The morphology of the AMs from the old mice was assessed after staining the cytospun cells and observing them using light microscopy. Scale bar 20µm and 40x magnification. The mice cartoon schematic was created in BioRender. Pahari, S. (2025) https://BioRender.com/x07c111.

Figure S18

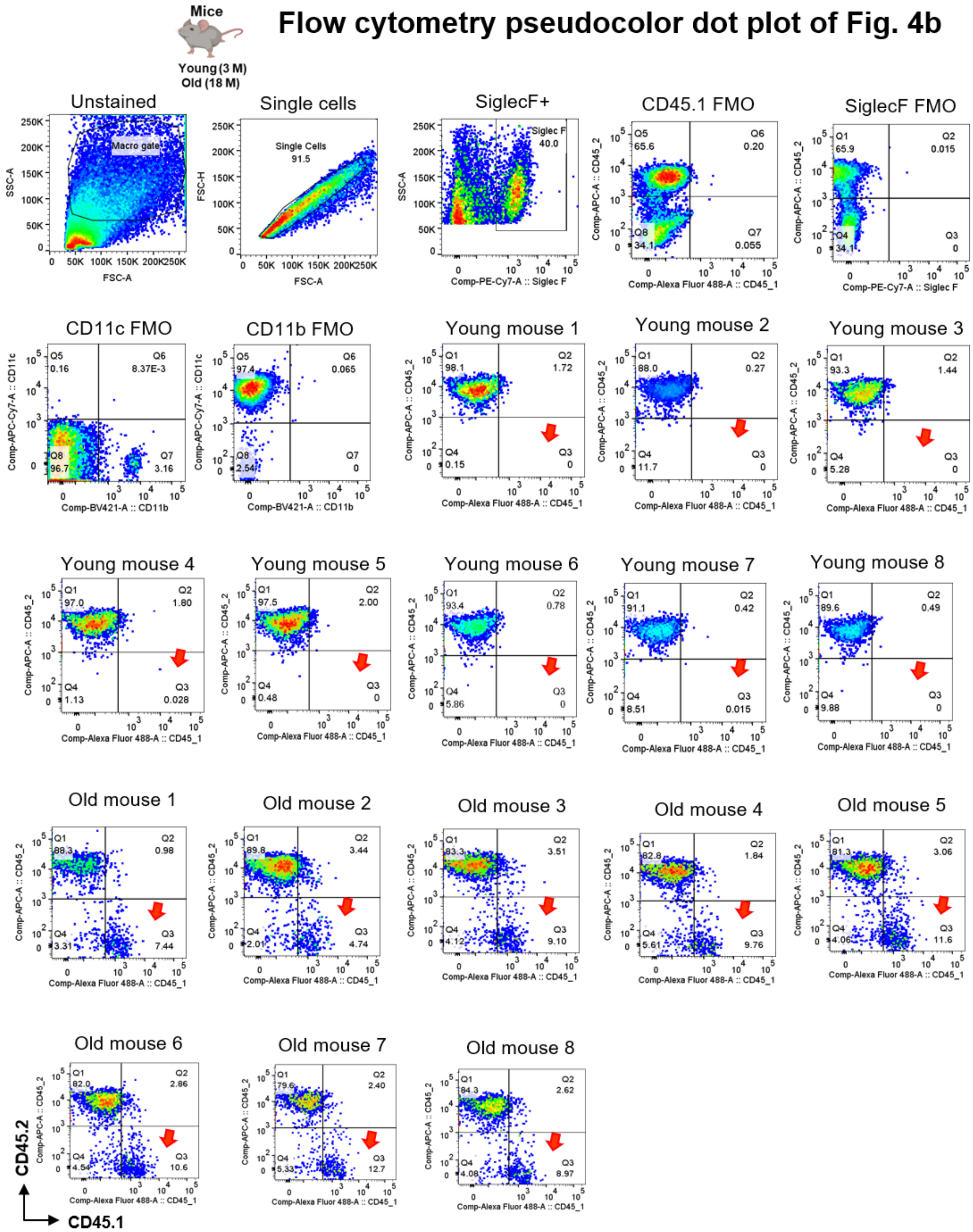

Figure S18. Flow cytometry pseudocolor dot plots corresponding to Figure 4b. Data showing the gating strategy and representative individual mouse data for CD45.2 versus CD45.1 expression. $CD45.1^{+}$ donor cells are identified in quadrant Q3. Red arrow indicates the population of interest. The mice cartoon schematic was created in BioRender. Pahari, S. (2025) https://BioRender.com/x07c111.

Figure S19

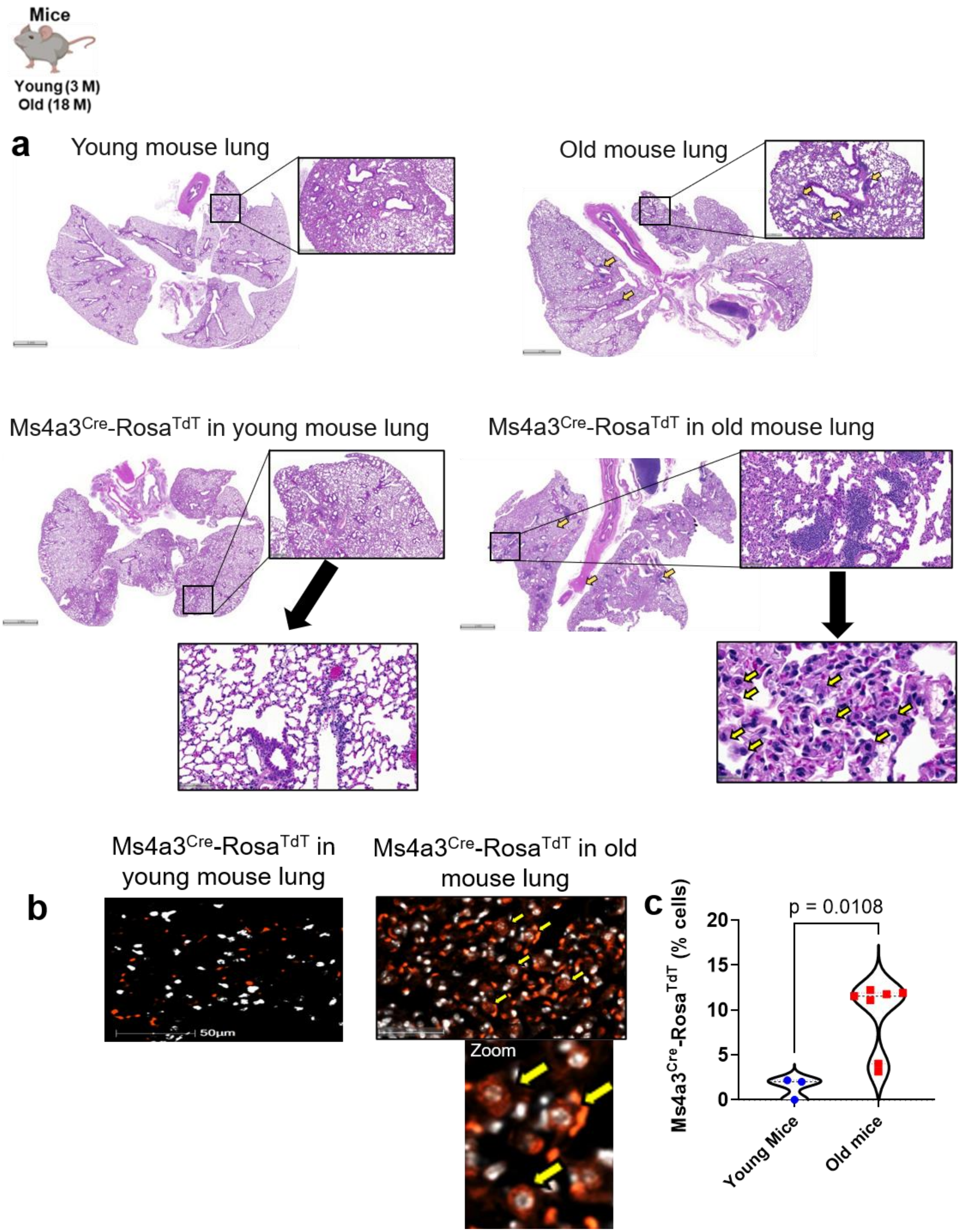

Mice
Young (3 M)
Old (18 M)
a
Young mouse lung
Old mouse lung
Ms4a3Cre-RosaTdT in young mouse lung
Ms4a3Cre-RosaTdT in old mouse lung
b
Ms4a3Cre-RosaTdT in young mouse lung
Ms4a3Cre-RosaTdT in old mouse lung
50μm
Zoom
c
p = 0.0108
Ms4a3Cre-RosaTdT (% cells)
20
15
10
5
0
Young Mice
Old mice

Figure S19. Histopathological Analysis of Ms4a3^{Cre}-Rosa^{TdT} MoAMs in the lungs of old mice. Ms4a3^{Cre}-Rosa^{TdT} bone marrow monocytes (donor cells) were adoptively transferred to young and old mice to track them and their progeny in the lungs of C57BL/6 mouse recipients. On the fifth day after the transfer, the entire lung lobes were collected from the recipient mice. The lung lobes were preserved in 10% formalin and underwent histopathological analysis following hematoxylin and eosin (H&E) staining. (a) The data demonstrate that the migrated MoAMs were present (indicated by blue hematoxylin staining) to a greater extent in the lungs of old mice and in old mice that received the Ms4a3^{Cre}-Rosa^{TdT} monocyte transfer. In contrast, the lungs of young mice or young mice receiving Ms4a3^{Cre}-Rosa^{TdT} showed limited cell infiltration in the alveolar compartments and lung parenchyma. The scale bar represents 2 mm, with images at 100× magnification. (b, c) Yellow arrows indicate the recruited Ms4a3^{Cre}-Rosa^{TdT}-positive MoAMs (red fluorescence). White dot indicates individual cells stained with DAPI (nucleus stain). Red fluorescence near DAPI (white) was counted and represented as a violin plot. The data are representative of three animals per group (N=3). The scale bar represents 50 µm, with images at 40× magnification. Data analyzed by unpaired, two-sided Student's test. The mice cartoon schematic was created in BioRender. Pahari, S. (2025) https://BioRender.com/x07c111.

Figure S20

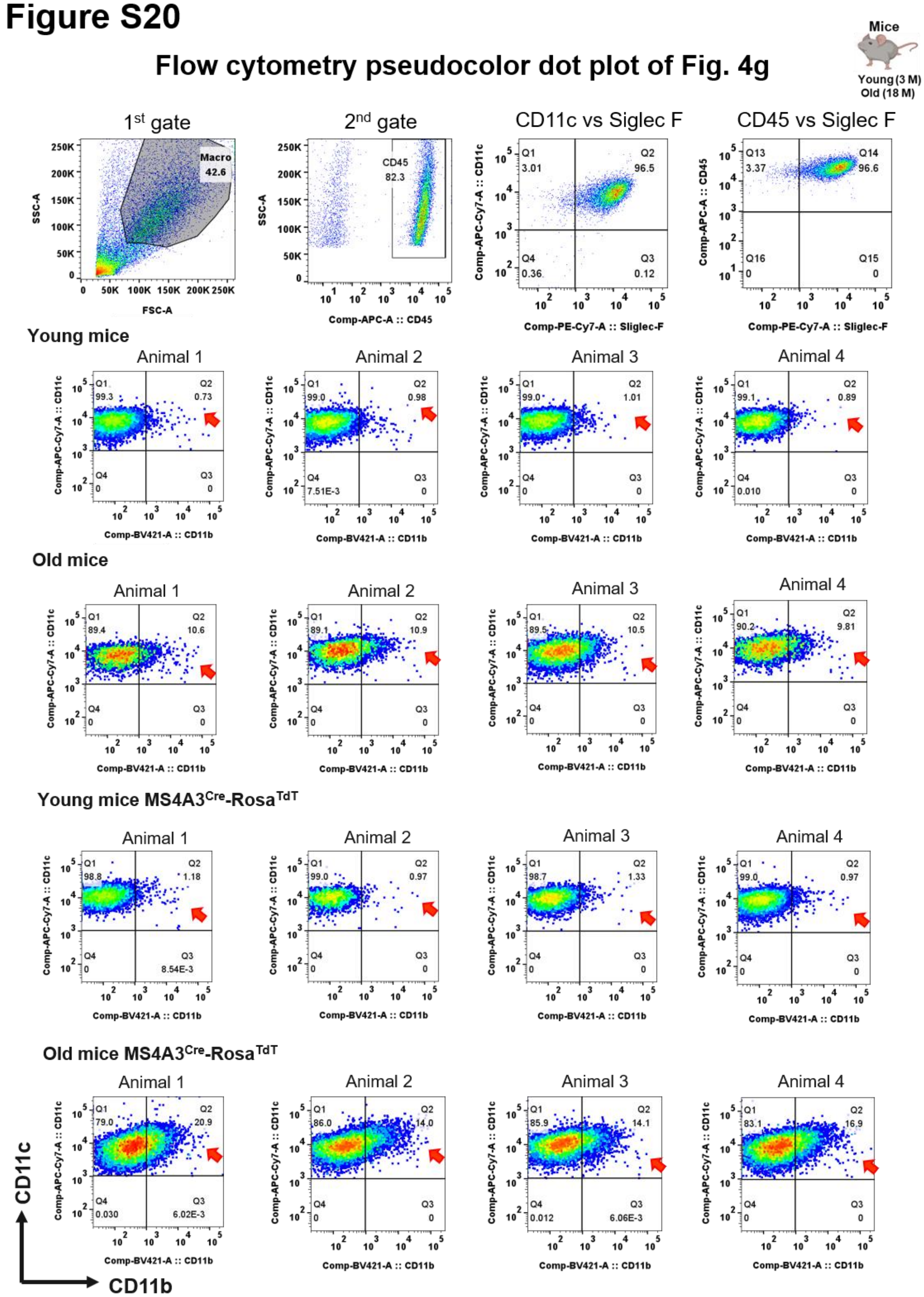

Figure S20. Flow cytometry pseudocolor dot plots corresponding to Figure 4g. Data showing the gating strategy and representative individual mouse data for $CD11c^{+}CD11b^{+}$ percentages on $CD45^{+}SiglecF^{+}$ gated populations. $CD11c^{+}CD11b^{+}$ cells are identified in quadrant Q2. Red arrow indicates the population of interest. N=4 mice/group. The mice cartoon schematic was created in BioRender. Pahari, S. (2025) https://BioRender.com/x07c111.

Figure S21

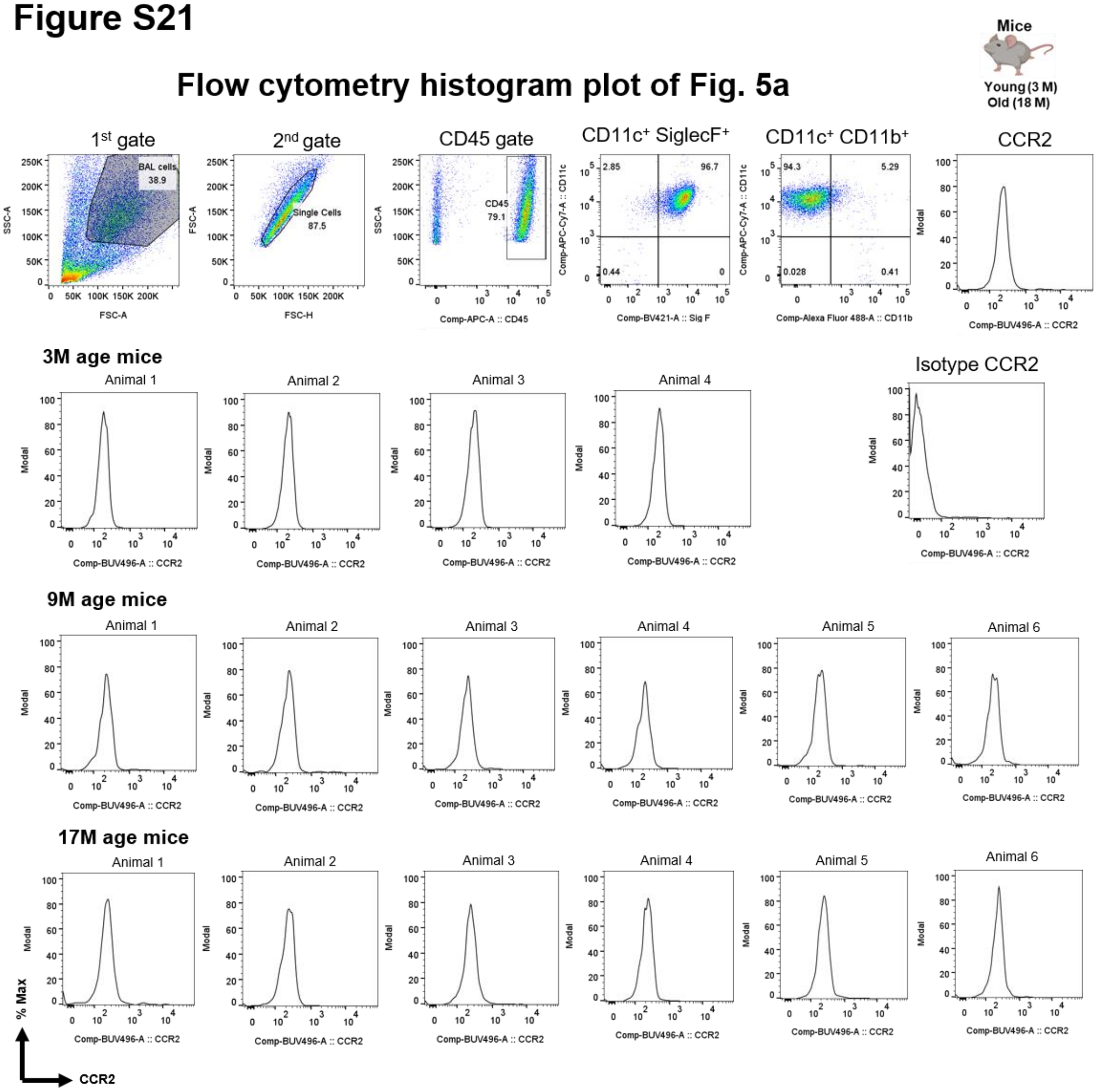

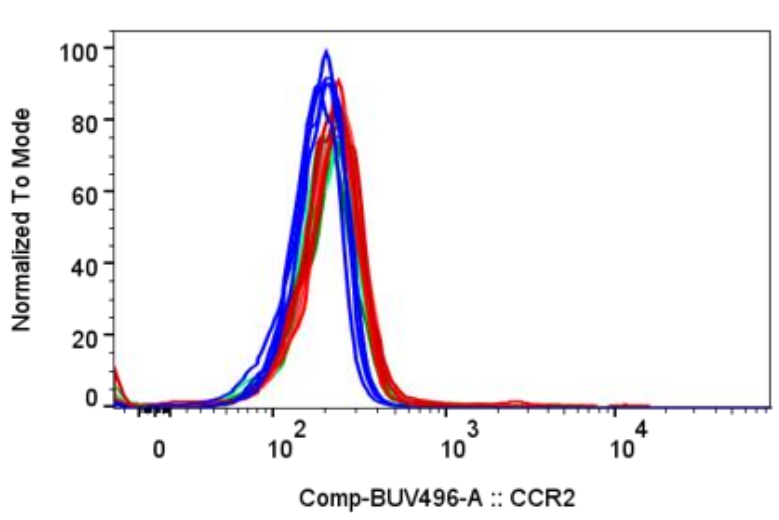

Sample Name	Subset Name	Geometric Mean : Comp-BUV496-A
BAL_3M 1.fcs	Q2: CD11b+ , CD11c+	177
BAL_3M 2.fcs	Q2: CD11b+ , CD11c+	192
BAL_3M 3.fcs	Q2: CD11b+ , CD11c+	188
BAL_3M 4.fcs	Q6: CD11b+ , CD11c+	173
BAL_17M 1.fcs	Q2: CD11b+ , CD11c+	215
BAL_17M 2.fcs	Q2: CD11b+ , CD11c+	221
BAL_17M 3.fcs	Q2: CD11b+ , CD11c+	227
BAL_17M 4.fcs	Q2: CD11b+ , CD11c+	215
BAL_17M 5.fcs	Q2: CD11b+ , CD11c+	235
BAL_17M 6.fcs	Q2: CD11b+ , CD11c+	226
BAL_9M 1.fcs	Q2: CD11b+ , CD11c+	216
BAL_9M 2.fcs	Q2: CD11b+ , CD11c+	218
BAL_9M 3.fcs	Q2: CD11b+ , CD11c+	219
BAL_9M 4.fcs	Q2: CD11b+ , CD11c+	205
BAL_9M 5.fcs	Q2: CD11b+ , CD11c+	209
BAL_9M 6.fcs	Q2: CD11b+ , CD11c+	204

Figure S21. CCR2 expression on mAMs in young and old mice. Part of Figure 5a. mAM was isolated from mice aged 3 months (3M), 9 months (9M), and 17 months (17M). Flow cytometry analysis of CCR2 expression in 3M- and 17M-old mice was conducted after gating on $CD45^{+}SiglecF^{+}CD11c^{+}CD11b^{+}$ cells. Histogram data represent individual MFI values from 3M-, 9M- and 17M-old mice. Individual mouse data are shown. N= 4 of 3M-old mice, 6 of 9M-old mice, and 17M-old mice. The mice cartoon schematic was created in BioRender. Pahari, S. (2025) https://BioRender.com/x07c111.

Figure S22

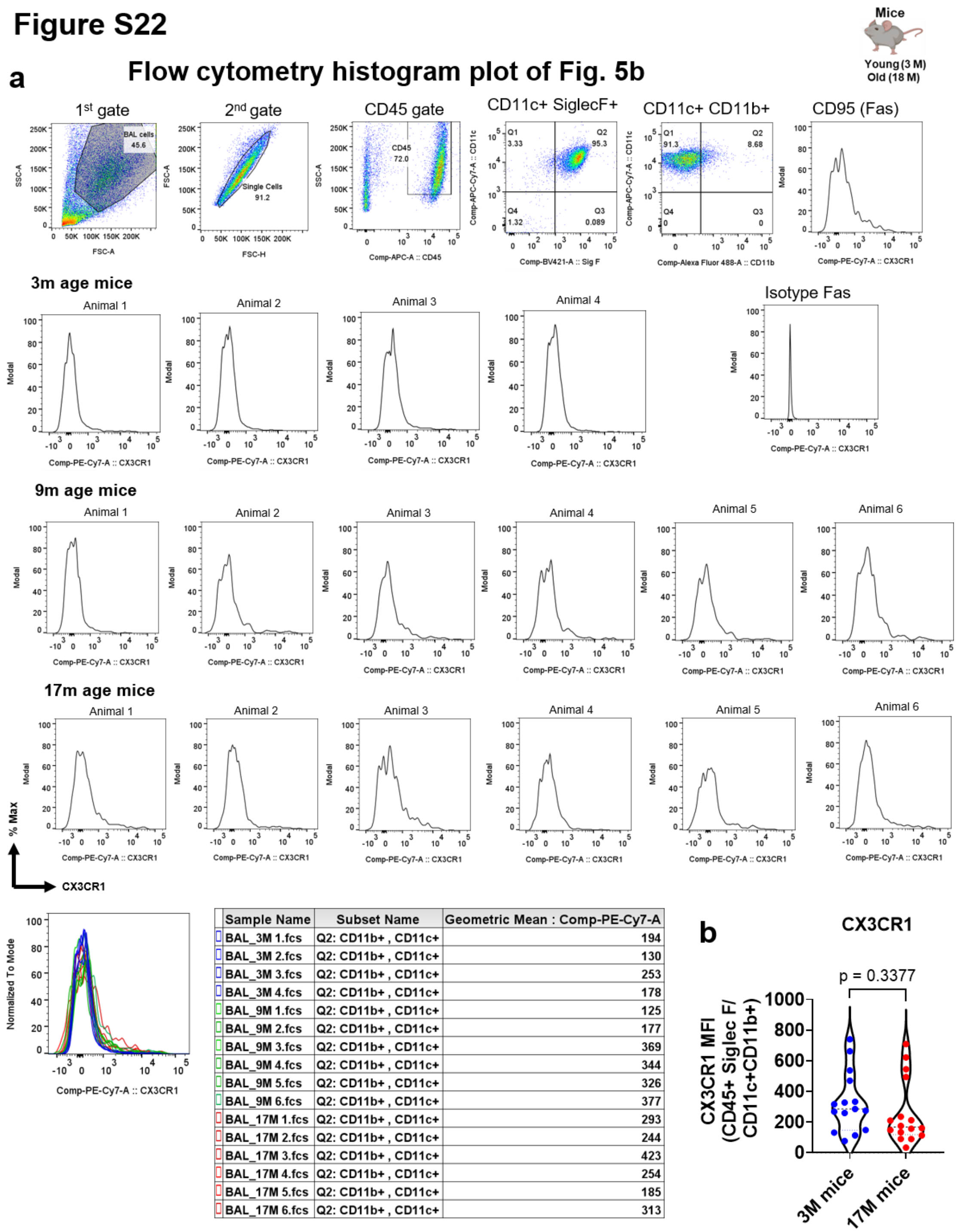

Sample Name	Subset Name	Geometric Mean : Comp-PE-Cy7-A
BAL_3M 1.fcs	Q2: CD11b+ , CD11c+	194
BAL_3M 2.fcs	Q2: CD11b+ , CD11c+	130
BAL_3M 3.fcs	Q2: CD11b+ , CD11c+	253
BAL_3M 4.fcs	Q2: CD11b+ , CD11c+	178
BAL_9M 1.fcs	Q2: CD11b+ , CD11c+	125
BAL_9M 2.fcs	Q2: CD11b+ , CD11c+	177
BAL_9M 3.fcs	Q2: CD11b+ , CD11c+	369
BAL_9M 4.fcs	Q2: CD11b+ , CD11c+	344
BAL_9M 5.fcs	Q2: CD11b+ , CD11c+	326
BAL_9M 6.fcs	Q2: CD11b+ , CD11c+	377
BAL_17M 1.fcs	Q2: CD11b+ , CD11c+	293
BAL_17M 2.fcs	Q2: CD11b+ , CD11c+	244
BAL_17M 3.fcs	Q2: CD11b+ , CD11c+	423
BAL_17M 4.fcs	Q2: CD11b+ , CD11c+	254
BAL_17M 5.fcs	Q2: CD11b+ , CD11c+	185
BAL_17M 6.fcs	Q2: CD11b+ , CD11c+	313

Figure S22. CX3CR1 expression on mAMs in young and old mice. Part of Figure 5b. mAM was isolated from mice aged 3 months (3M), 9 months (9M), and 17 months (17M). Flow cytometry analysis of CX3CR1 expression in 3M-, 9M- and 17M-old mice was conducted after gating on $CD45^+SiglecF^+CD11c^+CD11b^+$ cells. (a) Individual mouse histogram data are shown. N= 4 of 3M-old mice, 6 of 9M-old mice, and 17M-old mice. (b) Data pooled from 3 independent experiments with 15 mice/group. The mice cartoon schematic was created in BioRender. Pahari, S. (2025) https://BioRender.com/x07c111.

Figure S23

Flow cytometry pseudocolor dot plot of Fig. 6c

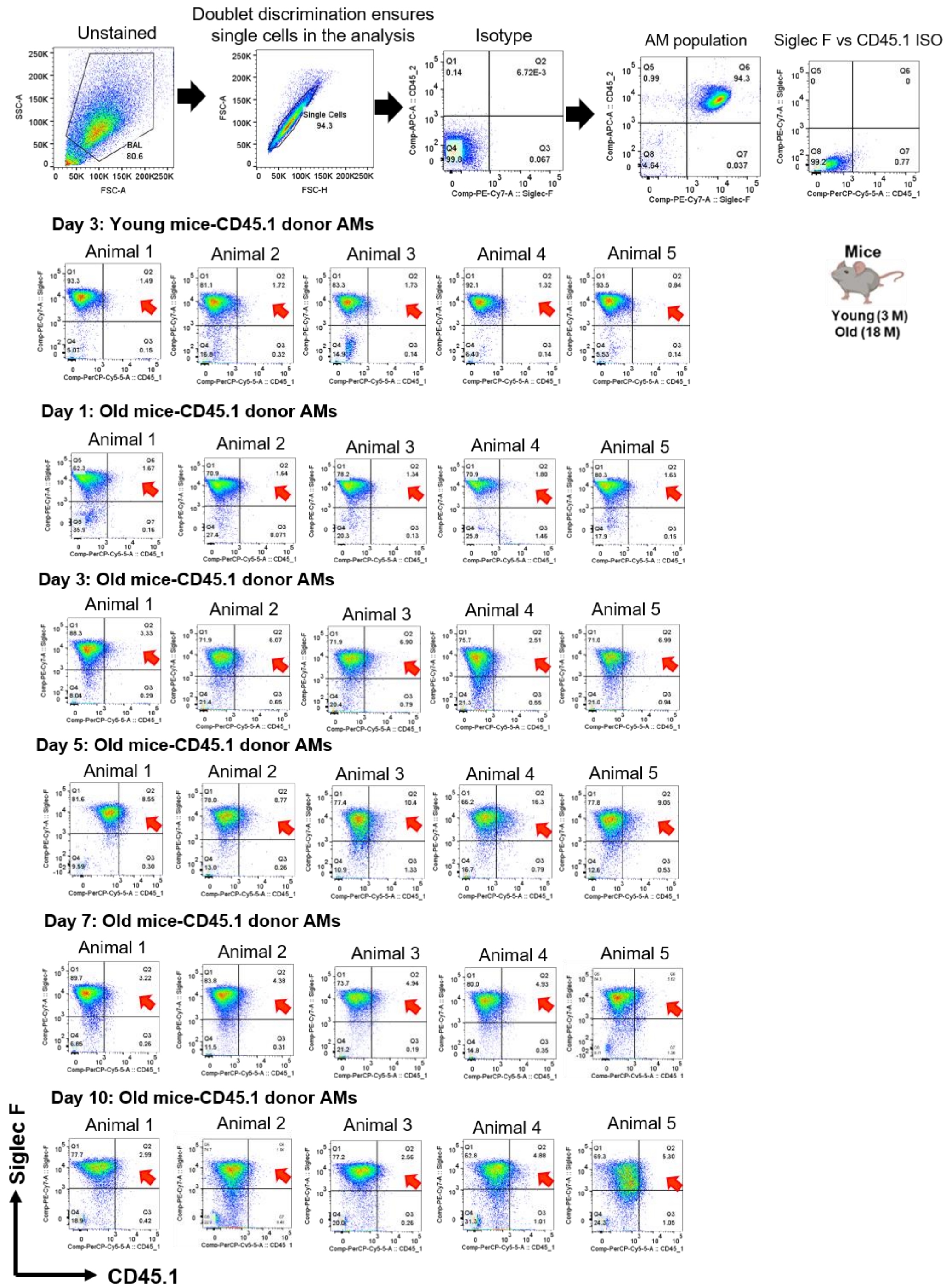

Figure S23. Flow cytometry pseudocolor dot plots corresponding to Figure 6c. Dot plots showing the gating strategy and representative individual mouse data for SiglecF^{+} CD45.1^{+} cells (quadrant Q2). The kinetics study includes days 1, 3, 5, 7, and 10. N=5 mice/group. Red arrow indicates the population of interest. The mice cartoon schematic was created in BioRender. Pahari, S. (2025) https://BioRender.com/x07c111.

Figure S24

Flow cytometry pseudocolor dot plot of Fig. 6e

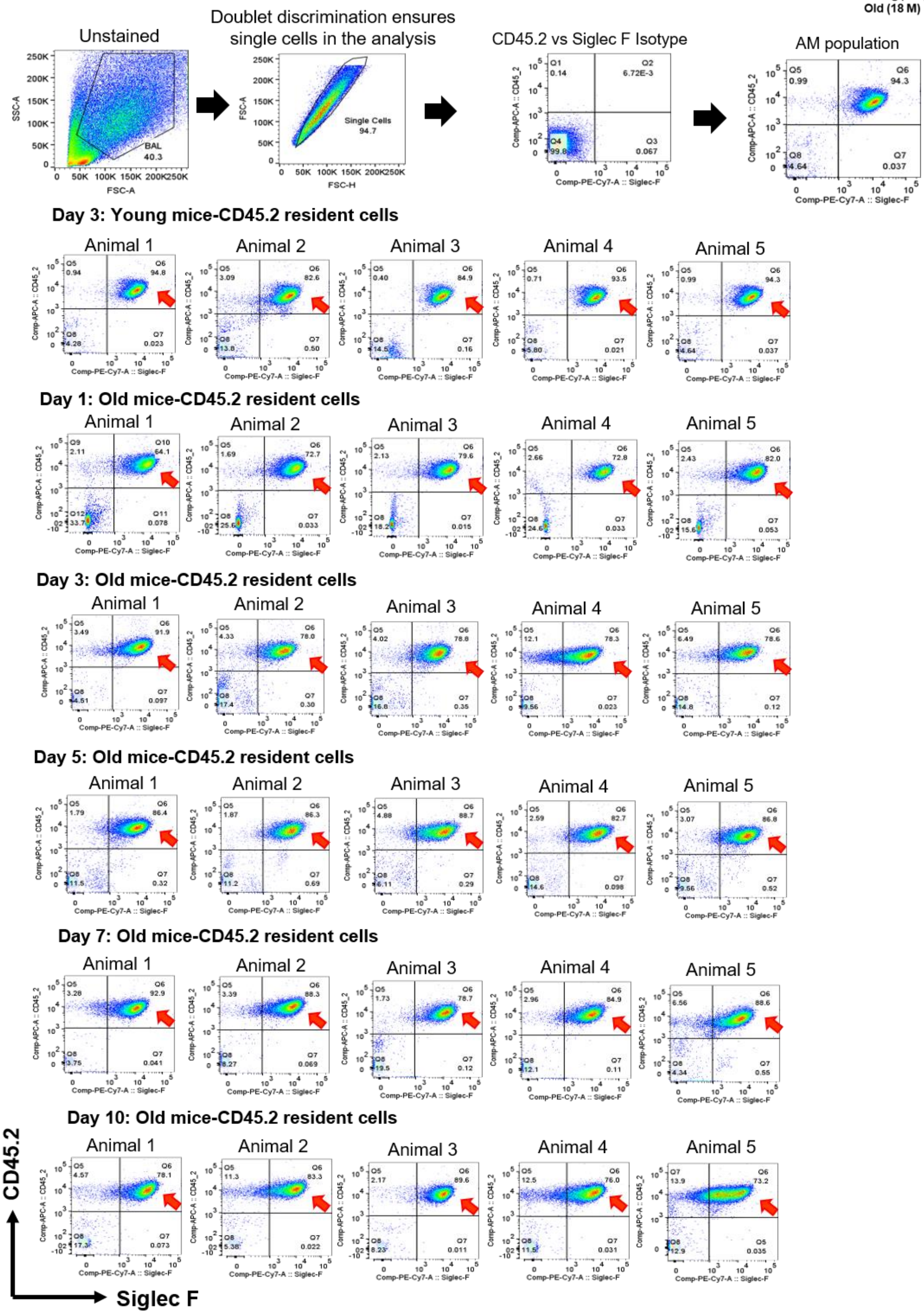

Figure S24. Flow cytometry pseudocolor dot plots corresponding to Figure 6e. Dot plots showing the gating strategy and representative individual mouse data for CD45.2$^+$ SiglecF$^+$ cells (quadrant Q6). The kinetics study includes days 1, 3, 5, 7, and 10. N=5 mice/group. Red arrow indicates the population of interest. The mice cartoon schematic was created in BioRender. Pahari, S. (2025) https://BioRender.com/x07c111.

Figure S25

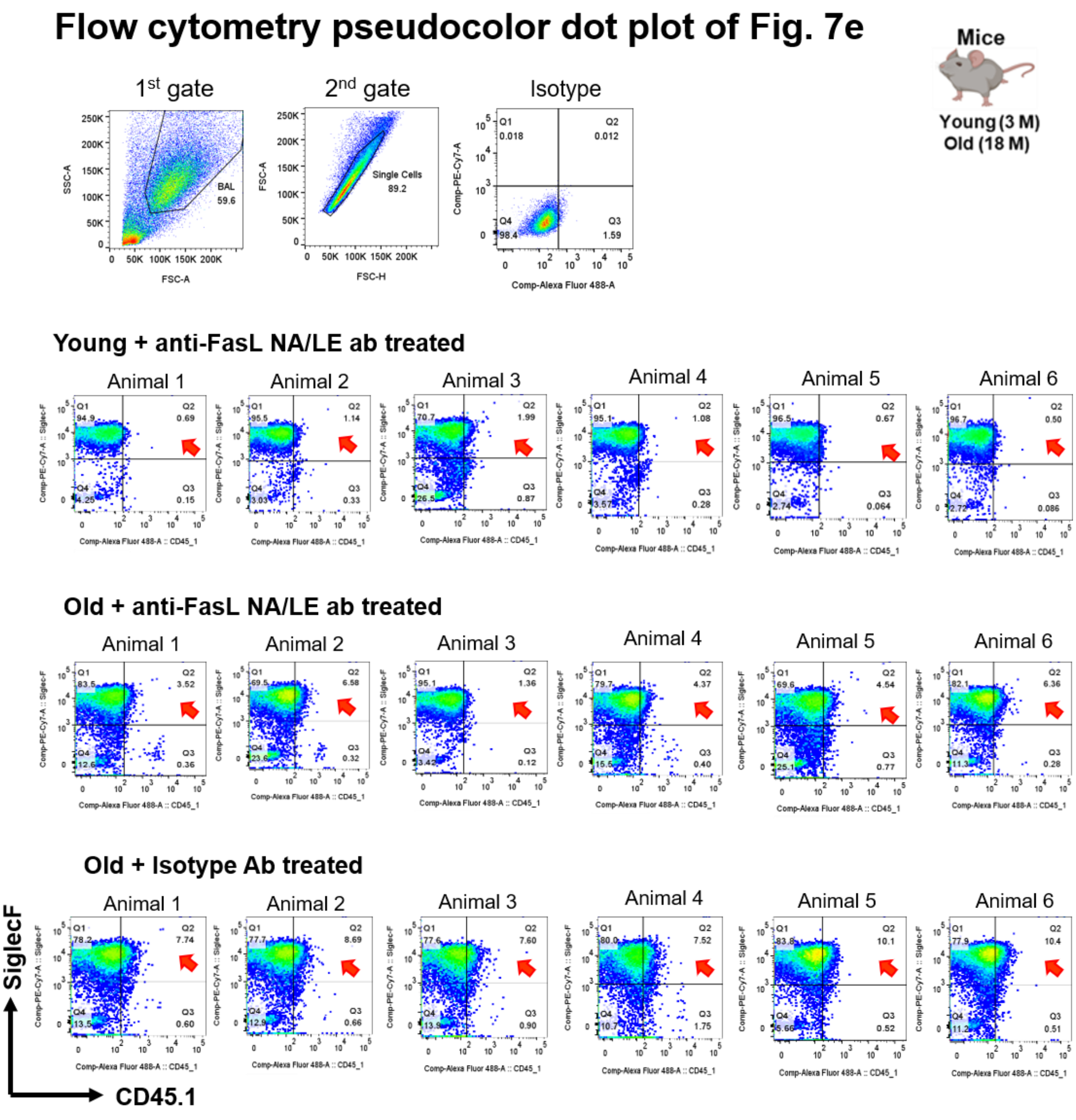

Figure S25. Flow cytometry pseudocolor dot plot of Figure 7e. Pseudocolor dot plots corresponding to **Figure 7e** showing the gating strategy and representative individual mouse data for SiglecF$^+$ CD45.1$^+$ cells (Percent, quadrant Q2). N=6 mice/group. Red arrow indicates the population of interest. The mice cartoon schematic was created in BioRender. Pahari, S. (2025) https://BioRender.com/x07c111.

Figure S26

Flow cytometry zebra plot of Fig. 7h

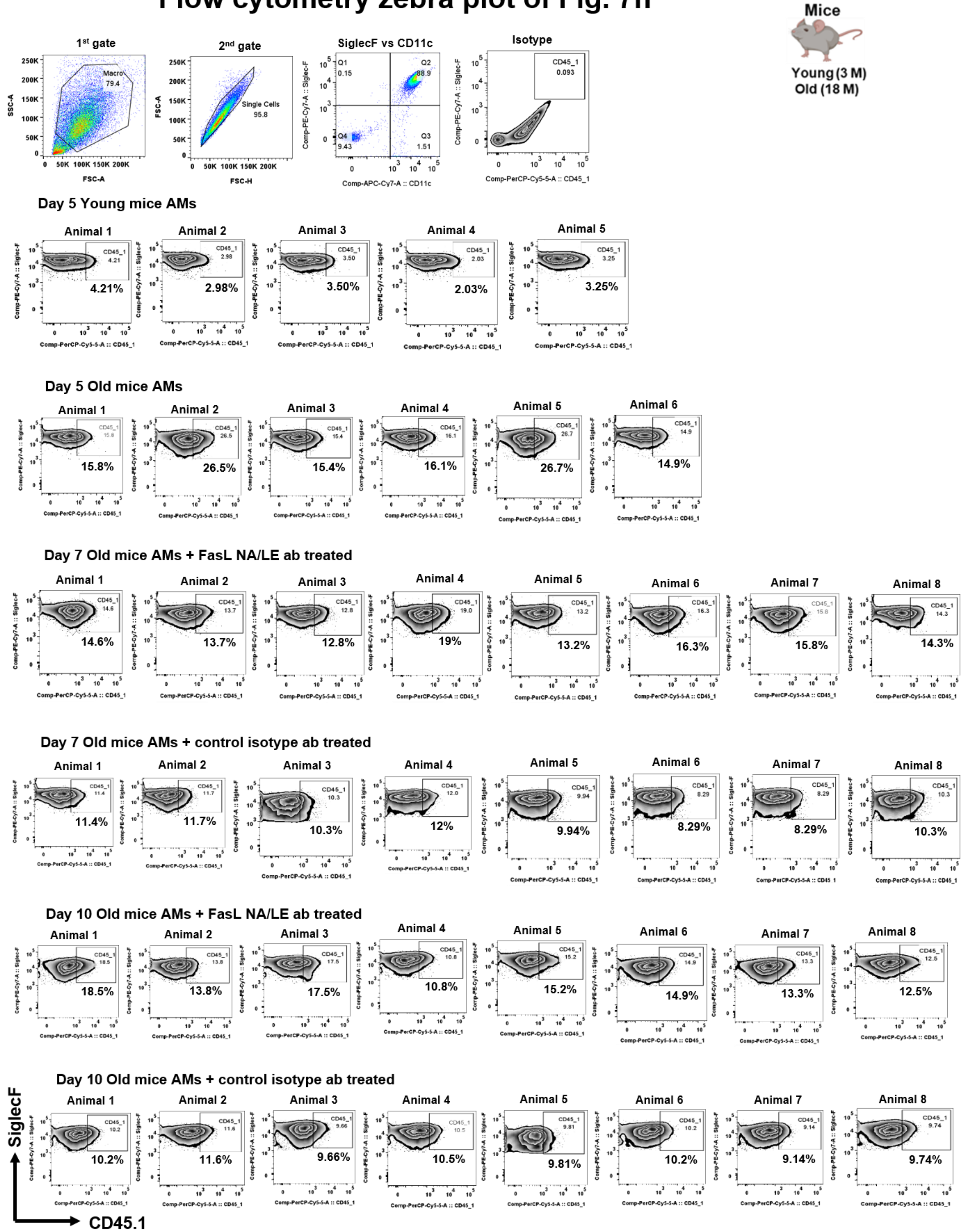

Figure S26. Flow cytometry zebra plot of Figure 7h. Zebra plots corresponding to **Figure 7h** showing the gating strategy and representative individual mouse data for SiglecF^{+} CD45.1^{+} cells (Percent). N=5 young control mice, 6 old control mice, and 8 experimental mice/group. The mice cartoon schematic was created in BioRender. Pahari, S. (2025) https://BioRender.com/x07c111.

Figure S27

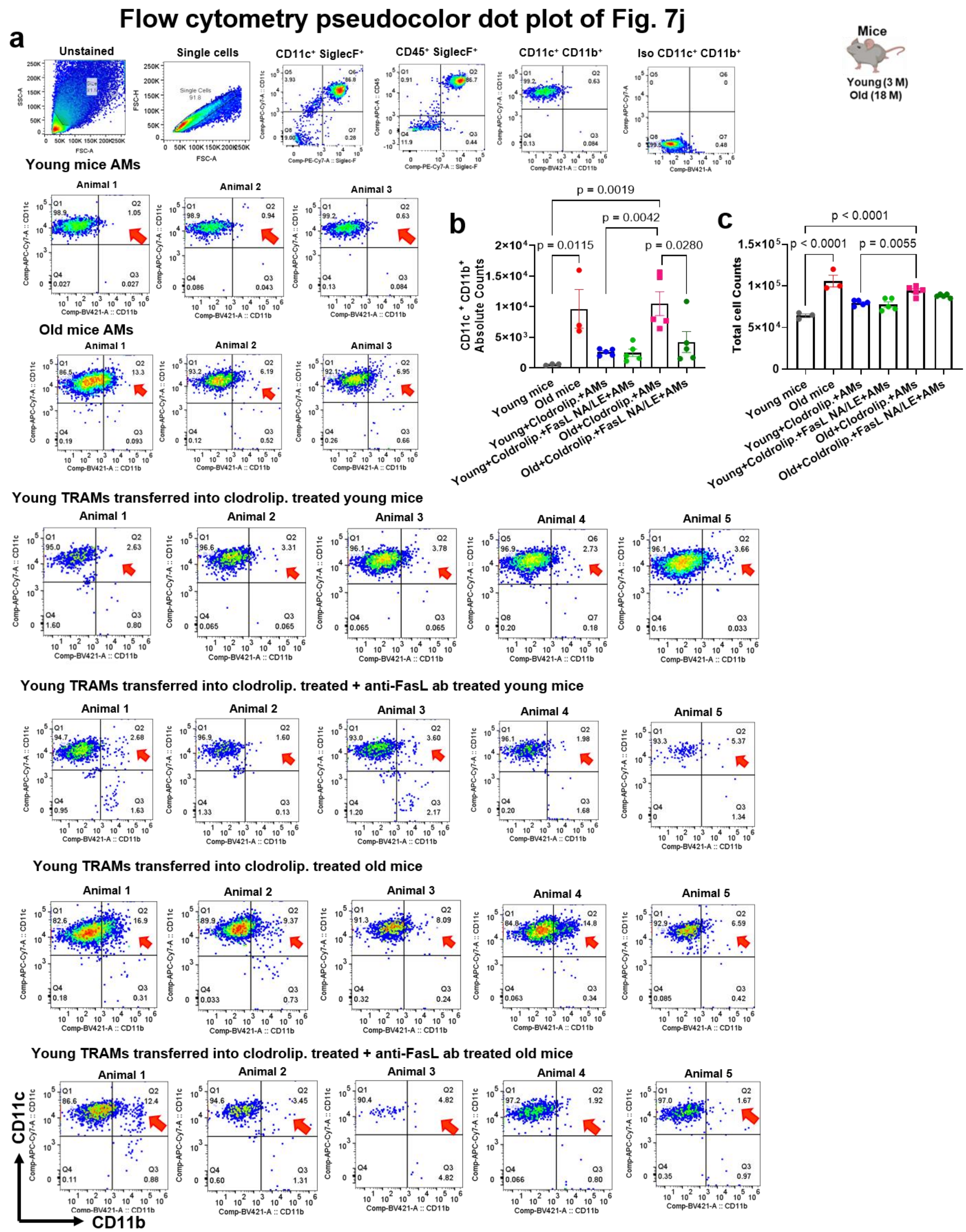

Figure S27. Flow cytometry pseudocolor dot plot of Figure 7j. (a) Flow cytometry pseudocolor dot plots corresponding to **Figure 7j** showing the gating strategy and representative individual mouse data for CD45^{+}SiglecF^{+} pre-gated, percent CD11c^{+}CD11b^{+} cells (quadrant Q2). N=3 young control mice, 3 old control mice, and 5 experimental mice/group. (b-c) Bar graphs show the absolute number of CD11c^{+}CD11b^{+} AMs recovered from BAL. Total AM counts for each experimental group are also shown. Red arrow indicates the population of interest. Data are expressed as mean ± SEM, analyzed by ordinary one-way ANOVA (two-sided) with Sidak's multiple comparisons test. The mice cartoon schematic was created in BioRender. Pahari, S. (2025) https://BioRender.com/x07c111.

Figure S28

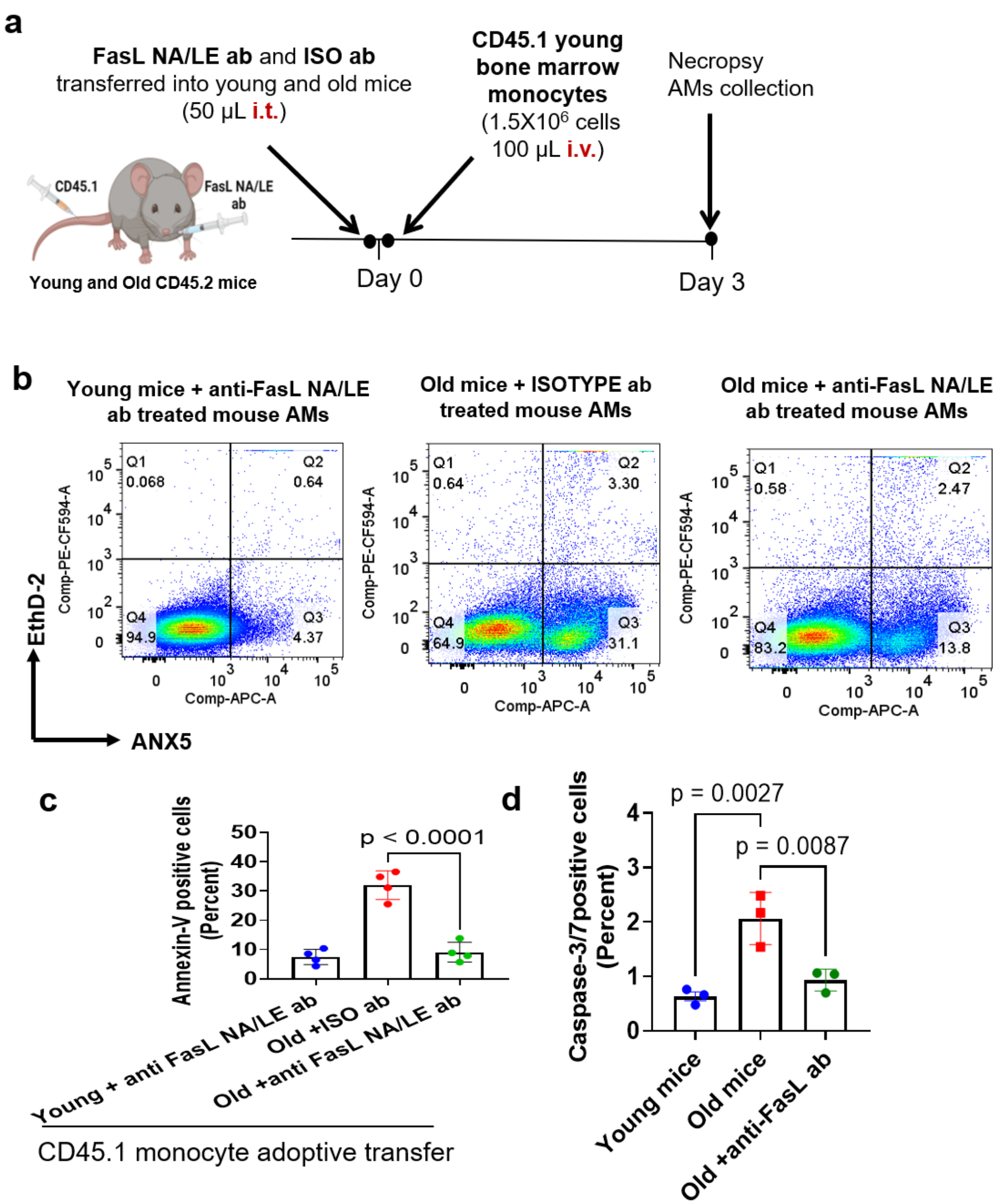

Figure S28. Adoptive transfer of CD45.1 monocytes and treatment with FasL NA/LE antibody extends the lifespan of AMs. Data are part of Figure 7. (a) Schematic representation of the experimental approach is shown. Young and old mice were treated by i.t. installation of FasL NA/LE Ab (50 µL, 0.5 mg/mL), or an isotype control Ab. Concurrently, CD45.1 bone marrow monocytes (isolated by magnetic sorting for Ly6c+, negative selection kit) were transferred i.v. On day 3 mAMs were isolated from each group by BAL to analyze for apoptosis by flow cytometry. Created in BioRender. Pahari, S. (2026) https://BioRender.com/q98c015. (b) Flow cytometry dot plot analysis shows Annexin V (ANX5) and Ethidium Homodimer-2 (EthD-2) viability assay on day 3. FasL NA/LE Ab treatment reduces Fas-mediated apoptosis in old mAMs. (c) Bar graph shows the percentage of cumulative annexin-positive cells from 4 mice/group (N=4). Data are expressed as mean ± SEM, analyzed by ordinary one-way ANOVA (two-sided) with Sidak's multiple comparisons test. (d) Flow cytometry-generated bar graphs illustrate the induction of Caspase-3/7 mediated apoptosis in old mice total mAMs, which was decreased following treatment with FasL NA/LE Ab. N=3 mice/group. Data are expressed as mean ± SEM, analyzed by ordinary one-way ANOVA (two-sided) with Sidak's multiple comparisons test.

Figure S29

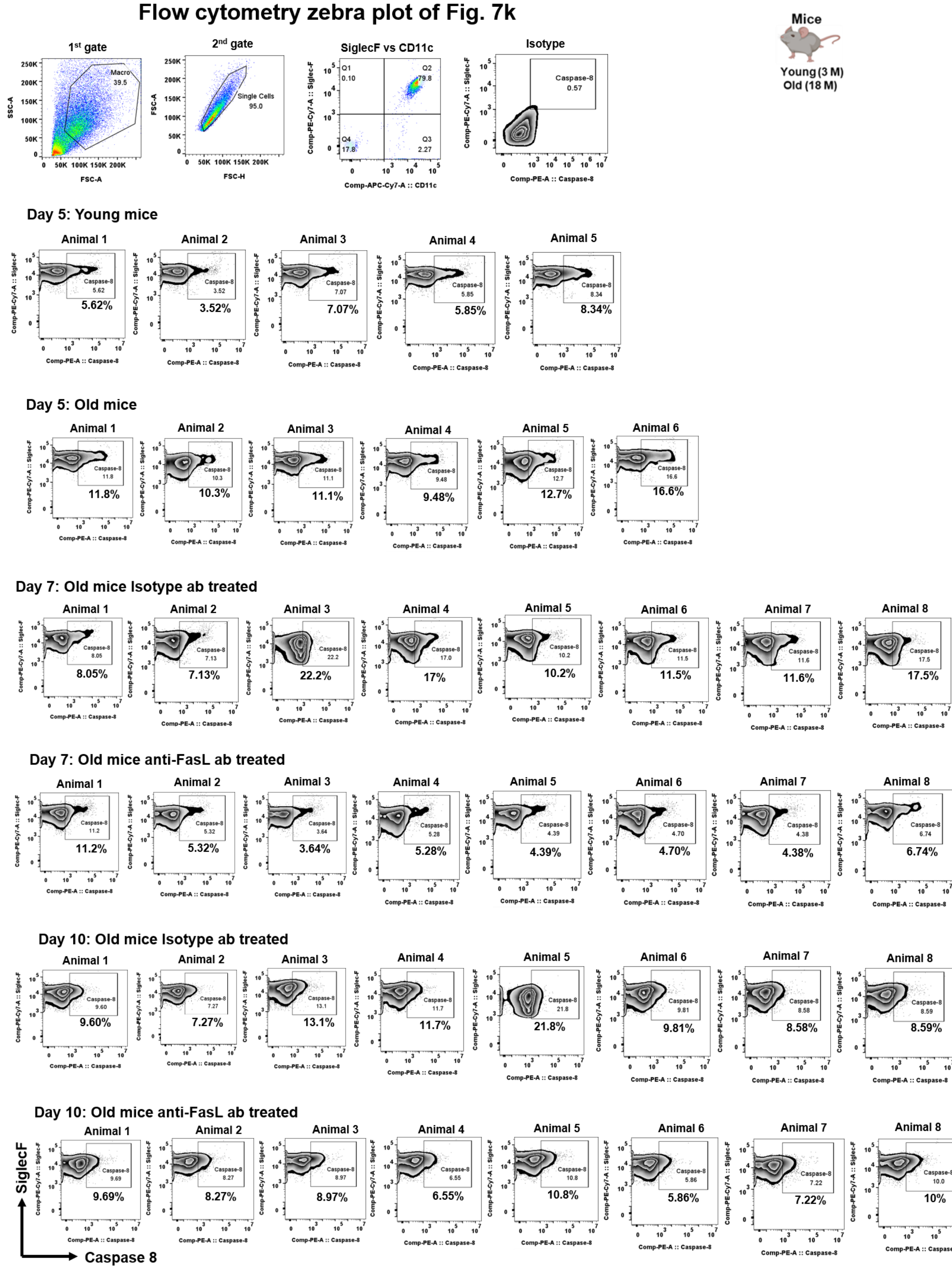

Figure S29. Flow cytometry zebra plots correspond to Figure 7k. Zebra plots showing the gating strategy and representative individual mouse data for Caspase-8$^+$ cells (percentage) within SiglecF$^+$CD11c$^+$ gated populations. N=5 young control mice, 6 old control mice, and 8 experimental mice/group. The mice cartoon schematic was created in BioRender. Pahari, S. (2025) https://BioRender.com/x07c111.

Figure S30

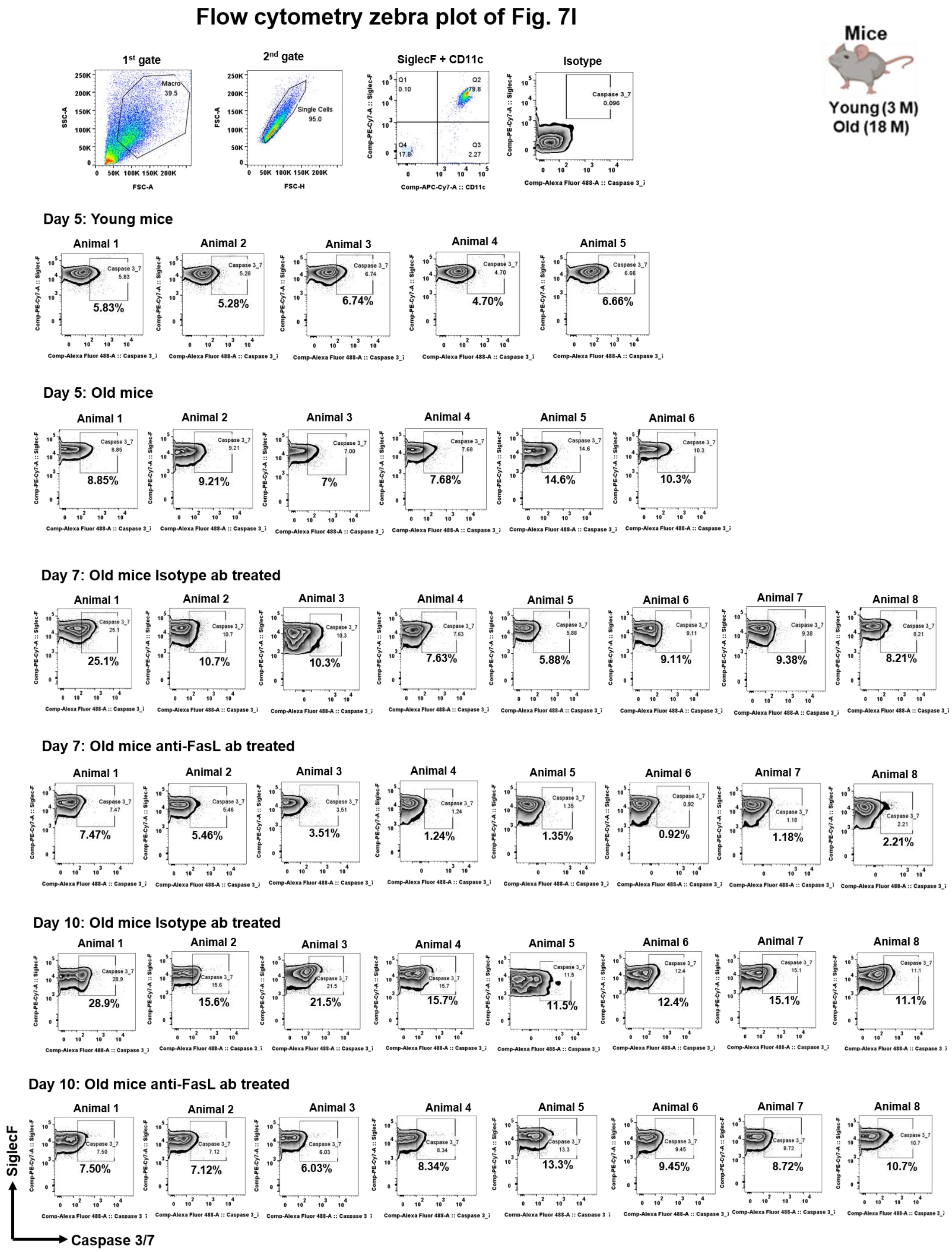

Figure S30. Flow cytometry zebra plots corresponding to Figure 7I. Zebra plots showing the gating strategy and representative individual mouse data for Caspase-3/7^{+} cells (percentage) within SiglecF^{+}CD11c^{+} gated populations. N=5 young control mice, 6 old control mice, and 8 experimental mice/group. The mice cartoon schematic was created in BioRender. Pahari, S. (2025) https://BioRender.com/x07c111.

Table S1: Demographic & Clinical Characteristics of the Participants in this study

OSU IRB 2012H0135 and TxBiomed IRB HSC20170673H

BAL Donar ID	Age (years)	Gender	Ethnicity	BCG Vaccine	TST/IGRA
	**Adult (20-45 years)**				
BAL-A1	39	M	W	N	N
BAL-A2	40	M	W	N	N
BAL-A4	44	M	B	N	N
BAL-A5	26	M	W	N	N
BAL-A17	25	F	W	N	N
BAL-A20	36	M	W	Y	N
BAL-A22	35	F	U	U	U
BAL-A23	23	M	H	U	N
	**Elderly (60-74 years)**				
BAL-E3	60	F	W	N	N
BAL-E6	65	M	W	N	N
BAL-E8	70	F	W	N	N
BAL-E9	73	F	W	N	N
BAL-E10	74	F	W	N	N
BAL-E12	65	F	W	N	N
BAL-E13	69	F	W	N	N
BAL-E14	70	F	W	N	N
BAL-E16	71	M	W	N	N
BAL-E18	73	M	W	N	N
BAL-E19	74	F	W	U	N
BAL-E21	70	M	H	U	N
BAL-E25	61	F	W	U	N

M: Male; F: Female; W: White; B: Black; H: Hispanic; N: No; Y: Yes; U: Unknown

Table S1: Demographic & Clinical Characteristics of the Participants in this Study. The table provides descriptive information on the human participants in this study, including age distribution and other relevant details.

Table S2: Key resources Table

REAGENT or RESOURCE	SOURCE	IDENTIFIER	Clone name	Abs
**Human Antibodies**				
BD Horizon Brilliant Stain Buffer	BD Bioscience	Cat# 563794 RRID: AB_2869750		1:10
Cell Staining Buffer	BioLegend	Cat# 420201		
Human TruStain FcX (Fc Receptor Blocking Solution)	BioLegend	Cat# 422302 RRID: AB_2818986		1:50
TruStain FcX PLUS (anti-mouse CD16/32) Antibody	BioLegend	Cat# 156604 RRID: AB_2783137	S17011E	1:50
Brilliant Violet 421 anti-mouse/human CD11b Antibody	BioLegend	Cat# 101236 RRID: AB_10897942	M1/70	1:70
Brilliant Violet 421 Rat IgG2b, κ Isotype Ctrl	BioLegend	Cat# 400640 RRID: AB_10895758	RTK4530	1:70
PE anti-human CD11c Antibody	BioLegend	Cat#301606 RRID: AB_314176	3.9	1:20
PE Mouse IgG1, κ Isotype Ctrl Antibody	BioLegend	Cat#400112 RRID: AB_2847829	MOPC-21	1:20
Alexa Fluor 700 anti-human CD11c Antibody	BioLegend	Cat#301648 RRID: AB_314176	3.9	1:20
Alexa Fluor 700 Mouse IgG1, κ Isotype Ctrl Antibody	BioLegend	Cat#400144 RRID: AB_2819923	MOPC-21	1:20
APC/Cyanine7 anti-human CD11b Antibody	BioLegend	Cat#301342 RRID: AB_2563395	ICRF44	1:20
APC/Cyanine7 Mouse IgG1, κ Isotype Ctrl Antibody	BioLegend	Cat#400128 RRID: AB_2892538	MOPC-21	1:20
Brilliant Violet 785 anti-human CD11c Antibody	BioLegend	Cat# 337252 RRID: AB_3097503	Bu15	1:20
Brilliant Violet 785 Mouse IgG1, κ Isotype Ctrl Antibody	BioLegend	Cat# 400170 RRID: AB_11219601	MOPC-21	1:20
Alexa Fluor 488 anti-human CD206 (MMR) Antibody	BioLegend	Cat# 321114 RRID: AB_571874	15-2	1:20
Alexa Fluor 488 Mouse IgG1, κ Isotype Ctrl (FC) Antibody	BioLegend	Cat# 400129 RRID: AB_2890263	MOPC-21	1:20
Brilliant Violet 605 anti-human HLA-DR Antibody	BioLegend	Cat# 307640 RRID: AB_2561913	L243	1:20
Brilliant Violet 605 Mouse IgG2a, κ Isotype Ctrl Antibody	BioLegend	Cat# 400270 RRID: AB_3097669	MOPC-173	1:20
PerCP/Cyanine5.5 anti-human CD16 Antibody	BioLegend	Cat# 302028 RRID: AB_893262	3G8	1:20
PerCP/Cyanine5.5 Mouse IgG1, κ Isotype Ctrl Antibody	BioLegend	Cat# 400150 RRID: AB_893680	MOPC-21	1:20
APC anti-human CD36 Antibody	BioLegend	Cat# 336208 RRID: AB_1279224	5-271	1:20
APC Mouse IgG2a, κ Isotype Ctrl (FC) Antibody	BioLegend	Cat# 400222 RRID: AB_2891178	MOPC-173	1:20
PerCP/Cyanine5.5 anti-human CD64 Antibody	BioLegend	Cat# 305024 RRID: AB_2561585	10.1	1:20

PerCP/Cyanine5.5 Mouse IgG1, κ Isotype Ctrl Antibody	BioLegend	Cat# 400150 RRID: AB_893680	MOPC-21	1:20
Brilliant Violet 605 anti-human CD86 Antibody	BioLegend	Cat# 305430 RRID:AB_2563824	IT2.2	1:20
Brilliant Violet 605 Mouse IgG2b, κ Isotype Ctrl Antibody	BioLegend	Cat# 400350 RRID: AB_3097666	MPC-11	1:20
Brilliant Violet 605 anti-human CD192 (CCR2) Antibody	BioLegend	Cat# 357214 RRID: AB_2562702	K036C2	1:20
Brilliant Violet 605 Mouse IgG2a, κ Isotype Ctrl Antibody	BioLegend	Cat# 400270 RRID: AB_3097669	MOPC-173	1:20
PerCP/Cyanine5.5 anti-human CX3CR1 Antibody	BioLegend	Cat# 341614 RRID: AB_11219203	2A9-1	1:20
PerCP/Cyanine5.5 Rat IgG2b, κ Isotype Ctrl Antibody	BioLegend	Cat# 400632 RRID: AB_893693	RTK4530	1:20
PE/Dazzle 594 anti-human CD14 Antibody	BioLegend	Cat# 301852 RRID: AB_2629575	M5E2	1:20
PE/Dazzle 594 Mouse IgG2a, κ Isotype Ctrl Antibody	BioLegend	Cat# 400276 RRID: AB_3097694	MOPC-173	1:20
Alexa Fluor 488 anti-human CD115 (CSF-1R) Antibody	BioLegend	Cat# 347311 RRID: AB_2566757	9-4D2-1E4	1:20
Alexa Fluor 488 Rat IgG1, κ Isotype Ctrl Antibody	BioLegend	Cat# 400417 RRID: AB_389319	RTK2071	1:20
PE/Dazzle 594 anti-human CD95 (Fas) Antibody	BioLegend	Cat# 305634 RRID: AB_2564221	DX2	1:20
PE/Dazzle 594 Mouse IgG1, κ Isotype Ctrl Antibody	BioLegend	Cat# 400176 RRID: AB_2923261	MOPC-21	1:20
PerCP-Cy5.5 anti-human CD14	Calibre Scientific	Cat# CAPR-103465		1:20
**Mouse Antibodies**				
BD OptiBuild BUV395 Hamster Anti-Mouse CD95	BD Bioscience	Cat# 740254 RRID: AB_2739999	Jo2	1:50
BD Horizon BUV395 Hamster IgG2, κ Isotype Control	BD Bioscience	Cat# 565820 RRID: AB_2869719	B81-3	1:50
BD OptiBuild BUV496 Rat Anti-Mouse CD192 (CCR2)	BD Bioscience	Cat# 750043 RRID: AB_2874260	475301	1:50
BUV496 Rat IgG2b, κ Isotype Control	BD Bioscience	Cat# 612954 RRID: AB_2869598	R35-38	1:50
Brilliant Violet 421 anti-mouse CD170 (SiglecF) Antibody	BioLegend	Cat# 155509 RRID: AB_2810421	S17007L	1:167
Brilliant Violet 421 Rat IgG2a, κ Isotype Ctrl Antibody	BioLegend	Cat# 400536 RRID: AB_10933427	RTK2758	1:167
Brilliant Violet 650 anti-mouse CD3 Antibody	BioLegend	Cat# 100229 RRID: AB_11204249	17A2	1:50
Brilliant Violet 650 Rat IgG2b, κ Isotype Ctrl Antibody	BioLegend	Cat# 400651 RRID: AB_3097674	RTK4530	1:50
BD OptiBuild BV786 Mouse Anti-Mouse CD64 a and b Alloantigens	BD Bioscience	Cat# 741024 RRID: AB_2740644	X54-5/7.1	1:50

BV786 Mouse IgG1, k Isotype Control	BD Bioscience	Cat# 563330 RRID: AB_2869484	X40	1:50
Alexa Fluor 488 anti-mouse/human CD11b Antibody	BioLegend	Cat# 101217 RRID: AB_389305	M1/70	1:70
Alexa Fluor 488 Rat IgG2b, κ Isotype Ctrl Antibody	BioLegend	Cat# 400625 RRID: AB_389321	RTK4530	1:70
PerCP/Cyanine5.5 anti-mouse CD115 (CSF-1R) Antibody	BioLegend	Cat# 135526 RRID: AB_2566461	AFS98	1:60
PerCP/Cyanine5.5 Rat IgG2a, κ Isotype Ctrl Antibody	BioLegend	Cat# 400532 RRID: AB_2864286	RTK2758	1:60
PE anti-mouse Ly-6C Antibody	BioLegend	Cat# 128008 RRID: AB_1186132	HK1.4	1:100
PE Rat IgG2c, κ Isotype Ctrl Antibody	BioLegend	Cat# 400707 RRID: AB_326573	RTK4174	1:100
PE/Cyanine7 anti-mouse CX3CR1 Antibody	BioLegend	Cat# 149016 RRID: AB_2565699	SA011F11	1:167
PE/Cyanine7 Mouse IgG2a, κ Isotype Ctrl Antibody	BioLegend	Cat# 400254 RRID: AB_10697032	MOPC-173	1:167
APC anti-mouse CD45.2 Antibody	BioLegend	Cat# 109814 RRID: AB_389210	104	1:60
APC Mouse IgG2a, κ Isotype Ctrl (FC) Antibody	BioLegend	Cat# 400222 RRID: AB_2891178	MOPC-173	1:60
Alexa Fluor 700 anti-mouse Ly-6G Antibody	BioLegend	Cat# 127622 RRID: AB_10643269	1A8	1:200
Alexa Fluor 700 Rat IgG2a, κ Isotype Ctrl Antibody	BioLegend	Cat# 400528 RRID: AB_2923249	RTK2758	1:200
APC/Cyanine7 anti-mouse CD11c Antibody	BioLegend	Cat# 117324 RRID: AB_830646	N418	1:100
APC/Cyanine7 Armenian Hamster IgG Isotype Ctrl Antibody	BioLegend	Cat# 400927 RRID: AB_2923251	HTK888	1:100
Brilliant Violet 421 anti-mouse/human CD11b Antibody	BioLegend	Cat# 101236 RRID: AB_10897942	M1/70	1:70
Brilliant Violet 421 Rat IgG2b, κ Isotype Ctrl Antibody	BioLegend	Cat# 400640 RRID: AB_11204247	RTK4530	1:70
BD OptiBuild BV786 Rat Anti-Mouse CD195	BD Bioscience	Cat# 743700 RRID: AB_2741682	C34-3448	1:50
Rat IgG2c, κ	BD Bioscience	Cat# RRID		1:50
PE anti-mouse CD206 (MMR) Antibody	BioLegend	Cat# 141706 RRID: AB_1089575	C068C2	1:50
PE Rat IgG2a, κ Isotype Ctrl Antibody	BioLegend	Cat# 400508 RRID: AB_326530	RTK2758	1:50
PE/Cyanine7 anti-mouse CD170 (SiglecF) Antibody	BioLegend	Cat# 155528 RRID: AB_2890715	S17007L	1:167
PE/Cyanine7 Rat IgG2a, κ Isotype Ctrl	BioLegend	Cat# 400522 RRID: AB_326542	RTK2758	1:167
APC anti-mouse CD45.2 Antibody	BioLegend	Cat# 109814 RRID: AB_389210	104	1:60

APC Mouse IgG2a, κ Isotype Ctrl (FC) Antibody	BioLegend	Cat# AB_2891178 RRID: 400222		1:60
APC Rat Anti-Mouse CD45	BD Bioscience	Cat# 559864 RRID: AB_398672	30-F11	1:60
APC Rat IgG2b, κ Isotype Control	BD Bioscience	Cat# 553991 RRID: AB_10050405	A95-1	1:60
Brilliant Violet 650 anti-mouse F4/80 Antibody	BioLegend	Cat# 123149 RRID: AB_2564589	BM8	1:50
Brilliant Violet 650 Rat IgG2a, κ Isotype Ctrl Antibody	BioLegend	Cat# 400542 RRID: AB_11123911	RTK2758	1:50
PerCP/Cyanine5.5 anti-mouse CD45.1 Antibody	BioLegend	Cat# 110728 RRID: AB_893346	A20	1:80
PerCP/Cyanine5.5 Mouse IgG2a, κ Isotype Ctrl Antibody	BioLegend	Cat# 400258 RRID: AB_10695169	MOPC-173	1:80
Alexa Fluor 488 anti-mouse CD45.1 Antibody	BioLegend	Cat# 110718 RRID: AB_492862	A20	1:100
Alexa Fluor 488 Mouse IgG2a, κ Isotype Ctrl Antibody	BioLegend	Cat# 400233 RRID: AB_3076443	MOPC-173	1:100
CellEvent Caspase-3/7 Green Flow Cytometry Assay Kit	Thermo Fisher Scientific/Invitrogen	Cat# C10427 RRID		1:80
Caspase-8 p18 Antibody (E-8): sc-166320	Santa Cruz Biotechnology	Cat# sc-166320 RRID	E-8	1:100
Purified NA/LE Hamster Anti-Mouse CD178	BD Bioscience	Cat# 555290 RRID: AB_395708	MFL3	50 µg
Purified Hamster IgG1, κ Isotype Control	BD Bioscience	Cat# 553969 RRID	A19-3	50 µg
Zombie Green Fixable Viability Kit	BioLegend	Cat# 423112		1:200
APC Annexin V	BioLegend	Cat# 640941		1:30
Annexin V Binding Buffer	BioLegend	Cat# 422201		
PKH26 Red Fluorescent Cell Linker Kit for Phagocytic Cell Labeling	Sigma Aldrich	Cat# PKH26PCL		10µM (1:10)
**Bacterial and virus strains**				
**Biological samples**				
Mouse alveolar macrophages	This manuscript	N/A		
Human alveolar macrophages	This manuscript	N/A		
Baboon alveolar macrophages	This manuscript	N/A		
Heparinized whole blood from mice	This manuscript	N/A		
**Chemicals, peptides, and recombinant proteins**				
Clodronate Liposomes	Liposoma research	Cat# CP-005-005		250 µg/50 µL

Control Liposomes (PBS)	Liposoma research	Cat# CP-005-005		250 µg/50 µL
RPMI 1640 Medium, GlutaMAX Supplement	Gibco / Invitrogen, Thermo Fisher Scientific	Cat# 11875-093; Cat# 61870044		
Human AB serum	Sigma Aldrich	Cat# H4522		1:10
Lymphoprep	Stem Cell Technologies	Cat# 07851		
Ficoll-paque PLUS	GE healthcare/Sigma	Cat# 17-1440-03		
EDTA, 0.5M	Thermo Fisher Scientific	Cat# 15575020		100 µM
Heparin sodium, 1000 U/mL	Nationwide medical surgical/ Sagent Pharmaceuticals	Cat #25021040010		1000 U/mL
Bovine Serum Albumin (BSA)	Millipore Sigma			0.2% BSA
Gibco cell dissociation buffer	Thermo Fisher Scientific	Cat# 13-150-016		
0.9% sodium chloride, irrigation	Abbott Laboratories			
MitoSOX Mitochondrial superoxide indicators Red	Thermo Fisher Scientific	Cat# M36008		
TRIzol reagent	Invitrogen/ Thermo Fisher Scientific	Cat# 15596026		
Hema 3 staining	Fisher Scientific	Cat# 22122911		
Amicon Ultra Centrifugal Filter, 10 kDa MWCO	Millipore Sigma	Cat# UFC801024		
DPBS 1x, no calcium, no magnesium	Thermo Fisher Scientific	Cat# 14190169		
**Critical commercial assays**				
Classical monocyte isolation kit	Miltenyi	Cat# 130-117-337		
EasySep Mouse Monocyte Isolation Kit	Stem Cell technologies	Cat# 19861		
EasySep Buffer	Stem Cell technologies	Cat# 20144		

High-Capacity RNA-to-cDNA Kit	Applied Biosystems	Cat# 4387406		
TaqMan Fast Universal PCR Master Mix (2x), no AmpErase UNG	Thermo Fisher Scientific	Cat# 4352042		
Luminex mouse Discovery Assay (10-Plex)	R&D Systems, Inc.	Cat# LXSAMSM-10kit<br>Code: mxmbhKZn		
Oligomycin A	Millipore Sigma	Cat# 75351-5MG		
FCCP (Carbonyl cyanide 4-(trifluoromethoxy)phenylhydrazone)	Millipore Sigma	Cat# C2920-10MG		
Antimycin A from Streptomyces sp.	Millipore Sigma	Cat# A8674-50MG		
Rotenone	Millipore Sigma	Cat# 557368-1GM		
2-Deoxy-D-glucose	Millipore Sigma	Cat# D6134-5G		
Collagenase from Clostridium histolyticum	Millipore Sigma	Cat# C7657-500MG		
Deoxyribonuclease I from bovine pancreas	Millipore Sigma	Cat# D5025-150KU		
**qRT-PCR Primers**				
**Human Primers**				
CD11B	Thermo Fisher Scientific	Cat# Hs00167304_m1		
CD11C	Thermo Fisher Scientific	Cat# Hs00174217_m1		
MRC1 (CD206)	Thermo Fisher Scientific	Cat# Hs00267207_m1		
NOS2	Thermo Fisher Scientific	Cat# Hs01075529_m1		
HIF1A	Thermo Fisher Scientific	Cat# Hs00153153_m1		
FAS	Thermo Fisher Scientific	Cat# Hs00236330_m1		
TLR2	Thermo Fisher Scientific	Cat# Hs00152932_m1		

PIEZO1	Thermo Fisher Scientific	Cat# Hs00207230_m1		
IFNB	Thermo Fisher Scientific	Cat# Hs01077958_s1		
IL10	Thermo Fisher Scientific	Cat# Hs00961622_m1		
CCR2	Thermo Fisher Scientific	Cat# Hs00356601_m1		
CX3CR1	Thermo Fisher Scientific	Cat# Hs00365842_m1		
NDUFAF6	Thermo Fisher Scientific	Cat# Hs00901870_m1		
LDHA	Thermo Fisher Scientific	Cat# Hs01378790_g1		
ACTB	Thermo Fisher Scientific	Cat# Hs01060665_g1		
**Mouse primers**				
Hif1α		Forward Primer ACCTTCATCGGAAACTCCAAAG Reverse Primer CTGTTAGGCTGGGAAAAGTTAGG		
Cox2		Forward Primer CTGGCGCTCAGCCATACAG Reverse Primer CGCACTTATACTGGTCAAATCCC		
Cd74		Forward Primer AGTGCGACGAGAACGGTAAC Reverse Primer CGTTGGGGAACACACACCA		
Mif		Forward Primer GCCAGAGGGGTTTCTGTCG Reverse Primer GTTCGTGCCGCTAAAGTCA		

Glut1		Forward Primer CAGTTCGGCTATA ACACTGGTG Reverse Primer GCCCCGACAGAG AAGATG		
Hk2		Forward Primer TGATCGCCTGCTT ATTCACGG Reverse Primer AACCGCCTAGAA ATCTCCAGA		
Pfkb3		Forward Primer CCCAGAGCCGGG TACAGAA Reverse Primer GGGGAGTTGGTC AGCTTCG		
Gls1		Forward Primer CTACAGGATTGC GAACATCTGATRe verse Primer ACACCATCTGAC GTTGTCTGA		
Slc25a		Forward Primer GAGGCATCGAAA TCTGCATCA Reverse Primer GGATGGAGCCGT AGAGCAA		
Acly		Forward Primer CAGCCAAGGCAA TTTCAGAGC Reverse Primer CTCGACGTTTGAT TAACTGGTCT		
Actb		Forward Primer TACAGCTTCACCA CCACAGC Reverse Primer AAGGAAGGCTGG AAAAGAGC		
**Recombinant DNA**				
**Deposited data**				
NCBI GEO database: GSE297392	https://www.ncbi.nlm.nih.gov/geo/query/acc.cgi?acc=GSE297392			

Software and algorithms				
Prism software 9.3.1	GraphPad by Dotmatics	N/A		
FlowJo 10.8.1	BD Biosciences	N/A		
ViiA 7 Software	Thermo Fisher Scientific	N/A		
HALO image analysis software	Indica Labs			
ZEN 2.3 blue edition software	Zeiss			
ImageJ software	NIH			
**Other**				
Nunc™ 96-Well Polystyrene Conical Bottom MicroWell Plates	Thermo Fisher Scientific	Cat# 249570		
QuadroMACS Separator	Miltenyi Biotec	Cat# 130-090-976		
EasySep Magnet	Stem Cell technologies	Cat# 18000		
50 mL Falcon tubes	Corning	Cat# 352098		
15 mL Falcon tubes	Thomas Scientific	Cat# 12-565-269		
BD FACSymphony	BD	N/A		
ViiA 7 Real-Time PCR System	Thermo Fisher Scientific	N/A		
Glass tubes for serum	Corning (Fisher)	Cat# 8422-50 (05-588-5B)		
Shandon EZ Single Cytofunnel	Fisher Scientific	Cat# A78710020		
Epredia Cytoslide Microscope Slides	Fisher Scientific	Cat# 5991059		
Steriflip-GP Sterile Centrifuge Tube Top Filter Unit	Millipore/Sigma	Cat# SCGP00525		
Falcon round-bottom polypropylene tubes	Falcon	Cat#352063		
Falcon round-bottom polystyrene tubes with cell strainer snap cap, 5mL	Falcon	Cat#352235		

Table S2. Key Resources Table. A detailed listing of reagents or resources, their sources, corresponding identifiers and Abs dilutions.
