# Supplementary material for: Lung environment in healthy old age shapes the phenotype and CCR2-mediated recruitment of a subset of apoptotic, high-turnover alveolar macrophages
Source: Nat Commun. 2026 Oct 7;17:11399. doi: 10.1038/s41467-026-78156-x (PMC13736887; doi:10.1038/s41467-026-78156-x)
Supplement: Supplementary file 2 [file 41467_2026_78156_MOESM2_ESM.pdf]

Description of Additional Supplementary Files

Supplementary Data 1. Differential expression of RNAs in flow cytometry-sorted $CD11c^+CD11b^+$ mAMs versus TRAMs from old mice ($CD11c^+$ $CD11b^-$). (a) Upregulated genes with false discovery rate (FDR) adjusted *p* value <0.05 and $\log_2$ fold change >1. (b) Downregulated genes with false discovery rate (FDR) adjusted *p* value <0.05 and $\log_2$ fold change < -1.

Supplementary Data 2. Differential expression of RNAs in TRAMs in old mice *versus* young mice. (a) Upregulated genes with false discovery rate (FDR) adjusted *p* value <0.05 and $\log_2$ fold change >1. (b) Downregulated genes with false discovery rate (FDR) adjusted *p* value <0.05 and $\log_2$ fold change < -1.
