# Supplementary material for: Lung environment in healthy old age shapes the phenotype and CCR2-mediated recruitment of a subset of apoptotic, high-turnover alveolar macrophages
Source: Nat Commun. 2026 Oct 7;17:11399. doi: 10.1038/s41467-026-78156-x (PMC13736887; doi:10.1038/s41467-026-78156-x)
Supplement: Supplementary file 3 [file 41467_2026_78156_MOESM5_ESM.pdf]

nature portfolio

Corresponding author(s): Larry S Schlesinger and Susanta Pahari

Last updated by author(s): Aug 10, 2026

Reporting Summary

Nature Portfolio wishes to improve the reproducibility of the work that we publish. This form provides structure for consistency and transparency in reporting. For further information on Nature Portfolio policies, see our Editorial Policies and the Editorial Policy Checklist.

Statistics

For all statistical analyses, confirm that the following items are present in the figure legend, table legend, main text, or Methods section.

n/a	Confirmed	
☐	☒	The exact sample size (*n*) for each experimental group/condition, given as a discrete number and unit of measurement
☐	☒	A statement on whether measurements were taken from distinct samples or whether the same sample was measured repeatedly
☐	☒	The statistical test(s) used AND whether they are one- or two-sided<br>*Only common tests should be described solely by name; describe more complex techniques in the Methods section.*
☒	☐	A description of all covariates tested
☐	☒	A description of any assumptions or corrections, such as tests of normality and adjustment for multiple comparisons
☐	☒	A full description of the statistical parameters including central tendency (e.g. means) or other basic estimates (e.g. regression coefficient) AND variation (e.g. standard deviation) or associated estimates of uncertainty (e.g. confidence intervals)
☐	☒	For null hypothesis testing, the test statistic (e.g. *F*, *t*, *r*) with confidence intervals, effect sizes, degrees of freedom and *P* value noted<br>*Give P values as exact values whenever suitable.*
☒	☐	For Bayesian analysis, information on the choice of priors and Markov chain Monte Carlo settings
☒	☐	For hierarchical and complex designs, identification of the appropriate level for tests and full reporting of outcomes
☒	☐	Estimates of effect sizes (e.g. Cohen's *d*, Pearson's *r*), indicating how they were calculated

Our web collection on statistics for biologists contains articles on many of the points above.

Software and code

Policy information about availability of computer code

Data collection	This study did not generate new Code.<br>Flow Cytometry analysis software: BD FACS Symphony instrument was used, and the data were analyzed using FlowJo 10.8.1 software (BD Biosciences).<br>Confocal microscopy software: ZEN 2.3 blue edition (Zeiss) and ImageJ software (NIH, Bethesda, MA).<br>For Luminex assay, data were analyzed using Belysa™ Immunoassay Curve Fitting Software (Millipore Sigma).<br>For RNAseq analysis, read counts for each sample were obtained using featureCounts Software. The heat maps of specific genes were generated using the pheatmap package in R software.<br>Graphs were created and statistical comparisons were conducted using GraphPad Prism version 10 software.
Data analysis	BD FACSymphony multi-color flow cytometer, and compensation, analysis, and data visualization were carried out. Annexin V and EthD-2 cell viability assay analysis was performed by the BD FACS Symphony instrument, and the data were analyzed using FlowJo software.<br>For Flow cytometry cell sorting, BD FACSAria II flow cytometer was used.<br><br>For confocal microscopy analysis, Ms4a3Cre-RosaTdT (Red) and Ccr2gfp/gfp KI/KO (Green) positive cells (BAL and blood derived) stained slides were examined using a Zeiss LSM 800 confocal microscope (20X, 63X magnification). Mean fluorescence intensity (MFI) and percentage of positive cells were quantified using ZEN 2.3 blue edition (Zeiss) and ImageJ software.<br><br>For Luminex assay, the release of cytokines, chemokines, and other secretory factors in the culture supernatant was measured using LLuminex® 100/200™ System (Luminex Corporation). Analytes/mL concentrations were calculated and plotted.

For Chromogenic immunohistochemistry analysis of CD11c+ CD11b+ AMs, whole-slide bright field images were acquired using the Zeiss Axio Scan.Z1 slide scanner (Carl Zeiss, Germany) and quantified using HALO image analysis software (Indica Labs, Albuquerque, NM, USA) and ZEN 2.3 blue edition software.

For bulk RNA-Seq, isolated RNA was quantified using the Qubit 4 Fluorimeter (Invitrogen), and its quality was assessed with the 4200 TapeStation System (Agilent). After Raw sequence mapping, the read counts for each sample were obtained using featureCounts.

The real-time cell metabolism of mouse AMs was measured using a Seahorse XF Extracellular Flux Analyzer XFe96 (Agilent Technologies).

All graphs were created and statistical comparisons were conducted using GraphPad Prism version 10.

For manuscripts utilizing custom algorithms or software that are central to the research but not yet described in published literature, software must be made available to editors and reviewers. We strongly encourage code deposition in a community repository (e.g. GitHub). See the Nature Portfolio guidelines for submitting code & software for further information.

Data

Policy information about availability of data

All manuscripts must include a data availability statement. This statement should provide the following information, where applicable:

- Accession codes, unique identifiers, or web links for publicly available datasets
- A description of any restrictions on data availability
- For clinical datasets or third party data, please ensure that the statement adheres to our policy

All data associated with this study are presented in this paper and are located either in the results section or in the supplementary files. Requests for further details on the dataset, resources, protocols, and reagents can be directed to the corresponding authors. Raw data generated during this study is provided with this paper as a separate Excel file labeled “Source Data file” and in the Supplementary Information. The RNA-seq data generated in this study have been deposited in NCBI’s Gene Expression Omnibus database under accession number: GSE297392 [https://www.ncbi.nlm.nih.gov/geo/query/acc.cgi?acc=GSE297392]. Source data is provided in this paper.

Research involving human participants, their data, or biological material

Policy information about studies with human participants or human data. See also policy information about sex, gender (identity/presentation), and sexual orientation and race, ethnicity and racism.

Reporting on sex and gender	Bronchoalveolar lavage (BAL) samples were collected from healthy adults (20-45 years) and elderly (≥60 years) individuals of both sexes, with an average age of adults (33 years) and elderly (69 years), without discrimination of race or ethnicity, following written consent (see supplemental Table S4). The donors had a preoperative diagnosis of lung nodule, lung nodule with mass, or abnormal parathyroid and thymus, without clinical features. BAL was performed on the healthy lobe of each donor's lung, yielding a cell count range of 2-5 million. Individuals with specific comorbidities and health-related risk factors were excluded from the study to minimize confounding effects. Exclusion criteria included current smoking or smoking cessation within the past month, excessive alcohol use, recreational drug use (injection or non-injection), active pneumonia, asthma, bilateral cancer or ongoing chemotherapy, chronic obstructive pulmonary disease (COPD), prediabetes or diabetes (hemoglobin A1c >5.7% within the previous three months or fasting blood glucose >110 mg/dL), body mass index (BMI) ≥40, hepatitis, human immunodeficiency virus (HIV/AIDS), immunosuppression or use of nonsteroidal anti-inflammatory biologics such as TNF antagonists, leukemia or lymphoma, liver failure, renal failure, rheumatoid arthritis, pregnancy or childbirth within the previous three months, recent antibiotic use, and regular use of over-the-counter anti-inflammatory medications. Each "n" value represents a different human BAL donor as specified in the figure legends.
Reporting on race, ethnicity, or other socially relevant groupings	See above and Table S4: Demographic & Clinical Characteristics of the Participants in this Study
Population characteristics	See above and Table S4: Demographic & Clinical Characteristics of the Participants in this Study
Recruitment	See above and Table S4: Demographic & Clinical Characteristics of the Participants in this Study
Ethics oversight	Human subject studies were conducted in strict accordance with the US Code of Federal and Local Regulations, as overseen by The Ohio State University (OSU) institutional review board with the number 2012H0135. The studies were also overseen by the Texas Biomedical Research Institute with IRB number HSC20170673H. Most samples were collected from the Division of Pulmonary and Critical Care Medicine, UT Health Science Center, San Antonio. All donors provided written consent.

Note that full information on the approval of the study protocol must also be provided in the manuscript.

Field-specific reporting

Please select the one below that is the best fit for your research. If you are not sure, read the appropriate sections before making your selection.

☒ Life sciences ☐ Behavioural & social sciences ☐ Ecological, evolutionary & environmental sciences

For a reference copy of the document with all sections, see nature.com/documents/nr-reporting-summary-flat.pdf

Life sciences study design

All studies must disclose on these points even when the disclosure is negative.

Sample size	The number of mice needed is based on power analysis using data from experiments (website: https://www.stat.ubc.ca/~rollin/stats/ssize/n2.html). The calculations are the customary ones based on normal distributions. The sample size was indicated in each figure legends.
Data exclusions	CD11c-CD11b+ (Flow sorted S3 populations) mouse alveolar macrophages subpopulations in old mice were present in very low numbers and exhibited a transcriptional profile like that of the CD11c+CD11b+ sub populations. Thus, we did not include them in our further analyses in RNAseq study.
Replication	To confirm the reproducibility of our findings, experiments were conducted using at least 4-8 biological replicates or donors, and all replication efforts were successful.
Randomization	In the RNA sequencing study, we randomly selected 10 young and 10 old mice, conducting three replicate experiments to isolate alveolar macrophages and identify their subpopulations using flow cytometry. Other human and mouse donors were also chosen at random.
Blinding	All human, non-human primate, and mouse studies were conducted blindly based on donor number, animal accession number, or experimental group number of mice.

Reporting for specific materials, systems and methods

We require information from authors about some types of materials, experimental systems and methods used in many studies. Here, indicate whether each material, system or method listed is relevant to your study. If you are not sure if a list item applies to your research, read the appropriate section before selecting a response.

Materials & experimental systems

n/a	Involved in the study	
☐	☒	Antibodies
☒	☐	Eukaryotic cell lines
☒	☐	Palaeontology and archaeology
☐	☒	Animals and other organisms
☒	☐	Clinical data
☒	☐	Dual use research of concern
☒	☐	Plants

Methods

n/a	Involved in the study	
☒	☐	ChIP-seq
☐	☒	Flow cytometry
☒	☐	MRI-based neuroimaging

Antibodies

Antibodies used

Human Antibodies

BD Horizon Brilliant Stain Buffer, BD Bioscience, Cat# 563794, RRID: AB_2869750, dilution 1:10
Cell Staining Buffer, BioLegend, Cat# 420201
Human TruStain FcX™ (Fc Receptor Blocking Solution), BioLegend, Cat# 422302, RRID: AB_2818986, dilution 1:50
TruStain FcX™ PLUS (anti-mouse CD16/32) Antibody, BioLegend, Cat# 156604, RRID: AB_2783137, Clone S17011E, dilution 1:50
Brilliant Violet 421™ anti-mouse/human CD11b Antibody, BioLegend, Cat# 101236, RRID: AB_10897942, Clone M1/70, dilution 1:70
Brilliant Violet 421™ Rat IgG2b, κ Isotype Ctrl, BioLegend, Cat# 400640, RRID: AB_10895758, Clone RTK4530, dilution 1:70
PE anti-human CD11c Antibody, BioLegend, Cat#301606, RRID: AB_314176, Clone 3.9, dilution 1:20
PE Mouse IgG1, κ Isotype Ctrl Antibody, BioLegend, Cat#400112, RRID: AB_2847829, Clone MOPC-21, dilution 1:20
Alexa Fluor® 700 anti-human CD11c Antibody, BioLegend, Cat#301648, RRID: AB_314176, Clone 3.9, dilution 1:20
Alexa Fluor® 700 Mouse IgG1, κ Isotype Ctrl Antibody, BioLegend, Cat#400144, RRID: AB_2819923, Clone MOPC-21, dilution 1:20
APC/Cyanine7 anti-human CD11b Antibody, BioLegend, Cat#301342, RRID: AB_2563395, Clone ICRF44, dilution 1:20
APC/Cyanine7 Mouse IgG1, κ Isotype Ctrl Antibody, BioLegend, Cat#400128, RRID: AB_2892538, Clone MOPC-21, dilution 1:20
Brilliant Violet 785™ anti-human CD11c Antibody, BioLegend, Cat# 337252, RRID: AB_3097503, Clone Bu15, dilution 1:20
Brilliant Violet 785™ Mouse IgG1, κ Isotype Ctrl Antibody, BioLegend, Cat# 400170, RRID: AB_11219601, Clone MOPC-21, dilution 1:20
Alexa Fluor® 488 anti-human CD206 (MMR) Antibody, BioLegend, Cat# 321114, RRID: AB_571874, Clone 15-2, dilution 1:20
Alexa Fluor® 488 Mouse IgG1, κ Isotype Ctrl Antibody, BioLegend, Cat# 400129, RRID: AB_2890263, Clone MOPC-21, dilution 1:20
Brilliant Violet 605™ anti-human HLA-DR Antibody, BioLegend, Cat# 307640, RRID: AB_2561913, Clone L243, dilution 1:20
Brilliant Violet 605™ Mouse IgG2a, κ Isotype Ctrl Antibody, BioLegend, Cat# 400270, RRID: AB_3097669, Clone MOPC-173, dilution 1:20
PerCP/Cyanine5.5 anti-human CD16 Antibody, BioLegend, Cat# 302028, RRID: AB_893262, Clone 3G8, dilution 1:20
PerCP/Cyanine5.5 Mouse IgG1, κ Isotype Ctrl Antibody, BioLegend, Cat# 400150, RRID: AB_893680, Clone MOPC-21, dilution 1:20
APC anti-human CD36 Antibody, BioLegend, Cat# 336208, RRID: AB_1279224, Clone 5-271, dilution 1:20
APC Mouse IgG2a, κ Isotype Ctrl (FC) Antibody, BioLegend, Cat# 400222, RRID: AB_2891178, Clone MOPC-173, dilution 1:20
PerCP/Cyanine5.5 anti-human CD64 Antibody, BioLegend, Cat# 305024, RRID: AB_2561585, Clone 10.1, dilution 1:20

PerCP/Cyanine5.5 Mouse IgG1, κ Isotype Ctrl Antibody, BioLegend, Cat# 400150, RRID: AB_893680, Clone MOPC-21, dilution 1:20
Brilliant Violet 605™ anti-human CD86 Antibody, BioLegend, Cat# 305430, RRID:AB_2563824, Clone IT2.2, dilution 1:20
Brilliant Violet 605™ Mouse IgG2b, κ Isotype Ctrl Antibody, BioLegend, Cat# 400350, RRID: AB_3097666, Clone MPC-11, dilution 1:20
Brilliant Violet 605™ anti-human CD192 (CCR2) Antibody, BioLegend, Cat# 357214, RRID: AB_2562702, Clone K036C2, dilution 1:20
Brilliant Violet 605™ Mouse IgG2a, κ Isotype Ctrl Antibody, BioLegend, Cat# 400270, RRID: AB_3097669, Clone MOPC-173, dilution 1:20
PerCP/Cyanine5.5 anti-human CX3CR1 Antibody, BioLegend, Cat# 341614, RRID: AB_11219203, Clone 2A9-1, dilution 1:20
PerCP/Cyanine5.5 Rat IgG2b, κ Isotype Ctrl Antibody, BioLegend, Cat# 400632, RRID: AB_893693, Clone RTK4530, dilution 1:20
PE/Dazzle™ 594 anti-human CD14 Antibody, BioLegend, Cat# 301852, RRID: AB_2629575, Clone M5E2, dilution 1:20
PE/Dazzle™ 594 Mouse IgG2a, κ Isotype Ctrl Antibody, BioLegend, Cat# 400276, RRID: AB_3097694, Clone MOPC-173, dilution 1:20
Alexa Fluor® 488 anti-human CD115 (CSF-1R) Antibody, BioLegend, Cat# 347311, RRID: AB_2566757, Clone 9-4D2-1E4, dilution 1:20
Alexa Fluor® 488 Rat IgG1, κ Isotype Ctrl Antibody, BioLegend, Cat# 400417, RRID: AB_389319, Clone RTK2071, dilution 1:20
PE/Dazzle™ 594 anti-human CD95 (Fas) Antibody, BioLegend, Cat# 305634, RRID: AB_2564221, Clone DX2, dilution 1:20
PE/Dazzle™ 594 Mouse IgG1, κ Isotype Ctrl Antibody, BioLegend, Cat# 400176, RRID: AB_2923261, Clone MOPC-21, dilution 1:20
PerCP-Cy5.5 anti-human CD14, Calibre Scientific, Cat# CAPR-103465, dilution 1:20

Mouse Antibodies

BD OptiBuild™ BUV395 Hamster Anti-Mouse CD95, BD Bioscience, Cat# 740254, RRID: AB_2739999, Clone Jo2, dilution 1:50
BD Horizon™ BUV395 Hamster IgG2, κ Isotype Control, BD Bioscience, Cat# 565820, RRID: AB_2869719, Clone B81-3, dilution 1:50
BD OptiBuild™ BUV496 Rat Anti-Mouse CD192 (CCR2), BD Bioscience, Cat# 750043, RRID: AB_2874260, Clone 475301, dilution 1:50
BUV496 Rat IgG2b, κ Isotype Control, BD Bioscience, Cat# 612954, RRID: AB_2869598, Clone R35-38, dilution 1:50
Brilliant Violet 421™ anti-mouse CD170 (Siglec-F) Antibody, BioLegend, Cat# 155509, RRID: AB_2810421, Clone S17007L, dilution 1:167
Brilliant Violet 421™ Rat IgG2a, κ Isotype Ctrl Antibody, BioLegend, Cat# 400536, RRID: AB_10933427, Clone RTK2758, dilution 1:167
Brilliant Violet 650™ anti-mouse CD3 Antibody, BioLegend, Cat# 100229, RRID: AB_11204249, Clone 17A2, dilution 1:50
Brilliant Violet 650™ Rat IgG2b, κ Isotype Ctrl Antibody, BioLegend, Cat# 400651, RRID: AB_3097674, Clone RTK4530, dilution 1:50
BD OptiBuild™ BV786 Mouse Anti-Mouse CD64 a and b Alloantigens, BD Bioscience, Cat# 741024, RRID: AB_2740644, Clone X54-5/7.1, dilution 1:50
BV786 Mouse IgG1, k Isotype Control, BD Bioscience, Cat# 563330, RRID: AB_2869484, Clone X40, dilution 1:50
Alexa Fluor® 488 anti-mouse/human CD11b Antibody, BioLegend, Cat# 101217, RRID: AB_389305, Clone M1/70, dilution 1:70
Alexa Fluor® 488 Rat IgG2b, κ Isotype Ctrl Antibody, BioLegend, Cat# 400625, RRID: AB_389321, Clone RTK4530, dilution 1:70
PerCP/Cyanine5.5 anti-mouse CD115 (CSF-1R) Antibody, BioLegend, Cat# 135526, RRID: AB_2566461, Clone AFS98, dilution 1:60
PerCP/Cyanine5.5 Rat IgG2a, κ Isotype Ctrl Antibody, BioLegend, Cat# 400532, RRID: AB_2864286, Clone RTK2758, dilution 1:60
PE anti-mouse Ly-6C Antibody, BioLegend, Cat# 128008, RRID: AB_1186132, Clone HK1.4, dilution 1:100
PE Rat IgG2c, κ Isotype Ctrl Antibody, BioLegend, Cat# 400707, RRID: AB_326573, Clone RTK4174, dilution 1:100
PE/Cyanine7 anti-mouse CX3CR1 Antibody, BioLegend, Cat# 149016, RRID: AB_2565699, Clone SA011F11, dilution 1:167
PE/Cyanine7 Mouse IgG2a, κ Isotype Ctrl Antibody, BioLegend, Cat# 400254, RRID: AB_10697032, Clone MOPC-173, dilution 1:167
APC anti-mouse CD45.2 Antibody, BioLegend, Cat# 109814, RRID: AB_389210, Clone 104, dilution 1:60
APC Mouse IgG2a, κ Isotype Ctrl (FC) Antibody, BioLegend, Cat# 400222, RRID: AB_2891178, Clone MOPC-173, dilution 1:60
Alexa Fluor® 700 anti-mouse Ly-6G Antibody, BioLegend, Cat# 127622, RRID: AB_10643269, Clone 1A8, dilution 1:200
Alexa Fluor® 700 Rat IgG2a, κ Isotype Ctrl Antibody, BioLegend, Cat# 400528, RRID: AB_2923249, Clone RTK2758, dilution 1:200
APC/Cyanine7 anti-mouse CD11c Antibody, BioLegend, Cat# 117324, RRID: AB_830646, Clone N418, dilution 1:100
APC/Cyanine7 Armenian Hamster IgG Isotype Ctrl Antibody, BioLegend, Cat# 400927, RRID: AB_2923251, Clone HTK888, dilution 1:100
Brilliant Violet 421™ anti-mouse/human CD11b Antibody, BioLegend, Cat# 101236, RRID: AB_10897942, Clone M1/70, dilution 1:70
Brilliant Violet 421™ Rat IgG2b, κ Isotype Ctrl Antibody, BioLegend, Cat# 400640, RRID: AB_11204247, Clone RTK4530, dilution 1:70
BD OptiBuild™ BV786 Rat Anti-Mouse CD195, BD Bioscience, Cat# 743700, RRID: AB_2741682, Clone C34-3448, dilution 1:50
Rat IgG2c, κ, BD Bioscience, dilution 1:50
PE anti-mouse CD206 (MMR) Antibody, BioLegend, Cat# 141706, RRID: AB_1089575, Clone C068C2, dilution 1:50
PE Rat IgG2a, κ Isotype Ctrl Antibody, BioLegend, Cat# 400508, RRID: AB_326530, Clone RTK2758, dilution 1:50
PE/Cyanine7 anti-mouse CD170 (Siglec-F) Antibody, BioLegend, Cat# 155528, RRID: AB_2890715, Clone S17007L, dilution 1:167
PE/Cyanine7 Rat IgG2a, κ Isotype Ctrl, BioLegend, Cat# 400522, RRID: AB_326542, Clone RTK2758, dilution 1:167
APC anti-mouse CD45.2 Antibody, BioLegend, Cat# 109814, RRID: AB_389210, Clone 104, dilution 1:60
APC Mouse IgG2a, κ Isotype Ctrl (FC) Antibody, BioLegend, Cat# AB_2891178, RRID: 400222, dilution 1:60
APC Rat Anti-Mouse CD45, BD Bioscience, Cat# 559864, RRID: AB_398672, Clone 30-F11, dilution 1:60
APC Rat IgG2b, κ Isotype Control, BD Bioscience, Cat# 553991, RRID: AB_10050405, Clone A95-1, dilution 1:60
Brilliant Violet 650™ anti-mouse F4/80 Antibody, BioLegend, Cat# 123149, RRID: AB_2564589, Clone BM8, dilution1:50
Brilliant Violet 650™ Rat IgG2a, κ Isotype Ctrl Antibody, BioLegend, Cat# 400542, RRID: AB_11123911, Clone RTK2758, dilution 1:50
PerCP/Cyanine5.5 anti-mouse CD45.1 Antibody, BioLegend, Cat# 110728, RRID: AB_893346, Clone A20, dilution 1:80
PerCP/Cyanine5.5 Mouse IgG2a, κ Isotype Ctrl Antibody, BioLegend, Cat# 400258, RRID: AB_10695169, Clone MOPC-173, dilution 1:80
Alexa Fluor® 488 anti-mouse CD45.1 Antibody, BioLegend, Cat# 110718, RRID: AB_492862, Clone A20, dilution 1:100
Alexa Fluor® 488 Mouse IgG2a, κ Isotype Ctrl Antibody, BioLegend, Cat# 400233, RRID: AB_3076443, Clone MOPC-173, dilution 1:100
CellEvent™ Caspase-3/7 Green Flow Cytometry Assay Kit, Thermo Fisher Scientific/Invitrogen, Cat# C10427, dilution 1:80
Caspase-8 p18 Antibody (E-8): sc-166320, Santa Cruz Biotechnology, Cat# sc-166320, Clone E-8, dilution 1:100
Purified NA/LE Hamster Anti-Mouse CD178, BD Bioscience, Cat# 555290, RRID: AB_395708, Clone MFL3, dilution 50 μg
Purified Hamster IgG1, κ Isotype Control, BD Bioscience, Cat# 553969, Clone A19-3, dilution 50 μg
Zombie Green™ Fixable Viability Kit BioLegend Cat# 423112 1:200

APC Annexin V, BioLegend, Cat# 640941, dilution 1:30
Annexin V Binding Buffer, BioLegend, Cat# 422201
PKH26 Red Fluorescent Cell Linker Kit for Phagocytic Cell Labeling, Sigma Aldrich, Cat# PKH26PCL, dilution 10µM (1:10)

Other details in Table S3. Key Resources Table. A detailed listing of reagents or resources, their sources, and corresponding identifiers.

Validation

Antibody validation information for all primary/fluorescence labeled antibodies are listed in the Key Resources table S3 and in the reporting summary documents. Where applicable, antibody validation is referenced from the relevant section of the manuscript, previously published literature, and/or the manufacturer's website, in accordance with the journal's reporting requirements.

Animals and other research organisms

Policy information about studies involving animals; ARRIVE guidelines recommended for reporting animal research, and Sex and Gender in Research

Laboratory animals

Specific pathogen-free C57BL/6 female mice were obtained from Charles River Laboratories (Wilmington, MA) at either 3 months of age (young) or 18 months of age (old) through a contract with the National Institute on Aging (NIA). Mice were housed in individually ventilated cages (IVC) and allowed to acclimate to the facility for one week before being used for the study. C57BL/6 young CD45.1 congenic mice (3 months age), B6.SJL-PtprcaPepcb/BoyJ (B6.CD45.1, Strain # 002014) and CByJ.SJL-Ptprca/J (CD45.1, Strain # 006584) BALB/c mice were purchased from The Jackson Laboratory. Ms4a3Cre-RosaTdT mice bone marrow was acquired from Florent Ginhoux, Singapore Immunology Network (SIgN), Agency for Science, Technology and Research (A*STAR). Ccr2gfp/gfp KI/KO mice (C57BL/6, Strain # 027619) were purchased from The Jackson Laboratory.
Olive baboons (Papio anubis) and Rhesus macaque (Macaca mulatta), both young (ages 3-5 years) and old (ages 15+ years) of both sexes, were obtained from our colony at the Southwest National Primate Research Center (SNPRC) at Texas Biomed.

Wild animals

The study did not involve wild animals.

Reporting on sex

Majority of experiments in mice were conducted in female mice. Olive baboons (Papio anubis) and Rhesus macaque (Macaca mulatta), both young (ages 3-5 years) and old (ages 15+ years) of both sexes, were obtained from our colony at the Southwest National Primate Research Center (SNPRC) at Texas Biomed.

Field-collected samples

The study did not involve sample collection from field

Ethics oversight

All procedures were approved by the Texas Biomedical Research Institute (Texas Biomed) Institutional Laboratory Animal Care and Use Committee (Texas Biomed-IACUC number 1608-MU). Olive baboons (Papio anubis) and Rhesus macaque (Macaca mulatta) were acquired through a biomaterials request, involving opportunistic BAL collection conducted without any disease conditions. This occurred during routine necropsies due to age or through live animal BAL collections, following Texas Biomed-IACUC Protocol number 1516 PC and 1516 MM.

Note that full information on the approval of the study protocol must also be provided in the manuscript.

Plants

Seed stocks

No plants or seeds were used in this study

Novel plant genotypes

No plants or seeds were used in this study

Authentication

No plants or seeds were used in this study

Flow Cytometry

Plots

Confirm that:

- [x] The axis labels state the marker and fluorochrome used (e.g. CD4-FITC).
- [x] The axis scales are clearly visible. Include numbers along axes only for bottom left plot of group (a 'group' is an analysis of identical markers).
- [x] All plots are contour plots with outliers or pseudocolor plots.
- [x] A numerical value for number of cells or percentage (with statistics) is provided.

Methodology

Sample preparation

Bronchoalveolar lavage (BAL) samples were collected from healthy adults aged 20 to 50 years and elderly individuals aged 65

years and older, both male and female. Additionally, samples were obtained from Olive baboons (Papio anubis) and Rhesus macaques (Macaca mulatta), categorized as young (ages 3 to 5 years) and old (ages 15 years and older). C57BL/6 mice were also used, with groups defined as young (3 months old) and old (18 months old). Alveolar macrophages (AMs) were separated from the BAL fluid. A total of 1 to 2 × 10^5 AMs were placed into FACS tubes (Falcon round-bottom polypropylene tubes, Cat# 352063) and centrifuged at 250 x g for 10 minutes. The cell pellets were then resuspended in 50 µL of cell staining buffer from BioLegend (Cat# 420201), along with Human TruStain FcX™ or mouse TruStain FcX™ PLUS (anti-mouse CD16/32) antibody from BioLegend (Cat# 156604) for Fc receptor blocking. The cells were incubated on ice for 30 minutes before being stained with fluorochrome-tagged antibodies and their corresponding isotype-matched control antibodies in BD Horizon Brilliant Stain Buffer Plus (BD, Cat# 566385) for 40 minutes in the dark at 4°C. Following this, the cells were washed once, fixed in 2% paraformaldehyde (Thermo Scientific, Cat# J19943-K2) in cell staining buffer for 10 minutes, and then centrifuged again at 250 x g for 10 minutes. The cells were resuspended in 300 µL of cell staining buffer and filtered through a round-bottom polystyrene tube using a cell strainer snap cap (Falcon, Cat# 352235). Samples were then acquired using a BD FACSymphony multi-color flow cytometer. Compensation, analysis, and data visualization were performed using FlowJo 10.8.1 software (BD Biosciences). Isotype controls and "Fluorescence Minus One" (FMO) controls were utilized as needed to establish gates.

AMs were isolated from both 10 young and 10 old mice and then sorted into CD11c+ CD11b- populations from young and old mice, and CD11c- CD11b+ and CD11c+ CD11b+ populations from old mice using the BD FACSAria II flow cytometer. CD11c-CD11b+ populations were found in very low numbers and displayed a transcriptional signature like the CD11c+CD11b+ populations. Consequently, we did not include them in further analyses. Samples were collected in three different batches as three replicates for bulk-RNAseq. This was achieved by flow cytometry staining and sorting, using BV421 anti-CD11b (clone M1/70) and PE anti-CD11c (clone N418) antibodies. The post-sorted cells were then verified based on the gating strategy, and the purity of the populations was confirmed through gating.

Instrument

BD FACSymphony multi-color flow cytometer and BD FACSAria II flow cytometer

Software

FlowJo 10.8.1 software (BD Biosciences)

Cell population abundance

Human, Olive baboons (Papio anubis), Rhesus macaques (Macaca mulatta) and mice alveolar macrophages (AMs) analyzed. For Bulk RNA-seq study AMs were flow-sorted into sub populations of cells such as CD11c+ CD11b- AMs from young mice and CD11c+ CD11b- and CD11c+ CD11b+ AM sub populations from old mice

Gating strategy

Human alveolar macrophages (hAMs), Olive baboon alveolar macrophages (bAMs), and mouse alveolar macrophages (mAMs) were distinguished by their respective cell surface receptor expressions using flow cytometry. The profiles were as follows: hAMs were characterized as CD206+CD64+CD11c+, bAMs were identified as CD45+CD64+CD206+CD163+CD11c+, and mAMs as CD45+SiglecF+CD11c+. To identify the CD11c+CD11b+ AM subpopulations, cells were pre-gated on the CD45+SiglecF+ (for mAMs) and CD45+CD64+ (for hAMs and bAMs) populations. The predominant group expressing CD11c cell surface receptors were identified as TRAMs. Within this context, CD11c+ CD11b+ subpopulations were recognized as monocyte-derived alveolar macrophages (MoAMs), alongside a very small subpopulation of CD11c- CD11b+ cells present in older mice.

☒ Tick this box to confirm that a figure exemplifying the gating strategy is provided in the Supplementary Information.
