# Supplementary material for: Lung environment in healthy old age shapes the phenotype and CCR2-mediated recruitment of a subset of apoptotic, high-turnover alveolar macrophages
Source: Nat Commun. 2026 Oct 7;17:11399. doi: 10.1038/s41467-026-78156-x (PMC13736887; doi:10.1038/s41467-026-78156-x)
Supplement: Supplementary file 4 [file 41467_2026_78156_MOESM6_ESM.pdf]

nature portfolio

Peer Review File

Lung environment in healthy old age shapes the phenotype and CCR2-mediated recruitment of a subset of apoptotic, high-turnover alveolar macrophages

Corresponding Author: Professor Larry Schlesinger

This file contains all reviewer reports in order by version, followed by all author rebuttals in order by version.

Version 0:

Reviewer comments:

Reviewer #1

(Remarks to the Author)
In this interesting study by Pahari et al, the authors compare BAL obtained from young versus old mice, non-human primates, and humans and perform phenotyping of alveolar macrophage (AM) based on surface markers. They find that compared to their young counterparts, aged lungs across species contain a higher percentage of monocyte-derived AM (MoAM). Using adoptive transfer studies the authors demonstrate that lung-intrinsic signals lead to increased recruitment of monocytes in aged mice. The also confirm that the mechanism of monocyte recruitment (via CCR2), cell turnover (via Fas-mediate apoptosis) and metabolic characteristics of MoAM versus resident AM (TRAM) are similar to those described in models of inflammation. While the presented data lack occasionally lack the appropriate controls to be entirely convincing and the findings are somewhat overstated, these findings have myriad implications for our understanding of aging-related lung disease and immune dysfunction in aging and are likely to be impactful in the field. Suggestions to elevate the rigor of the manuscript are below:

Major comments

1. The high reliance of fluorescent methods raise concern for confounded results throughout the paper. In nearly all cases, AM from old animals (or humans) gain markers (CD11b, TdTomato, MitoSox staining, etc) as measured by fluorescence compared to cells from their younger counterparts. While some of these findings are corroborated at the RNA level, many are not. Given that AM are characterized by autofluorescence in multiple wavelengths, and autofluorescence increases after airborne particulate exposures, it seems possible that measurements may be confounded by increased autofluorescence of AM over the lifespan. Rigorous controls, including FMO should be included in relevant experiments. Likewise, non-fluorescence-based measurements of protein expression would strengthen the findings.
2. The use of SiglecF as a pan-macrophage marker confounds interpretation of flow cytometry data. In studies of pulmonary inflammation, newly recruited MoAM have been shown to initially lack SiglecF and gain expression over time in the alveolus. The use of SiglecF as an initial AM gate throughout the paper may have excluded newly recruited MoAM and may confound interpretation of data, especially in regards to cell recruitment and turnover. (This may also explain the lack of CD11c-CD11b+ cells in mice in Fig 1a compared to baboons and humans in Fig 1b c and e). Use of an alternative pan-macrophage marker (e.g. CD64, F4/80), alternative gating strategy, or discussion of these caveats would strengthen the interpretation of mouse studies.
3. The lack of a comparable CD11c+CD11b+ population in young mice makes interpretation of data in Figures 2 and S8 difficult. It seems likely that the highlighted genes (e.g. “Inflammatory” and “TNF-related”) may simply reflect monocyte-macrophage maturation, rather than a phenotype unique to aging. Comparison to published monocyte-macrophage maturation pathways or post-inflammatory datasets may clarify this issue.
4. The conclusions from Figure 3 are difficult to understand. The authors state that donor TRAMs undergo a “phenotypic change” or are “converted” to CD11c+CD11b+ AM by the aged lung environment. This seems at odds with and less likely (based on their own subsequent data) than the alternative conclusion that they are joined by newly recruited MoAM from the host and that this is unrelated to TRAM. Specific labeling of host and donor cells would strengthen the claims of this experiment. Likewise, it should be stipulated whether BAL samples used for Fig 3f were obtained from control mice or after adoptive transfer as this would impact interpretation of the results.
5. The idea that recruitment of MoAM in aging is caused by or results in inflammation is not supported by the data and not

essential to the key findings of the manuscript. While the authors measured a number of inflammatory markers in the BAL of aged animals, they did not demonstrate that these factors are causally related to monocyte recruitment. Likewise, although MoAM express inflammatory markers, they are also known to be critical for clearance of cell debris, lung repair and regeneration, which may be deemed anti-inflammatory or immunosuppressive functions.

Minor comments:

6. Health status and age differences of human subjects cannot be evaluated based on provided data. Descriptive information regarding human subjects (e.g. median age in each group, number of cells obtained from BAL, impetus for BAL collection) should be added.
7. Cell differentials for baboons should be added to Figure S1.
8. It is not clearly stated whether purified AM or whole BAL were used for extracellular flux assays and qPCR. Since cell differentials were different between old and young mice (Fig S1b) differences in mRNA levels, ECAR and OCR could be confounded by the presence of other cell types and should be discussed.
9. Presentation of CD11b and CD11c expression on transferred CD45.1+ AM in Fig1b would support the stated hypothesis of the experiment.
10. Pictorals are not well-supported by the data. Figure 5h suggests that monocytes are recruited to the pulmonary interstitium, but not the alveolus, in the absence of CCR2. However, interstitial macrophage were not examined in this study. Figure 8 suggests that MoAM bypass a CD11c+CD11b+ transition state to become TRAM in young versus old animals. The data do not support this idea.

Reviewer #2

(Remarks to the Author)

Review Pahari et al.:
The study by Pahari et al. investigates (using RNAseq and flow cytometry) how the healthy aged lung environment shapes the alveolar macrophage pool in humans, baboons and mice. The authors describe an age related, increased occurrence of short-lived, CD11c+ CD11b+ inflammatory monocyte derived alveolar macrophages (moAM). Increased moAM presence in aged mice is due to increased CCR2-dependent moAM recruitment from circulating monocytes and this increased turnover of moAM in aged mice is due to enhanced Fas-mediated signaling. While some of these findings are intriguing, several of the experiments do not fully support the author´s conclusions which strongly diminishes enthusiasm about the study. Importantly, cells are classified using not the most established markers for different AM populations. And most relevant populations were not compared (e.g. young CD11c+CD11b+ were not included in the RNAseq to compare against aged CD11c+ CD11b+ cells, upregulation of CD11b+ on trAM in clodronate experiments does equate to conversion into moAM). In addition, several figures would benefit from improved clarity and labeling.
Details:
Major comments:
- RNAseq comparisons of different AM populations:
Although the authors show convincingly that moAMs in aged mice display increased inflammatory signatures in RNAseq, moAMs from young mice were not sorted, which makes the comparison somewhat skewed as the comparison is not about young versus aged, but about comparing different cell types. Was it not feasible to sort moAM from young animals due to very low numbers? What was the rationale for including old CD11c–CD11b+ cells in the RNAseq, given that these cells were only present in old animals?

The authors also report 311 DEGs between young and old trAM (line 140–141), this finding is not further explored. This is unfortunate, as these DEGs might provide insight into the inflammatory environment of aged lungs and could help explain the increased recruitment of moAM with age.

- CD11b upregulation does not equal moAM lineage
CD11b is an activation marker that can be upregulated in trAM in inflammatory conditions, as the authors themselves demonstrate in their clodronate depletion experiments (Figure 3). While increased CD11b expression on trAM transferred to aged mice does support the idea of a more inflammatory environment, it is misleading to state that these cells “converted to moAM” (line 191-192). These cells are distinct cells from the bona fide monocyte-derived moAM shown in Figure 4. This distinction should be clarified and discussed as it strongly affects the interpretation of the results.
A similar issue arises in Figure 7. After clodronate treatment and FasL NA/LE antibody administration (Figure 7i, j), the authors report decreased percentages of CD11c+ CD11b+ cells. However, because these cells are transferred intratracheally, the reduction could reflect downregulation of CD11b on trAM rather than a true decrease in cell numbers. It would be important, that the transferred cells were labelled with a dye to distinguish their origin from newly bone-marrow recruited cells. Otherwise, the conclusions drawn from this experiment are rather overinterpretations. Moreover, absolute numbers should be shown. If it is truly a decrease in numbers, wouldn´t this imply increased cell death?

- Figure 6: Could the monocyte populations over time in the lungs also be shown and quantified over time? It would be interesting to know if the monocyte are truly recruited prior to day 5 to the lungs and only differentiate into Siglec F+ AM later. From the current FACS plots, the CD45.1+SiglecF+ population never exeeds 0.79%, which does not strongly support this interpretation.

- Figure 7: The authors claim that “enhanced Fas-mediated signaling of resident AMs in old age creates a more favorable

inflammatory environment for the recruitment of monocytes to the lungs" (line 260). However, Fas expression on trAM specifically is not shown. In Figure 7A–B, Fas is measured across all CD11c+ SiglecF+ cells, which in old mice include both tr-AM (CD11c+CD11b–) and moAM (CD11c+CD11b+). Similarly, Figure 1H only shows Fas on moAM. Is Fas expression therefore truly different between trAM from young vs. old mice?

Minor comments
- Figure 2: Populations should be fully labeled as CD11c+CD11b- (old, young) and CD11c+CD11b+ (old), otherwise it´s somewhat confusing, since they all populations are CD11c+.
- Which mAMs were sorted exactly for the metabolic measurements (Fig S10)? Only CD11c+?
- Figure 4b: Is there quantification with more replicates available for Figure 4b?
- How does this compare with the RNA-seq data? In Figure 7D the scheme depicts AM collection at days 3 and 4, while TNF levels are measured at day 5. Was this a separate experiment? This should be clarified in the figure legend and text.

- Generally:
The color scheme across panels should be consistent to aid interpretation. For example, in Figure 7E "green" represents old + anti-FasL NA/LE antibody, while in Figure 7F "green" represents young + anti-FasL NA/LE. This inconsistency makes the figure hard to follow. Gating should clearly indicate whether percentages or absolute cell counts are being reported (e.g. Figures 7K and 7L).

Reviewer #3

(Remarks to the Author)
The study by Pahari et al. found that the aging lung environment drives a shift in the alveolar macrophage (AM) population towards a more inflammatory subset (CD11c+CD11b+). This subset shows upregulated TNF signaling and metabolic shift towards glycolysis. The authors claim that the recruitment of this subset is CCR2-dependent. Also that this subset is monocyte-derived (moAMs) as it's labeled by Ms4a3 Cre. The paper is studying important mechanisms but there are major issues that need to be addressed:
1. The authors did not show that TdT+ TRAMs cells vs TdT-TRAMs have the same transcriptomic signature of CD11c+CD11b+ population. This is essential to confirm that the specific inflammatory signature is a monocyte-derived subset.
2. Figure 3E The authors explain the conversion phenotype with the old lung environment. The authors need to show important young controls- especially clodronate treated young mice after transfer.
3. Overall, clodronate is very non-specific treatment, that introduces alveolar injury and inflammation that could itself drive CD11c+CD11b+ marker induction. It's known to induce neutrophil recruitment, and inflammatory cytokine release.
4. In order to prove monocytic origin, the authors should consider the following experiment: sort Ms4a3-fate mapped AMs (tdT+) at different ages (according to the initial Ms4a3 paper there should be around 20% tdT+ cells around week 10) and perform bulk RNA seq of TdT+ and neg populations and see whether they observe the same differences in transcriptional profiles or if they see upregulation of CD11b in those AMs. It could be that old lung environment make all AMs acquire CD11b and inflammatory state.
5. The authors should make sure they exclude eosinophils as those are SiglecF+ CD11b+ in mice. In most of the plots, CD11c+CD11b+ population rather looks like a smear and not a distinct population.
6. Figure 3e Missing young controls for this experiment and quantification.
7. Lines 203 Since there is already a paper published on DC3 which are labeled with Ms4a3-Cre the text need to be adjusted.
8. Figure S15 Selective images and no quantification, the authors need to quantify by IF or Flow cytometry.
9. Figure 4b Not clear why the authors are focused on CD11b+ population instead of CD11b+ CD11c.
10. Figure 1E, 3C, E Flow plots does not seem to be compensated. This needs to be fixed.
11. Figure 5E The authors should perform a mixed competitive adoptive transfer of monocytes or BM chimera of WT and CCR2 KO into the same recipient to control for inter mouse variability and quantify relative recruitment efficiency. Without a withi mouse competition, differences in recipient inflammation, chemokine gradients, or donor cell preparation across animals could contribute to the observed differences.
12. Overall, adoptive transfer experiments (Figure 4b and Figure 4c,e) establishes the capacity of bone marrow monocytes to engraft and differentiate but it does not prove they are the predominant source during natural aging. Without parabiosis or long-term bone marrow chimeras, the claim of definitive origin remains weak and could be explained by injection of high numbers and non-physiologic conditions.
13. Figure 5b In my opinion there is a clear trend of CX3CR1 increase and I am surprised 3M and 17M is not significant. The authors need to repeat with more mice or change the wording in the text.
14. Figure 4E I cannot see any lineage traced cells (tdT+). Can the authors provide a flow plot with clean tdT+ cells?
15. Figure 4F I am confused by the quatification - tdT+ out of siglecf+ tdT+cells?
16. Figure 7h The authors should be consistent with their quantification readouts. In this plot total AMs are shown instead of CD11b+CD11c+ quantification.
Minor:
• "Our previous research found that CD11c+ CD11b+ mAMs from old mice exhibit elevated levels of cell surface receptors Ly6C and CD115, consistent with a monocytic origin 2. We extended this analysis in mice to examine at what age changes in cell surface receptor expression occur." – but authors did not check Ly6C or CD115 so sentence is not relevant here.

Reviewer #4

(Remarks to the Author)
I co-reviewed this manuscript with one of the reviewers who provided the listed reports. This is part of the Nature Communications initiative to facilitate training in peer review and to provide appropriate recognition for Early Career Researchers who co-review manuscripts.

Version 1:

Reviewer comments:

Reviewer #1

(Remarks to the Author)
Authors have provided thoughtful responses to critiques. Only outstanding issue is as follows:

*In line 217, the assertion that 10-15% of PKHneg cells represent cells that have immigrated into the lung is not substantiated - especially since the authors report that only 85-90% of TRAMs were depleted by clodronate (leaving 10-15% of TRAMs unlabeled). This does not detract from the main messages of the paper. The paragraph would be more clear if "...representing the small TRAM population (10-15%) that had immigrated into the alveolar compartment during this time" was removed from the sentence.

Reviewer #2

(Remarks to the Author)
Pahari et al. have substantially improved the manuscript in response to the previous round of review. In particular, added FMO controls and additional gating validation clearly strengthen the flow cytometry data and address important concerns. The revised version is also much clearer regarding the SiglecF gating strategy and the low abundance of $CD11c^{+}CD11b^{+}$ AMs in young mice.

I support a publication if a few remaining points are addressed:

First, I still think the language around TRAM “conversion” or “reprogramming” should be toned down, especially in the title, abstract, and highlights. The new PKH26-transfer experiment nicely supports that the aged lung environment can induce a $CD11c^{+}CD11b^{+}$ phenotype in transferred TRAMs. However, CD11b upregulation alone does not prove conversion into bona fide monocyte-derived AMs. Pahari et al. could therefore use more cautious wording, such as acquisition of a $CD11c^{+}CD11b^{+}$ phenotype or phenotypic activation/reprogramming.

Second, the Fas mechanism should not be overinterpreted. The data support an association between Fas/FasL signaling and AM turnover in aged lungs, but the current wording still sounds somewhat causal. It would also be helpful if Pahari et al. showed absolute cell numbers in the relevant Fas/FasL antibody experiments, if available, since changes in percentages alone are difficult to interpret.

Third, Pahari et al. should add a short “Limitations of the study” paragraph. This should mention that $CD11c^{+}CD11b^{+}$ AMs from young mice were too rare to sort for RNA-seq, which limits direct age-matched comparison of this population.

Finally, the discussion should also clearly mention that CD11b is not a stable lineage marker. It can be upregulated on AMs upon activation or inflammatory stimulation. Thus, the $CD11c^{+}CD11b^{+}$ phenotype may indicate monocyte origin in some settings, but could also reflect activation-associated CD11b upregulation in resident AMs.

With these textual changes, and ideally the addition of absolute cell numbers for the Fas/FasL experiments, I would consider my remaining concerns addressed.

Reviewer #3

(Remarks to the Author)
No further questions. Congrats on your manuscript

Reviewer #4

(Remarks to the Author)
I co-reviewed this manuscript with one of the reviewers who provided the listed reports. This is part of the Nature Communications initiative to facilitate training in peer review and to provide appropriate recognition for Early Career Researchers who co-review manuscripts.

Open Access This Peer Review File is licensed under a Creative Commons Attribution 4.0 International License, which permits use, sharing, adaptation, distribution and reproduction in any medium or format, as long as you give appropriate credit to the original author(s) and the source, provide a link to the Creative Commons license, and indicate if changes were made.
In cases where reviewers are anonymous, credit should be given to 'Anonymous Referee' and the source.
The images or other third party material in this Peer Review File are included in the article's Creative Commons license, unless indicated otherwise in a credit line to the material. If material is not included in the article's Creative Commons license and your intended use is not permitted by statutory regulation or exceeds the permitted use, you will need to obtain permission directly from the copyright holder.
To view a copy of this license, visit https://creativecommons.org/licenses/by/4.0/

Reviewer's Comments:
Reviewer #1 (Remarks to the Author)

In this interesting study by Pahari et al, the authors compare BAL obtained from young versus old mice, non-human primates, and humans and perform phenotyping of alveolar macrophage (AM) based on surface markers. They find that compared to their young counterparts, aged lungs across species contain a higher percentage of monocyte-derived AM (MoAM). Using adoptive transfer studies the authors demonstrate that lung-intrinsic signals lead to increased recruitment of monocytes in aged mice. The also confirm that the mechanism of monocyte recruitment (via CCR2), cell turnover (via Fas-mediate apoptosis) and metabolic characteristics of MoAM versus resident AM (TRAM) are similar to those described in models of inflammation. While the presented data lack occasionally lack the appropriate controls to be entirely convincing and the findings are somewhat overstated, these findings have myriad implications for our understanding of aging-related lung disease and immune dysfunction in aging and are likely to be impactful in the field. Suggestions to elevate the rigor of the manuscript are below:

Major comments

1. The high reliance of fluorescent methods raise concern for confounded results throughout the paper. In nearly all cases, AM from old animals (or humans) gain markers (CD11b, TdTomato, MitoSox staining, etc) as measured by fluorescence compared to cells from their younger counterparts. While some of these findings are corroborated at the RNA level, many are not. Given that AM are characterized by autofluorescence in multiple wavelengths, and autofluorescence increases after airborne particulate exposures, it seems possible that measurements may be confounded by increased autofluorescence of AM over the lifespan. Rigorous controls, including FMO should be included in relevant experiments. Likewise, non-fluorescence-based measurements of protein expression would strengthen the findings.

Response: *We thank the reviewer for this important comment on the potential influence of autofluorescence regarding fluorescence-based analyses of alveolar macrophages (AMs), particularly in old age samples. We agree that AM autofluorescence is an important consideration and that rigorous controls are required. Our longstanding, optimized flow cytometry strategy aims to minimize autofluorescence-related bias. AM populations were defined using multiparameter gating and marker combinations rather than single-channel intensity alone. In addition, all mice were maintained under specific pathogen-free conditions in individually ventilated cages, minimizing potential confounding from chronic airborne particulate exposure.*

However, as recommended, we performed fluorescence minus one (FMO) controls in addition to isotype controls and have now included these data in the revised manuscript (Figure S2). These controls established comparable gating boundaries and confirmed that the age-associated differences observed were not attributable to nonspecific background or autofluorescence. The relevant figures and Methods section have been updated accordingly.

A practical limitation of AM studies is the low number of cells recoverable from bronchoalveolar lavage (BAL), especially when individual animals are analyzed. In healthy mice, BAL typically yields ~0.6-1 × 10^5 AMs per animal, making flow cytometry and imaging-based approaches the most feasible methods for single-animal phenotypic analysis. In contrast, many bulk protein and gene-expression assays require substantially higher cell numbers. To address this, we complemented fluorescence-based analyses with orthogonal approaches, including BAL fluid protein measurements and RNA-seq using pooled samples, both of which independently supported the phenotypic and inflammatory changes observed in old age AM populations.

In response to the reviewer's suggestion, we have further strengthened the manuscript by adding non-fluorescence-based chromogenic immunohistochemistry (IHC) on isolated BAL cells from young and old mice (new Figure S5a-b). While chromogenic IHC is less sensitive than flow cytometry for resolving rare subsets, it provides independent validation of marker expression without reliance on fluorescence detection.

We appreciate the reviewer's thoughtful suggestions, which have improved the rigor of the revised manuscript.

2. The use of SiglecF as a pan-macrophage marker confounds interpretation of flow cytometry data. In studies of pulmonary inflammation, newly recruited MoAM have been shown to initially lack SiglecF and gain expression over time in the alveolus. The use of SiglecF as an initial AM gate throughout the paper may have excluded newly recruited MoAM and may confound interpretation of data, especially in regards to cell recruitment and turnover. (This may also explain the lack of CD11c-CD11b+ cells in mice in Fig 1a compared to baboons and humans in Fig 1b c and e). Use of an alternative pan-macrophage marker (e.g. CD64, F4/80), alternative gating strategy, or discussion of these caveats would strengthen the interpretation of mouse studies.

Response: *We thank the reviewer for this thoughtful comment regarding the use of SiglecF in our gating strategy and the potential exclusion of newly recruited monocyte-derived alveolar macrophages (MoAMs). We agree that recently recruited MoAMs may initially express low or absent SiglecF and progressively acquire SiglecF expression after entering the alveolar niche, as reported previously (Misharin et al., 2013; Gibbings et al., 2017; Yu et al., 2017). This is an important consideration when interpreting recruitment and turnover dynamics.*

As suggested, we have now evaluated alternative macrophage markers, including CD64 and F4/80, in our flow cytometry panels (Figure S2). These analyses confirmed that the gated populations were macrophages. However, in our hands, CD11c and SiglecF provided the most robust and discrete identification of AMs in BAL. Our analysis demonstrated that $CD45^+F4/80^+$ cells comprised approximately 30% of the BAL population, whereas $CD45^+CD64^+$ cells accounted for more than 70%, reflecting broader expression across pulmonary myeloid populations. In contrast, the most distinct and consistent AM population was identified within the $CD45^+SiglecF^+$ or $CD11c^+SiglecF^+$ gates (96.4%). Therefore, we selected $CD11c^+SiglecF^+$ or $CD45^+SiglecF^+$ cells for characterization of AM populations throughout the study.

Consistent with prior studies, F4/80 expression on mouse AMs is lower and less discrete than on interstitial or bone marrow-derived macrophages, while CD64 expression is broader and more heterogeneous across pulmonary myeloid populations (Gautier et al., 2012; Misharin et al., 2013; Yu et al., 2016). By comparison, $CD45^+SiglecF^+CD11c^+$ cells represented the clearest and most reproducible AM population across experiments (Figure S2). To address the reviewer's concern, we included additional gating validation using CD64 and F4/80 in the revised manuscript (Figure S2), demonstrating concordance with our primary gating strategy. We also clarified the rationale for defining mouse AMs as $CD45^+SiglecF^+CD11c^+$, consistent with widely used approaches in the field (Misharin et al., 2013; Schneider et al., 2014; Gibbings et al., 2017; Kopf et al., 2015).

Importantly, our study was designed to characterize established AM subsets within the alveolar compartment rather than to capture all newly recruited monocytes immediately upon entry. Thus, the use of SiglecF-based gating preferentially identifies cells that have acquired AM features within the alveolar niche. We agree that this approach may underrepresent very early $SiglecF^-$ recruited intermediates, and we have now added this caveat to the Discussion.

We appreciate the reviewer's suggestion, which has strengthened the interpretation and presentation of the mouse flow cytometry data.

3. The lack of a comparable CD11c+CD11b+ population in young mice makes interpretation of data in Figures 2 and S8 difficult. It seems likely that the highlighted genes (e.g. "Inflammatory" and "TNF-related") may simply reflect monocyte-macrophage maturation, rather than a phenotype unique to aging. Comparison to published monocyte-macrophage maturation pathways or post-inflammatory datasets may clarify this issue.

Response: *We thank the reviewer for this insightful comment. We agree that distinguishing an age-specific phenotype from a general monocyte-to-macrophage maturation program is important. The key observation in our study is that the $CD11c^{+}CD11b^{+}$ mAM population was consistently expanded in old mice, whereas it was rare or nearly absent in young mice. In young animals, this subset represented only ~1–2% of BAL cells, with most mice containing <1%, which precluded reliable flow sorting and recovery of sufficient RNA for transcriptomic analysis. Therefore, young mice were included as the biological reference group, while mechanistic comparisons focused on the expanded populations present in old mice.*

To address whether the transcriptional profile reflected aging rather than maturation alone, we compared all three mAM subsets across age groups and evaluated enrichment of a curated literature-based age-associated inflammatory/senescence-related gene set (PMID: 37456831; PMID: 17460174; PMID: 32774895). This analysis identified 29 genes significantly enriched in $CD11c^{+}CD11b^{+}$ mAMs from old mice, including canonical inflammaging mediators (Il1b, Il6, Tnf, Ccl2, Ccr2, S100a8, S100a9), senescence-associated regulators (Cdkn2a), metabolic-inflammatory genes (Hif1a, Acod1, Slc2a1), and AP-1/NF-κB pathway components (Fos, Jun, Junb, Nfkbia, Tnfaip3) (Figure 2d). These pathways are more consistent with chronic inflammatory aging and senescence-like activation than with normal differentiation alone.

We agree that maturation-associated programs may contribute to this phenotype, as recruited macrophages entering the old age alveolar niche likely undergo both differentiation and age-driven inflammatory reprogramming. We have now clarified this point in the Discussion and emphasize that the data support a model in which the old age lung microenvironment promotes the accumulation of recruited macrophages with a distinct inflammaging-like transcriptional state.

4. The conclusions from Figure 3 are difficult to understand. The authors state that donor TRAMs undergo a "phenotypic change" or are "converted" to CD11c+CD11b+ AM by the aged lung environment. This seems at odds with and less likely (based on their own subsequent data) than the alternative conclusion that they are joined by newly recruited MoAM from the host and that this is unrelated to TRAM. Specific labeling of host and donor cells would strengthen the claims of this experiment. Likewise, it should be stipulated whether BAL samples used for Fig 3f were obtained from control mice or after adoptive transfer as this would impact interpretation of the results.

Response: *We particularly thank the reviewer for this important comment and agree that the interpretation of Figure 3 required further clarification and additional experimentation. This was a sticking point for all reviewers. We have spent significant time on this issue and now make clear the fact that the manuscript makes 2 fundamental observations. First, that there are newly recruited MoAMs to the alveolar space from the periphery in old age. Second, that TRAMs undergo a "phenotypic change" (which we now call phenotypic reprogramming) to an increased $CD11c^{+}CD11b^{+}$ state in the old age lung environment. The experiment in the initial manuscript was designed to determine whether the old age alveolar environment can create this phenotypic reprogramming using intratracheally transferred young*

TRAMs in mice. However, we did not clearly distinguish these cells from those recruited in from the periphery creating lack of clarity.

We have conducted a new set of experiments. Following >85–90% AM depletion with intratracheal clodronate liposomes, PKH26-labeled $CD11c^+CD11b^-$ young TRAMs were transferred into young and old recipients. This enabled separation of donor ($PKH26^+$) and host ($PKH26^-$) cells. In old recipients, a substantial fraction of donor $PKH26^+$ AMs acquired a $CD11c^+CD11b^+$ phenotype (~40% at 48 h), whereas this was minimal in young recipients (Figure 3e-g). $PKH26^-$ $CD11c^+CD11b^+$ AMs were also detected. We believe that ongoing recruitment contributes to the 10-15% AM population that is present following clodronate treatment (Figure 3b,c).

Thus, our revised manuscript now makes clear that the old age lung environment promotes both phenotypic reprogramming of TRAMs and recruitment of MoAMs from the periphery. We have revised the text accordingly.

For Figure 3f now h, BAL samples were obtained from the indicated groups, including untreated controls, clodronate-treated mice, and recipients after adoptive transfer. This has now been clarified in the figure legend and Methods.

5. The idea that recruitment of MoAM in aging is caused by or results in inflammation is not supported by the data and not essential to the key findings of the manuscript. While the authors measured a number of inflammatory markers in the BAL of aged animals, they did not demonstrate that these factors are causally related to monocyte recruitment. Likewise, although MoAM express inflammatory markers, they are also known to be critical for clearance of cell debris, lung repair and regeneration, which may be deemed anti-inflammatory or immunosuppressive functions.

Response: *We thank the reviewer for these comments and agree that our data do not establish a direct causal relationship between inflammatory mediators in the old age alveolar space and recruitment of MoAMs (Figure 3h). Our study was intended to define the age-associated expansion and phenotype of recruited macrophage populations rather than to mechanistically prove that specific soluble factors drive recruitment. We have therefore revised the text to avoid causal language and now state that these inflammatory mediators are associated with, rather than demonstrated to cause, the accumulation of MoAMs in old age.*

We also agree that the functional roles of recruited macrophages are complex and cannot be classified simply as pro-inflammatory or anti-inflammatory. In addition to expressing inflammatory markers, MoAMs may contribute to apoptotic cell clearance, tissue remodeling, repair, regeneration, or immunoregulatory processes depending on the local microenvironment. Our data indicate that old age MoAMs exhibit an altered inflammatory transcriptional profile, but this does not exclude concurrent reparative or regulatory functions. We have added this point to the Discussion to provide a more balanced interpretation of the findings.

Minor comments:

6. Health status and age differences of human subjects cannot be evaluated based on provided data. Descriptive information regarding human subjects (e.g. median age in each group, number of cells obtained from BAL, impetus for BAL collection) should be added.

Response: *We thank the reviewer for this helpful suggestion. In response, we have added the requested descriptive information for the human subjects, including age distribution (median/range as appropriate), number of cells obtained from BAL samples (cell yield range as appropriate), and clinical*

indication/impetus for BAL collection. These details are now included in the revised Methods section and corresponding table S4.

7. Cell differentials for baboons should be added to Figure S1.

Response: *As per the reviewer's suggestion, we have added differential cell counts in Baboons (Fig. S1d).*

8. It is not clearly stated whether purified AM or whole BAL were used for extracellular flux assays and qPCR. Since cell differentials were different between old and young mice (Fig S1b) differences in mRNA levels, ECAR and OCR could be confounded by the presence of other cell types and should be discussed.

Response: *We thank the reviewer for this comment. We apologize for the lack of clarity and have revised the Methods section to explicitly state that the extracellular flux (ECAR/OCR) and qPCR analyses were performed using purified AMs isolated from BAL, rather than unfractionated whole BAL cells. Thus, the reported metabolic and transcriptional measurements reflect the total AM population in young versus old mice.*

9. Presentation of CD11b and CD11c expression on transferred CD45.1+ AM in Fig1b would support the stated hypothesis of the experiment.

Response: *We thank the reviewer for this helpful comment. We believe the reviewer is referring to the adoptive transfer experiment shown in Figure 4b (CD45.1$^+$ donor cell transfer), rather than Figure 1b. We agree that presentation of CD11b and CD11c expression on transferred CD45.1$^+$ AMs would further support the experimental rationale and link these findings to the expanded CD11c$^+$CD11b$^+$ population observed in old age mice. We show similar data in Fig 4e, an additional approach after Ms4a3 monocyte transfer. In response, we have revised the figure and added analysis of CD11b and CD11c expression on CD45.1$^+$ donor cells in the recipient BAL. These data are now included in the revised figure and legend.*

10. Pictorals are not well-supported by the data. Figure 5h suggests that monocytes are recruited to the pulmonary interstitium, but not the alveolus, in the absence of CCR2. However, interstitial macrophage were not examined in this study. Figure 8 suggests that MoAM bypass a CD11c+CD11b+ transition state to become TRAM in young versus old animals. The data do not support this idea.

Response: *As per the suggestion, we have modified the figures accordingly. The "monocytes are recruited to the pulmonary interstitium" parts are now removed from Figure 5h and 8.*

Reviewer #2 (Remarks to the Author)

Review Pahari et al.:
The study by Pahari et al. investigates (using RNAseq and flow cytometry) how the healthy aged lung environment shapes the alveolar macrophage pool in humans, baboons and mice. The authors describe an age related, increased occurrence of short-lived, CD11c+ CD11b+ inflammatory monocyte derived alveolar macrophages (moAM). Increased moAM presence in aged mice is due to increased CCR2-dependent moAM recruitment from circulating monocytes and this increased turnover of moAM in aged mice is due to enhanced Fas-mediated signaling. While some of these findings are intriguing, several of the experiments do not fully support the author´s conclusions which strongly diminishes enthusiasm about the study. Importantly, cells are classified using not the most established markers for different AM populations. And most relevant populations were not compared (e.g. young CD11c+CD11b+ were not included in the RNAseq to compare against aged CD11c+ CD11b+ cells, upregulation of CD11b+ on trAM

in clodronate experiments does equate to conversion into moAM). In addition, several figures would benefit from improved clarity and labeling.

Response: *We thank the reviewer for the careful evaluation of our study and for these constructive comments. We agree that several points required clarification and have revised the manuscript accordingly.*

AM populations in mice, humans, and baboons were classified using established combinations of lineage and surface markers commonly used in the field. Specifically, mouse AMs were identified as $CD45^+SiglecF^+CD11c^+$ (Zaynagetdinov et al., 2013; Bain & MacDonald, 2022), human AMs as $CD206^+CD64^+CD11c^+$ (Mitsi et al., 2018; Pahari et al., 2023), and baboon AMs as $CD45^+CD64^+CD206^+CD163^+CD11c^+$ (Cai et al., 2014). In the revised manuscript, we also added validation using alternative macrophage markers (CD64 and F4/80) and clarified the rationale for the selected gating strategies (Figure S2).

We agree that inclusion of young $CD11c^+CD11b^+$ cells for direct RNA-seq comparison would be informative. However, this population was extremely rare in young mice (typically <1-2% of BAL cells), which prevented reliable sorting and recovery of sufficient RNA. We now emphasize this point more clearly and interpret the expanded $CD11c^+CD11b^+$ subset in old mice as an age-associated recruited population rather than assuming direct equivalence to the rare cells observed in young animals.

As further explained in response to R1, we revised the manuscript regarding clodronate adoptive transfer experiments to clarify the main findings of the manuscript and avoid overstating "conversion" in this context. We now clearly describe our main findings as 1) phenotypic reprogramming of intratracheally transferred young TRAMs and 2) recruitment of MoAMs from the periphery in the old age lung environment (Figure 3e).

Finally, we have improved figure clarity throughout the revised manuscript by updating labels, annotations, and color presentation as suggested. We appreciate the reviewer's comments, which have strengthened both the rigor and clarity of the study.

Details:
Major comments:
- RNAseq comparisons of different AM populations:
Although the authors show convincingly that moAMs in aged mice display increased inflammatory signatures in RNAseq, moAMs from young mice were not sorted, which makes the comparison somewhat skewed as the comparison is not about young versus aged, but about comparing different cell types. Was it not feasible to sort moAM from young animals due to very low numbers?

Response: *We thank the reviewer for pointing this out. We agree that inclusion of young $CD11c^+CD11b^+$ cells for direct RNA-seq comparison would be informative. However, this population was extremely rare in young mice (typically <1-2% of BAL cells), which prevented reliable sorting and recovery of sufficient RNA. We now emphasize this point more clearly and interpret the expanded $CD11c^+CD11b^+$ subset in old mice as an age-associated recruited population rather than assuming direct equivalence to the rare cells observed in young animals. These findings support the conclusion that recruitment and accumulation of this subset are features of healthy old age.*

What was the rationale for including old CD11c-CD11b+ cells in the RNAseq, given that these cells were only present in old animals?

Response: *We thank the reviewer for this question. We found the presence of this group to be interesting in old mice, so we elected to include it in our initial transcriptome analysis. However, this subset was*

present at much lower frequency, and its transcriptional profile resembled the CD11c⁺CD11b⁺ population. Because of their low abundance and gene expression pattern, we did not pursue this subset in subsequent analyses. We clarify this in the revised manuscript.

Accordingly, in the end, our primary comparisons focused on the three major mAM populations: CD11c⁺CD11b⁻ mAMs from young mice, CD11c⁺CD11b⁻ mAMs from old mice, and the expanded CD11c⁺CD11b⁺ mAM population from old mice. This section of the manuscript has been modified to clarify our strategy.

The authors also report 311 DEGs between young and old trAM (line 140-141), this finding is not further explored. This is unfortunate, as these DEGs might provide insight into the inflammatory environment of aged lungs and could help explain the increased recruitment of moAM with age.

Response: Thank you for your comments. We agree that the differentially expressed genes (DEGs) between young and old TRAMs may provide important insight into the inflammatory alveolar environment in old age. In the revised manuscript, we have now expanded this analysis by directly comparing CD11c⁺CD11b⁻ TRAMs from young and old mice. The expression profiles of age-associated inflammatory genes are now included in the supplementary data (Figure S10c). These analyses demonstrate that TRAMs from old mice also exhibit inflammatory transcriptional changes, supporting the concept that the old age alveolar environment broadly influences resident AM populations.

-CD11b upregulation does not equal moAM lineage
CD11b is an activation marker that can be upregulated in trAM in inflammatory conditions, as the authors themselves demonstrate in their clodronate depletion experiments (Figure 3). While increased CD11b expression on trAM transferred to aged mice does support the idea of a more inflammatory environment, it is misleading to state that these cells “converted to moAM” (line 191-192). These cells are distinct cells from the bona fide monocyte-derived moAM shown in Figure 4. This distinction should be clarified and discussed as it strongly affects the interpretation of the results.

Response: We thank the reviewer for this helpful comment. We have revised the text regarding the clodronate adoptive transfer experiments to avoid overstating that transferred cells were “converted to MoAMs.” Instead, we now describe these findings as phenotypic reprogramming of transferred cells as noted above and in response to R1.

We performed additional experiments in which donor TRAMs from young mice were labeled with the lipophilic dye PKH26 prior to intratracheal transfer. This approach enabled us to distinguish transferred donor cells (PKH26⁺) from those present in the alveolus (PKH26⁻). Using this strategy, we observed that transferred young TRAMs (PKH26⁺) acquire a CD11c⁺CD11b⁺ phenotype in old recipients.

Accordingly, the revised manuscript clarifies and emphasizes two principal findings:

1. *Old age is associated with increased recruitment of MoAMs into the alveolar space from the periphery.*
2. *The old age alveolar environment promotes phenotypic reprogramming of TRAMs toward a CD11c⁺CD11b⁺ AM state.*

A similar issue arises in Figure 7. After clodronate treatment and FasL NA/LE antibody administration (Figure 7i, j), the authors report decreased percentages of CD11c+ CD11b+ cells. However, because these cells are transferred intratracheally, the reduction could reflect downregulation of CD11b on trAM rather than a true decrease in cell numbers. It would be important that the transferred cells were labelled with a dye to distinguish their origin from newly bone-marrow recruited cells. Otherwise, the conclusions

drawn from this experiment are rather overinterpretations. Moreover, absolute numbers should be shown. If it is truly a decrease in numbers, wouldn´t this imply increased cell death?

Response: *We thank the reviewer for this helpful comment. We agree with the reviewer's interpretation, but we needed new experiments and more clarity in the writing to accurately report the main findings. The new experiments in Figure 3 demonstrate that young PKH26-labeled TRAMs delivered intratracheally to old mice undergo increased phenotypic reprogramming toward a CD11c⁺CD11b⁺ state within the old age alveolar environment.*

*In **Figure 7j**, we show that treatment with a neutralizing FasL NA/LE antibody and young TRAMs transferred following clodronate depletion leads to reduced phenotypic reprogramming to the CD11c⁺CD11b⁺ state. Specifically, the percentage of CD11c⁺CD11b⁺ AMs is less in anti-FasL-treated mice compared with clodrolip-treated controls that received donor young TRAMs.*

In response to the reviewer's suggestion, we quantified the absolute number of CD11c⁺CD11b⁺ AMs and total BAL counts, in addition to their percentage, from the indicated experimental groups. These data are now included in Figure S27 and confirm that FasL neutralization reduces the reprogramming of young TRAMs to the CD11c⁺CD11b⁺ population in old mice. These data suggest that FasL neutralization alters the inflammatory milieu in the old age alveolar space, as evidenced by decreased TNF levels in BAL fluid (Figure 7f), which is associated with reduced phenotypic reprogramming of transferred TRAMs toward the CD11c⁺CD11b⁺ state. We now show that the absolute number of total AMs in old mice does not change (Figure S27c); thus, the reduction in the CD11c⁺CD11b⁺ population following FasL neutralization reflects reduced reprograming of TRAMs rather than a decrease in cell number, consistent with the reviewer's interpretation.

- Figure 6: Could the monocyte populations over time in the lungs also be shown and quantified over time? It would be interesting to know if the monocyte are truly recruited prior to day 5 to the lungs and only differentiate into Siglec F+ AM later. From the current FACS plots, the CD45.1+SiglecF+ population never exeeds 0.79%, which does not strongly support this interpretation.

Response: *Thank you for your question. We are sorry for this confusion in interpreting these data. In Fig 6b, the Q3 population in the FACS plots are CD45.1 cells that are Siglec F negative. Siglec F +CD45.1+ AMs (Q2 in FACS plot) increased over time: 1.34% (Day1), 6.90% (Day3), 16.3% (Day5), then decreased to 4.93% (Day7), 2% (Day10). In the revised version, Q2 is highlighted to emphasize the main finding.*

- Figure 7: The authors claim that "enhanced Fas-mediated signaling of resident AMs in old age creates a more favorable inflammatory environment for the recruitment of monocytes to the lungs" (line 260). However, Fas expression on trAM specifically is not shown. In Figure 7A–B, Fas is measured across all CD11c+ SiglecF+ cells, which in old mice include both tr-AM (CD11c+CD11b–) and moAM (CD11c+CD11b+). Similarly, Figure 1H only shows Fas on moAM. Is Fas expression therefore truly different between trAM from young vs. old mice?

Response: *We thank the reviewer for this important comment and agree that the original manuscript data did not clearly distinguish between Fas expression on TRAMs versus recruited MoAMs. In the revised manuscript, we have now included subset-specific analysis of Fas expression on CD11c⁺CD11b⁻ TRAMs and CD11c⁺CD11b⁺ MoAMs. These new data (Figure 7a,b) demonstrate that Fas expression is increased both on TRAMs and recruited MoAMs in old mice. Accordingly, we have revised the text to clarify that enhanced Fas-mediated signaling in old age involves both resident and recruited AM subsets.*

Minor comments

- Figure 2: Populations should be fully labeled as CD11c+CD11b- (old, young) and CD11c+CD11b+ (old), otherwise it´s somewhat confusing, since they all populations are CD11c+.

Response: *We appreciate your suggestions. We have now modified the figure accordingly.*

- Which mAMs were sorted exactly for the metabolic measurements (Fig S10)? Only CD11c+?

Response: *We thank the reviewer for this question. We apologize for the lack of clarity and have revised the Methods section to explicitly state that the extracellular flux (ECAR/OCR) and qPCR analyses were performed using purified AMs isolated from BAL, rather than unfractionated whole BAL cells. Thus, the reported metabolic and transcriptional measurements reflect the total AM compartment in young versus old mice.*

We agree that age-related shifts in AM subset composition may contribute to these differences. Indeed, this was one of the motivations for our subsequent subset analyses, which identified distinct resident and recruited AM populations in old mice. We considered performing extracellular flux assays on flow-sorted subpopulations (e.g., CD11c$^+$CD11b$^-$ versus CD11c$^+$CD11b$^+$ cells). However, this proved technically challenging because sufficient numbers of viable sorted cells were difficult to recover after BAL collection, antibody staining, prolonged sorting, and immediate Seahorse processing. Despite using large, pooled cohorts (approximately 25-30 mice per group), the extended workflow substantially reduced cell responsiveness in the assay.

We have now clarified this limitation in the Discussion and note that future studies using optimized high-throughput or low-input metabolic platforms will be valuable to resolve subset-specific bioenergetic differences during aging.

- Figure 4b: Is there quantification with more replicates available for Figure 4b?

Response: *To address this question, we now provide quantitative data with more mice in the current version of the manuscript (Fig 4b).*

- How does this compare with the RNA-seq data? In Figure 7D the scheme depicts AM collection at days 3 and 4, while TNF levels are measured on day 5. Was this a separate experiment? This should be clarified in the figure legend and text.

Response: *We thank the reviewer for this helpful comment and apologize for the lack of clarity. The RNA-seq and TNF measurements were performed in related but separate experiments with distinct collection time points. We have now clarified this in the revised figure, figure legend, and main text, including the experimental timeline and sample collection days, to avoid confusion.*

RNAseq data also demonstrate increase in TNF in CD11c+CD11b+ MoAMs in old mice.

- Generally:

The color scheme across panels should be consistent to aid interpretation. For example, in Figure 7E “green” represents old + anti-FasL NA/LE antibody, while in Figure 7F “green” represents young + anti-FasL NA/LE. This inconsistency makes the figure hard to follow. Gating should clearly indicate whether percentages or absolute cell counts are being reported (e.g. Figures 7K and 7L).

Response: *We thank the reviewer for these helpful suggestions. We agree that consistent color schemes are important for figure interpretation. Accordingly, we have revised the color scheme across all relevant panels to ensure that each experimental group is represented uniformly throughout Figure 7. We have*

also updated the gating plots and axis labels to clearly indicate whether values are shown as percentages in panels such as Figures 7k and 7l.

Reviewer #3 (Remarks to the Author):

The study by Pahari et al. found that the aging lung environment drives a shift in the alveolar macrophage (AM) population towards a more inflammatory subset (CD11c+CD11b+). This subset shows upregulated TNF signaling and metabolic shift towards glycolysis. The authors claim that the recruitment of this subset is CCR2-dependent. Also that this subset is monocyte-derived (moAMs) as it's labeled by Ms4a3 Cre. The paper is studying important mechanisms but there are major issues that need to be addressed:

1. The authors did not show that TdT+ TRAMs cells vs TdT-TRAMs have the same transcriptomic signature of CD11c+CD11b+ population. This is essential to confirm that the specific inflammatory signature is a monocyte-derived subset.

Response: *We apologize for the confusion. We have modified the manuscript significantly to make clear that we have two separate but complementary findings in the manuscript: 1) That TRAMs are reprogrammed in the old age alveolar environment to a CD11c+CD11b+ state and 2) MoAMs are newly recruited to the alveolar space in old age from the periphery. Thus, we do not propose that these are the same populations.*

*Although we were not clear on the point regarding TdT+ TRAMs cells vs TdT-TRAMs, we elected to directly compare expression of two key genes, **PPARγ** and **Hif1α**, by qPCR to assess whether TRAMs from Ms4a3^{Cre}-Rosa^{TdT} mice differed from TRAMs of TdT^{-} mice and found no significant differences (see below). Additionally, no transcriptional differences were observed in lung cells or bone marrow cells between the groups.*

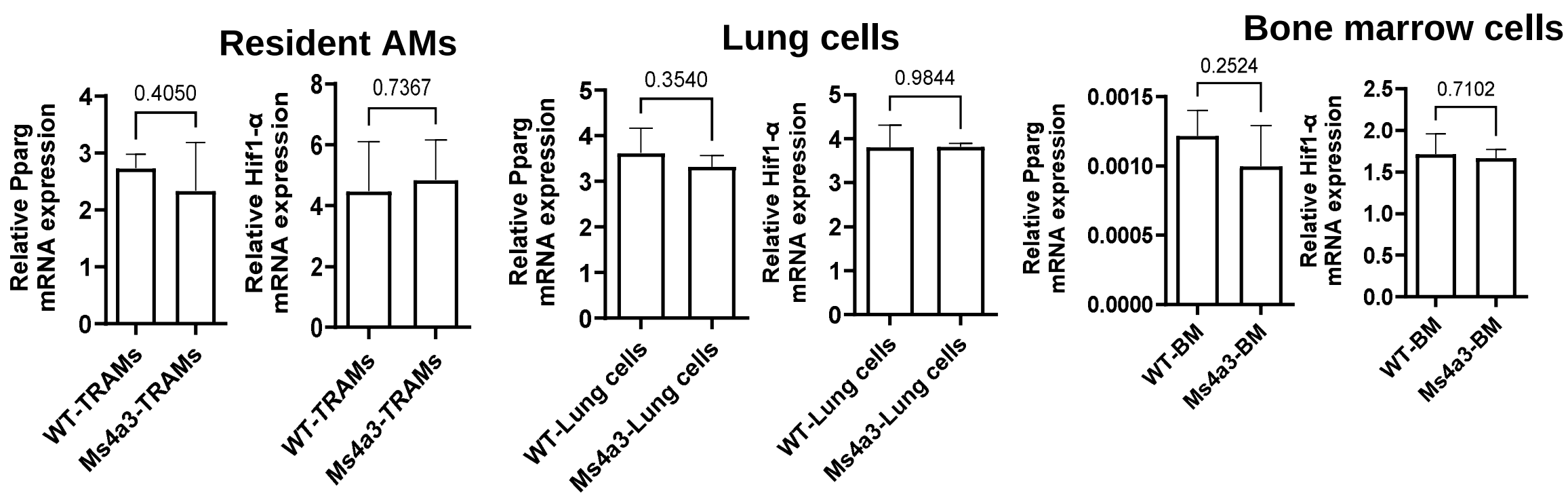

2. Figure 3E The authors explain the conversion phenotype with the old lung environment. The authors need to show important young controls- especially clodronate treated young mice after transfer.

Response: *We thank the reviewer for this helpful comment. We agree that inclusion of young clodronate-treated recipient controls strengthens the interpretation of Figure 3E.*

In response to the reviewer's suggestion (and the other reviewers), we performed additional experiments in which PKH26-labeled young donor AMs were transferred i.t. into both young and old clodronate-treated recipients. This approach allowed us to distinguish transferred donor cells (PKH26^{+}) from AMs present in the alveolus that are PKH26^{-}. Consistent with our model, both i.t. transferred young PKH26^{+} TRAMs and PKH26^{-} MoAMs acquired a sizeable CD11c^{+}CD11b^{+} phenotype in old recipients, whereas this phenotype

was much reduced in young recipients. We have revised the text to clearly separate the two major conclusions of the study:

1. *Old age is associated with increased recruitment of MoAMs into the alveolar space from the periphery.*
2. *The old age alveolar environment promotes phenotypic reprogramming of TRAMs toward a CD11c⁺CD11b⁺ state.*

3. Overall, clodronate is very non-specific treatment, that introduces alveolar injury and inflammation that could itself drive CD11c+CD11b+ marker induction. It's known to induce neutrophil recruitment, and inflammatory cytokine release.

Response: *We thank the reviewer for this comment. We agree that clodronate liposomes can induce transient alveolar perturbation and therefore require careful experimental timing and interpretation. At the same time, intratracheal clodronate liposome administration is a well-established and widely used method for selective depletion of resident AMs in the lung and has been applied extensively to study macrophage repopulation, recruitment, and niche dynamics (Thepen et al., 1989; van Rooijen & Sanders, 1994; Janssen et al., 2011; Misharin et al., 2017). We use a standard approach found in the literature for our studies.*

In our study, clodronate was administered on days 0 and 3, with downstream analyses performed on day 7. Importantly, the age-associated CD11c⁺CD11b⁺ population was also observed in old mice under steady-state conditions, independent of clodronate treatment, indicating that this subset is not solely an artifact of any depletion-induced inflammation (Figure. 3h). The clodronate model was used as a complementary tool to examine how the old age alveolar environment influences macrophage reprogramming (phenotypic plasticity). We have now clarified this rationale in the revised manuscript and Discussion.

4. In order to prove monocytic origin, the authors should consider the following experiment: sort Ms4a3-fate mapped AMs (tdT+) at different ages (according to the initial Ms4a3 paper there should be around 20% tdT+ cells around week 10) and perform bulk RNA seq of TdT+ and neg populations and see whether they observe the same differences in transcriptional profiles or if they see upregulation of CD11b in those AMs. It could be that old lung environment make all AMs acquire CD11b and inflammatory state.

Response: *We thank the reviewer for this thoughtful and technically interesting suggestion. As noted above, we believe that the original manuscript created confusion between our 2 major findings.*

We agree that the proposed age-related bulk RNA-seq experiment would be informative; however, it was not feasible within the scope of the present study. Isolation of sufficient numbers of tdTomato⁺ and tdTomato⁻ AMs from young and especially aged mice would require large cohorts of fate-mapping animals, prolonged aging, and extensive sorting to obtain adequate RNA input. Generating and maintaining aged reporter mice is both time-intensive and costly ($349 per mouse), particularly for the numbers required (35-40) for transcriptomic analyses.

Regarding your last statement: "It could be that old lung environment make all AMs acquire CD11b and inflammatory state." *We agree entirely and, as commented on by all reviewers, we have now enhanced clarity of the manuscript by highlighting the fact that our data demonstrate <u>both</u> enhanced immigration of double positive MoAMs from the periphery <u>and</u> the importance of the alveolar environment in reprogramming TRAMs to a double positive state in in old age.*

5. The authors should make sure they exclude eosinophils as those are SiglecF+ CD11b+ in mice. In most of the plots, CD11c+CD11b+ population rather looks like a smear and not a distinct population.

Response: *We thank the reviewer for this comment. We agree that eosinophils are a relevant consideration in murine lung flow cytometry because they can express SiglecF^{+}CD11b^{+}. To avoid this potential confounder, our analyses were not based on SiglecF and CD11b alone but instead focused on CD11c^{+}SiglecF^{+} cells within the viable CD45^{+} compartment. Because eosinophils are typically CD11c^{-} low, this gating strategy effectively excludes eosinophils while retaining AM populations.*

In addition, eosinophils were infrequent in BAL samples under our steady-state conditions, comprising very low percentages in young mice and generally only ~1-2% in old mice. We have now clarified this gating rationale in the Methods and figure legends.

Regarding the appearance of a "smear" rather than a discrete cluster, we agree that high-density events and biological heterogeneity can broaden distribution patterns in two-parameter plots, particularly in old age samples containing transitional or recruited macrophage states. To improve clarity, we have revised the figure presentation, including axis scaling and labeling, and added representative gating plots in the revised manuscript.

6. Figure 3e Missing young controls for this experiment and quantification.

Response: *We thank the reviewer for this helpful comment. We agree that inclusion of young controls strengthens the interpretation of Figure 3e. In our original experiments, the CD11c^{+}CD11b^{+} population was extremely rare in young mice (typically <1% of BAL cells), whereas the majority of AMs were CD11c^{+}CD11b^{-} TRAMs. For this reason, the initial experiment focused on the effect of the old age alveolar environment.*

Based on this and other reviewer comments, we performed additional experiments including young recipient controls. Donor AMs were labeled with the lipophilic dye PKH26, enabling clear distinction between i.t. transferred donor TRAMs and PKH26- AMs present in the alveolus, followed by quantitative analysis in both young and old recipients. These new data have been incorporated into the revised manuscript, and the corresponding text and figure legends have been updated accordingly.

7. Lines 203 Since there is already a paper published on DC3 which are labeled with Ms4a3-Cre the text need to be adjusted.

Response: *As per the reviewer's suggestion, we modified the text accordingly.*

8. Figure S15 Selective images and no quantification, the authors need to quantify by IF or Flow cytometry.

Response: *We thank the reviewer for this helpful comment. Quantification of Ms4a3-positive cells was provided by both flow cytometry and immunofluorescence in Figure 4C-F. In addition, to further address the reviewer's concern regarding image-based presentation, we have now included quantitative analysis of the supplemental histology data in Figure S19c, which shows the distribution of Ms4a3^{Cre} tdTomato^{+} cells within the lung parenchyma.*

9. Figure 4b Not clear why the authors are focused on CD11b+ population instead of CD11b+ CD11c.

Response: *We are not clear on this comment. However, Fig 4b is intended to show that there is an increase in adoptively transferred CD45.1 monocytes immigrating into the alveolar space (MoAMs) that are double positive (CD11b and CD11c) in old age mice.*

10. Figure 1E, 3C, E Flow plots does not seem to be compensated. This needs to be fixed.

Response: *All are compensated appropriately. As per the reviewer's suggestion, we have now modified the figures with new figures/experiments.*

11. Figure 5E The authors should perform a mixed **competitive adoptive transfer** of monocytes or BM chimera of WT and CCR2 KO into the same recipient to control for inter mouse variability and quantify relative recruitment efficiency. Without a withi mouse competition, differences in recipient inflammation, chemokine gradients, or donor cell preparation across animals could contribute to the observed differences.

Response: *We thank the reviewer for this excellent suggestion. We agree that a mixed competitive adoptive transfer experiment provides a stronger approach to control for inter-mouse variability and directly compares relative recruitment efficiency within the same recipient environment.*

In response, we performed new mixed adoptive transfer experiments using CD45.1$^+$ WT young monocytes mixed together with CCR2-KO GFP$^+$ monocytes, followed by intravenous transfer into young and old recipient mice. BAL and peripheral blood samples were subsequently analyzed by confocal microscopy and quantitative cell assessment (new Figure 5f-h).

These new data, now included in the revised manuscript, demonstrate the relative disadvantage of CCR2-deficient monocytes in recruitment to the old age alveolar space and strengthen our conclusion that CCR2 signaling contributes to monocyte recruitment in old age. The Results and Discussion sections have been revised accordingly.

12. Overall, adoptive transfer experiments (Figure 4b and Figure 4c,e) establishes the capacity of bone marrow monocytes to engraft and differentiate but it does not prove they are the predominant source during natural aging. Without parabiosis or long-term bone marrow chimeras, the claim of definitive origin remains weak and could be explained by injection of high numbers and non-physiologic conditions.

Response: *We thank the reviewer for this thoughtful comment. We agree that adoptive transfer experiments demonstrate the capacity of bone marrow monocytes to engraft and differentiate within the alveolar niche, but do not by themselves establish that they are the exclusive or predominant source of macrophage accumulation during natural aging. We have revised the text to temper this conclusion accordingly. Our intent was to provide complementary evidence for bone marrow contribution using two independent approaches: CD45.1 donor-cell transfer and the Ms4a3^{Cre} Rosa^{tdTomato} fate-mapping model. The Ms4a3 system is particularly informative because labeling is restricted to granulocyte-monocyte progenitors and monocyte-derived lineages, thereby supporting a bone marrow origin for the fate-mapped cells detected in the alveolar compartment. These are extremely challenging experiments, and we tried to address the topic with what was feasible.*

We agree that parabiosis or long-term bone marrow chimera studies would provide additional insight into the relative contribution of circulating precursors during physiological aging. However, we believe that such experiments are beyond the scope of the current study. We have now clarified this point in the Discussion and frame our conclusions more appropriately: our data support a significant contribution of bone marrow-derived monocytes to the expanded macrophage populations observed in old age, while not excluding additional sources or mechanisms.

13. Figure 5b In my opinion there is a clear trend of CX3CR1 increase and I am surprised 3M and 17M is not significant. The authors need to repeat with more mice or change the wording in the text.

Response: *We thank the reviewer for this helpful suggestion. We agree that the original dataset showed a trend towards increased CX3CR1 expression. To address this point, we performed additional experiments comparing young and old mice with an increased sample size. When pooled across multiple*

independent experiments (n = 15 mice/group), CX3CR1 expression remained not significantly different between age groups (new Figure S22b). Based on these additional data, we have revised the text to avoid overinterpretation and now state that CX3CR1 showed a non-significant trend toward an increase with age, whereas CCR2 was more consistently associated with monocyte recruitment into the alveolar space.

14. Figure 4E I cannot see any lineage traced cells (tdT+). Can the authors provide a flow plot with clean tdT+ cells?

Response: *As per the reviewer's suggestion, we reanalyzed and modified the figure included in the revised manuscript.*

15. Figure 4F I am confused by the quatification - tdT+ out of siglecf+ tdT+cells?

Response: *Thank you for pointing this out. We apologize for the lack of clarity. We have now modified and corrected this accordingly. Fig. 4f depicts cumulative data from Fig. 4e, regarding the populations indicated by the red arrow.*

16. Figure 7h The authors should be consistent with their quantification readouts. In this plot total AMs are shown instead of CD11b+CD11c+ quantification.

Response: *We thank the reviewer for this helpful comment. The purpose of this experiment was to determine whether FasL neutralization influences the persistence of intravenously adoptively transferred CD45.1^{+} monocyte-derived cells after recruitment into the alveolar space at day 5. Accordingly, the relevant outcome measure was the maintenance of transferred/recruited donor CD45.1^{+} cells. With antibody treatment, recruited MoAMs were maintained for a longer period relative to the isotype control group (Figure 7h). We have therefore revised the text to clarify that FasL antibody treatment prolonged the persistence of recruited CD45.1^{+} macrophages compared with the isotype control group, consistent with reduced turnover of recruited MoAMs in old mice. This study does not intend to quantify CD11c+CD11b+ populations rather than recruited donor CD45.1^{+} cells. We modified figure 7h Y-axis and Figure 7g regarding the experimental approach, and in the figure legends accordingly to provide more clarity.*

Minor:

- “Our previous research found that CD11c+ CD11b+ mAMs from old mice exhibit elevated levels of cell surface receptors Ly6C and CD115, consistent with a monocytic origin 2. We extended this analysis in mice to examine at what age changes in cell surface receptor expression occur.”– but authors did not check Ly6C or CD115 so sentence is not relevant here.

Response: *We thank the reviewer for this comment. We agree that the wording could be clearer. The statement was intended to reference our previous findings reported by Lafuse et al. (Ref. 2, J Immunol, 2019), in which CD11c^{+}CD11b^{+} mAMs from old mice were shown to express elevated Ly6C and CD115, consistent with a monocytic origin. In the present study, we did not re-evaluate Ly6C or CD115 expression, but instead extended that work by determining the age at which these recruited macrophage populations begin to appear in the BAL. We have revised the text to make this distinction clear.*

REVIEWERS' COMMENTS

Reviewer #1 (Remarks to the Author):

Authors have provided thoughtful responses to critiques. Only outstanding issue is as follows:

*In line 217, the assertion that 10-15% of PKHneg cells represent cells that have immigrated into the lung is not substantiated - especially since the authors report that only 85-90% of TRAMs were depleted by clodronate (leaving 10-15% of TRAMs unlabeled). This does not detract from the main messages of the paper. The paragraph would be more clear if "...representing the small TRAM population (10-15%) that had immigrated into the alveolar compartment during this time" was removed from the sentence.

Response: *As suggested by the reviewer, we have modified the text accordingly.*

Reviewer #2 (Remarks to the Author):

Pahari et al. have substantially improved the manuscript in response to the previous round of review. In particular, added FMO controls and additional gating validation clearly strengthen the flow cytometry data and address important concerns. The revised version is also much clearer regarding the SiglecF gating strategy and the low abundance of $CD11c^{+}CD11b^{+}$ AMs in young mice.

I support a publication if a few remaining points are addressed:

First, I still think the language around TRAM “conversion” or “reprogramming” should be toned down, especially in the title, abstract, and highlights. The new PKH26-transfer experiment nicely supports that the aged lung environment can induce a $CD11c^{+}CD11b^{+}$ phenotype in transferred TRAMs. However, CD11b upregulation alone does not prove conversion into bona fide monocyte-derived AMs. Pahari et al. could therefore use more cautious wording, such as acquisition of a $CD11c^{+}CD11b^{+}$ phenotype or phenotypic activation/reprogramming.

Response: We thank the reviewer for this important comment. We agree that acquisition of CD11b expression alone does not establish “conversion” of TRAMs into monocyte-derived AMs. We have therefore removed the term “conversion” from the manuscript and instead describe our findings as acquisition of a $CD11c^{+}CD11b^{+}$ phenotype or phenotypic reprogramming where appropriate.

Second, the Fas mechanism should not be overinterpreted. The data support an association between Fas/FasL signaling and AM turnover in aged lungs, but the current wording still sounds somewhat causal. It would also be helpful if Pahari et al. showed absolute cell numbers in the relevant Fas/FasL antibody experiments, if available, since changes in percentages alone are difficult to interpret.

Response: We thank the reviewer for this helpful comment. We agree that the Fas/FasL findings should be interpreted cautiously and have revised the manuscript to avoid implying a direct causal relationship between Fas/FasL signaling, AM turnover, and phenotypic changes.

In response to the reviewer’s previous comments, our revised manuscript already included quantification of both the percentage and absolute number of $CD11c^{+}CD11b^{+}$ AMs, together with total AM counts, in the FasL-neutralization experiments (Figure 7j and Figure S27b-c). Following clodronate depletion and intratracheal transfer of young TRAMs into old mice, FasL NA/LE-neutralizing antibody treatment was associated with a reduction in both the percentage and absolute number of $CD11c^{+}CD11b^{+}$ AMs compared with the corresponding control group. Importantly, total AM numbers were not significantly altered (Figure S27c), indicating that the reduction in the $CD11c^{+}CD11b^{+}$ population was not simply attributable to an overall reduction in AM recovery.

Third, Pahari et al. should add a short “Limitations of the study” paragraph. This should mention that $CD11c^{+}CD11b^{+}$ AMs from young mice were too rare to sort for RNA-seq, which limits direct age-matched comparison of this population.

Finally, the discussion should also clearly mention that CD11b is not a stable lineage marker. It can be

upregulated on AMs upon activation or inflammatory stimulation. Thus, the $CD11c^{+}CD11b^{+}$ phenotype may indicate monocyte origin in some settings, but could also reflect activation-associated CD11b upregulation in resident AMs.
With these textual changes, and ideally the addition of absolute cell numbers for the Fas/FasL experiments, I would consider my remaining concerns addressed.

Response: *We thank the reviewer for this helpful suggestion. We have now added a dedicated "Limitations of the Study" paragraph to the Discussion that explicitly addresses comments noted.*

Reviewer #3 (Remarks to the Author):

No further questions. Congrats on your manuscript

We sincerely thank the reviewers for their constructive comments and valuable suggestions, which have significantly improved the quality and clarity of this manuscript.
